# *Zymoseptoria tritici* white-collar complex integrates light, temperature and plant cues to initiate dimorphism and pathogenesis

**DOI:** 10.1038/s41467-022-33183-2

**Published:** 2022-09-26

**Authors:** Sreedhar Kilaru, Elena Fantozzi, Stuart Cannon, Martin Schuster, Thomas M. Chaloner, Celia Guiu-Aragones, Sarah J. Gurr, Gero Steinberg

**Affiliations:** 1 Biosciences, University of Exeter, Stocker Road, EX4 4QD Exeter, UK; 2 University of Utrecht, Padualaan 8, Utrecht, 3584 CH The Netherlands

**Keywords:** Fungal pathogenesis, Pathogens, Cellular microbiology, Plant sciences

## Abstract

Transitioning from spores to hyphae is pivotal to host invasion by the plant pathogenic fungus *Zymoseptoria tritici*. This dimorphic switch can be initiated by high temperature in vitro (~27 °C); however, such a condition may induce cellular heat stress, questioning its relevance to field infections. Here, we study the regulation of the dimorphic switch by temperature and other factors. Climate data from wheat-growing areas indicate that the pathogen sporadically experiences high temperatures such as 27 °C during summer months. However, using a fluorescent dimorphic switch reporter (FDR1) in four wild-type strains, we show that dimorphic switching already initiates at 15–18 °C, and is enhanced by wheat leaf surface compounds. Transcriptomics reveals 1261 genes that are up- or down-regulated in hyphae of all strains. These pan-strain core dimorphism genes (PCDGs) encode known effectors, dimorphism and transcription factors, and light-responsive proteins (velvet factors, opsins, putative blue light receptors). An FDR1-based genetic screen reveals a crucial role for the white-collar complex (WCC) in dimorphism and virulence, mediated by control of PCDG expression. Thus, WCC integrates light with biotic and abiotic cues to orchestrate *Z. tritici* infection.

## Abstract (web summary)

Transitioning from spores to hyphae is crucial for host invasion by the plant pathogenic fungus *Zymoseptoria tritici*. Here, the authors show that the spore-to-hypha transition is enhanced by wheat leaf surface compounds and is regulated by the white-collar complex, which integrates light with biotic and abiotic cues to allow host invasion through open stomata.

## Introduction

Crop losses due to fungal disease pose a significant challenge to food security and the bioeconomy^1^. Wheat is the most widely grown crop globally and accounts for around 20% of human calorie intake^2^. The ascomycete fungus *Zymoseptoria tritici*, the causal agent of the foliar disease Septoria tritici blotch (STB), is the most destructive fungal pathogen of wheat grown in temperate climates. Even with fungicide-based disease mitigation, STB reduces yields of wheat by ~5–10% per annum^3^. Losses caused by *Z. tritici* are thus substantial, accounting for 0.72–1.44 billion Euros per annum in France, Germany and UK, with an additional 0.93 billion Euros spent on fungicides to control STB^3^. Advances in our understanding of the infection biology of *Z. tritici* promise insight into new ways of controlling STB.

In *Z. tritici*, sexual ascospores initiate the primary infection of wheat, whilst secondary infections are caused by asexual spores. The infection cycle begins on the wheat leaf surface, when the spore undergoes a dimorphic transition to a hypha^4^. These tip-growing structures explore the leaf, randomly locating stomata^5^, invading through these apertures and colonising plant tissues^4,6^. Once inside, hyphae secrete effector proteins that modify plant defence^7,8^, as well as plant cell wall-degrading enzymes, thought to provide nutrition for the pathogen^9^. Finally, fruiting bodies (pycnidia) are formed, which release new asexual spores, capable of initiating further infection cycles. During a wheat-growing season, this cycle can be repeated several times^3^, so proliferating the pathogen and causing disease epidemics.

As each infection cycle begins with the dimorphic spore-to-hypha transition, this developmental step is crucial for *Z. tritici* pathogenicity in wheat. In recent years, various *Z. tritici* dimorphism-related proteins have been identified and, in most cases, an effect on the pathogenicity of *Z. tritici* has been shown. These include transcription factors^10^, kinases^11,12^, signalling molecules^13^ and a cyclin^14^. Moreover, *Z. tritici* gene expression depends on light^15^ and light-dependent velvet factors are implicated in the dimorphic transition^16,17^. However, our understanding of the dimorphic switch in *Z. tritici* remains fragmentary.

Many human pathogenic fungi transition morphologically in response to temperature changes^18^. This seems rare in plant pathogenic fungi, where external cues such as pH, plant surface waxes or pheromones initiate transitions^19^. An exception is *Z. tritici*, which forms hyphae in vitro upon shifting to 25–28 °C^13,17,20^. However, this elevated temperature is thought to induce cellular heat stress^21–23^ and was shown to impact genome stability and mutation rates^22,24^. As yield-reducing infection of the flag leaf by *Z. tritici* occurs in May (https://ahdb.org.uk/knowledge-library/septoria-tritici-in-winter-wheat), where temperatures are expected to be lower, the relevance of high temperatures in field infections is in doubt.

Here, we address the role of temperature in *Z. tritici* dimorphic switching and virulence. Monitoring dimorphic switching at the cellular level, using a fluorescent reporter, reveals that even moderate temperatures induce the hyphal growth programme. Moreover, exogenous addition of wheat leaf surface compounds enhances hyphal switching to promote pathogenesis. Genetic screening reveals the light-sensing white-collar complex (WCC) as a master regulator of dimorphic switching that controls the expression of most dimorphism core genes, identified in four *Z. tritici* wildtype (WT) strains. We show that asynchronous light conditions impair pathogenicity and discuss a proposed role for the WCC in aligning pathogenic development with plant stomatal opening to optimise infection success.

## Results

### Hyphal growth is induced by temperatures that cause heat stress

**Fig. 1:**
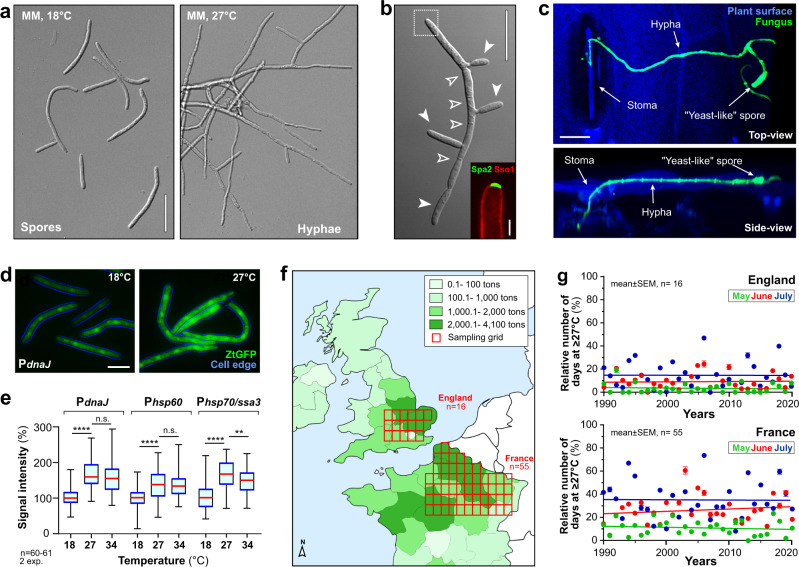
Temperature and dimorphic switching in *Z. tritici*. **a** Spores and hyphae, at 18 and 27 °C, in minimal medium (MM). Scale bar = 25 µm. **b** *Z. tritici* spore undergoing microconidiation. Multi-cellular spore (septa indicated by open arrowhead) form new asexual spores (=blastospores, filled arrowheads); a growing tip is shown (dotted box in overview; inset, where plasma membrane labelled with mCherry-Sso1 in red and the polarisome marked by ZtSpa2^26^ in green). Scale bars = 20 µm (overview); 2 µm (inset, lower right corner). See Supplementary Fig. 1. **c** *Z. tritici* on wheat leaf surface. A hypha emerges from a yeast-like spore and enters via stomata. Plant auto-fluorescence (blue), fungal cell wall stained with WGA-Alexa-Fluor 488 (green). Scale bar = 20 µm. See Supplementary Movie 2. **d** IPO323 cells, expressing codon-optimised green-fluorescent protein (ZtGFP) under the promoter of a gene encoding a homologue of the heat stress chaperone DnaJ (P_*dnaJ*_, green; FungiDB gene ID: ZTRI_8.716) promoter. The cell edge is indicated in blue. Fluorescence signal intensity increases at 27 °C. Scale bar = 10 µm. **e** Signal intensity in IPO323 cells, expressing ZtGFP under promoters of genes, encoding putative heat stress proteins DnaJ/Hsp40/Sis1 (P_*dnaJ*_), Hsp60 (P_*hsp60*_) and Hsp70/Ssa3 (P_*hsp70/ssa3*_). FungiDB gene IDs are: DnaJ/Hsp40/Sis1, ZTRI_8.716; Hsp60, ZTRI_8.689; Hsp70/Ssa3, ZTRI_10.182. All promoters are induced at 27 and 34 °C. *n* = 60–61 cells examined over 2 independent experiments. See Supplementary Fig. 2a and Supplementary Table 4. **f** Map showing areas of intensive wheat cultivation in UK and France. Colour coding indicates average yield per year in tons from 2010 to 2014. Sampling areas, each approximately 2400 km^2^, indicated in red. Yield data were obtained from USDA (https://ipad.fas.usda.gov/rssiws/al/crop_production_maps/Europe/EU_Wheat.png). Grid location and size match satellite temperature data, obtained from https://rda.ucar.edu/datasets/ds628.0/. **g** Relative number of days in May, June, and July when wheat field temperature in England (upper graph) and France (lower graph) reached ≥27 °C (1990–2020). Areas of temperature sampling indicated by red boxes in (**f**). Note repeated cycles of infection occur during the growing season. Results shown in (**a**–**d**) were obtained independently in 2 (**d**) or >5 experiments (**a**–**c**). Data points in (**g**) show mean ± SEM, with *n* = 16 (England) or 55 (France) measurements in different area grids, depicted in (**f**). Some data sets in (**e**) are non-normally distributed (P*dnaJ*, 34 °C; P*hsp60*, 34 °C Shapiro–Wilk test, *P* < 0.05; see Source Data file); thus, data are shown as Whiskers’ plots with 25/75 percentiles (blue line) and median (red line; for all numerical values see Source Data file); statistical comparison in (**e**) used Mann–Whitney testing, comparisons are indicated by brackets; n.s. = non-significant difference at two-tailed *P* = 0.0764 (left comparison) and two-tailed *P* = 0.8464 (right comparison); **significant difference at two-tailed *P* = 0.0034; ****significant difference at two-tailed *P* < 0.0001. Source data are provided as a Source Data file.

We began our study with a characterisation of the morphologies underpinning dimorphism in *Z. tritici*. At 18 °C liquid cultures (minimal medium, MM), reference strain IPO323^25^ formed multi-cellular structures (Fig. 1a), that multiplied by lateral budding and are therefore defined as blastospores (hereafter named spores; Fig. 1b). After 2 days at 27 °C branched hyphae dominated the culture (Fig. 1a). Spores had an apical growth point, indicated by polarisome protein ZtSpa2^26^ (Fig. 1b, inset), and occasionally grew into curved hyphal-like cell chains that were wider than the straight-growing hyphae (Supplementary Fig. 1a). Measuring cell length and width, in cells expressing plasma membrane marker eGFP-Sso1^27^ (see Supplementary Tables 1–3 for strain genotype, plasmid description and experimental usage), revealed that axenic hyphae have the same cell dimensions as plant-colonising hyphae (Supplementary Fig. 1b–d and Supplementary Movie 1). Such hyphae enable *Z. tritici* invasion via stomata (Fig. 1c and Supplementary Movie 2) and are thus likely to represent the plant-colonising morphotype (Supplementary Fig. 1e and Supplementary Movie 3).

While 27 °C is widely used to induce hyphal growth in vitro^13,17,20^, this elevated temperature may heat stress the cell^21–23^ and may not be relevant for infection in the field. Heat stress in fungi is accompanied by the expression of chaperones^28^. We identified homologues of *Aspergillus fumigatus* chaperones, shown to be induced after 2–3 h exposure to heat^29^ (Supplementary Table 4). We fused 1000 bp of their respective promoters to the gene, encoding *Z. tritici* codon-optimised GFP (ZtGFP) and integrated constructs into the IPO323 *sdi1* locus^30^. After 3 h at 27 or 34 °C, all promoters were induced, resulting in increased cytoplasmic green fluorescence (Fig. 1d, e and Supplementary Fig. 2a). Thus, our results support the idea that 27 °C imposes heat stress on *Z. tritici*.

We next asked if such high temperatures are relevant in wheat fields. To address this, we extracted canopy temperatures in sample areas of ~60 × 40 km for wheat-intensive growing regions in England and France (Fig. 1f) for May, June and July, over 30 years, from climate reanalysis data^31^. This revealed that temperatures reached ≥18 °C during most days from May to July (Supplementary Fig. 2b). Days with ≥27 °C were less common but occurred in France and England in June and July (Fig. 1g). As *Z. tritici* passes several cycles of infection during the growing season^3^, the pathogen likely experiences such heat stress-inducing temperatures in wheat fields.

### A fluorescent reporter system to investigate spore-to-hypha transition

**Fig. 2:**
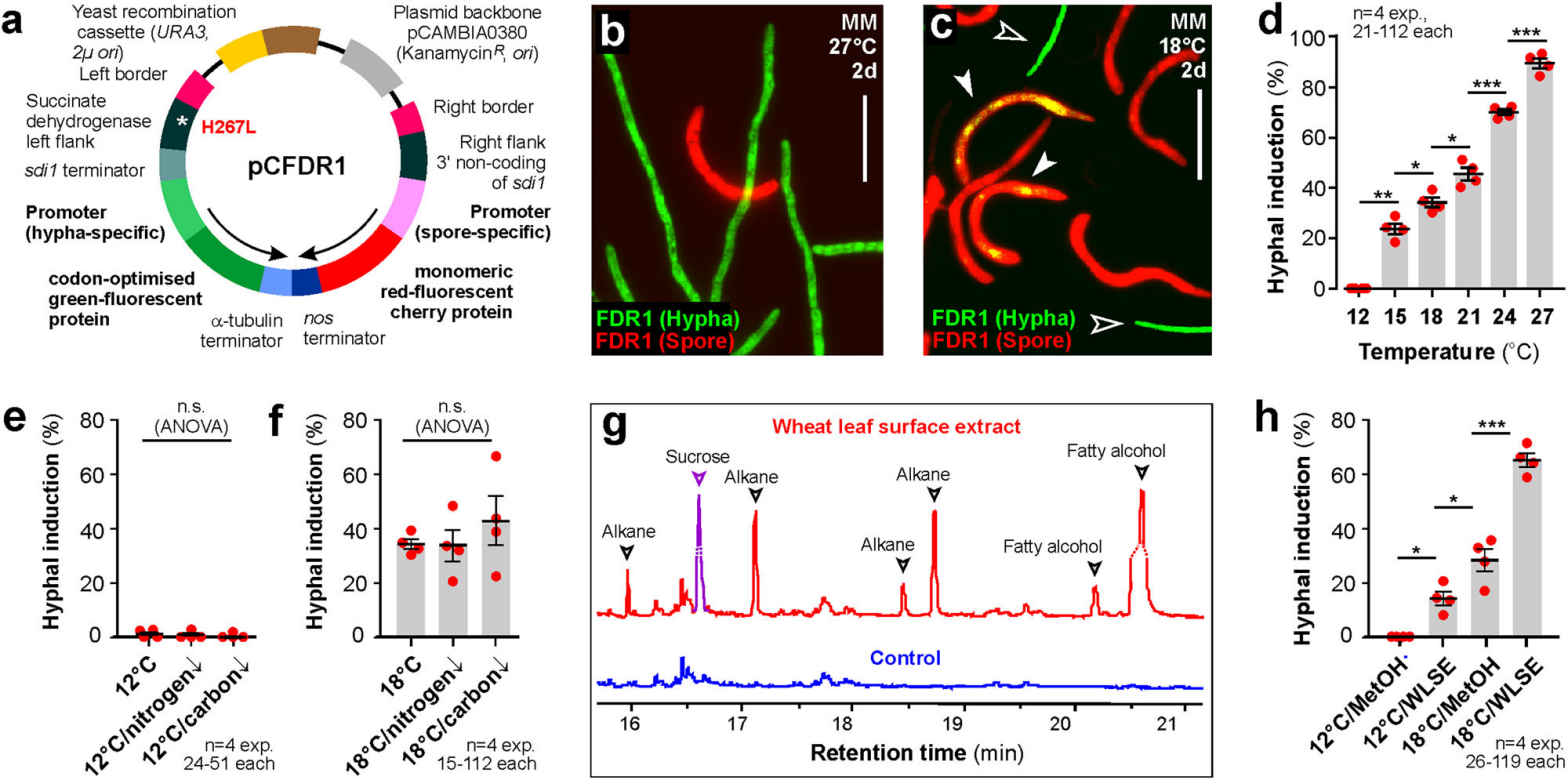
A fluorescent reporter to analyse dimorphic switching conditions. **a** Organisation of the reporter plasmid pCFDR1. Expression of mCherry and ZtGFP is driven by 1000 bp promoters from spore and hypha-specific genes. Transcription direction indicated by arrows. The hygromycin resistance-conferring point mutation is indicated by asterisk and H267L. For nucleotide sequence for the spore- and hyphal-specific promoters see Source Data File. **b** IPO323 cells expressing pCFDR1 (IPO323_FDR1) after 2 days of growth in MM at 27 °C. Hyphae express codon-optimised ZtGFP (green), whereas spores express mCherry (red). Scale bar = 30 µm. See Supplementary Fig. 3. **c** IPO323_FDR1 after 2 days in MM/18 °C. Few hyphae are formed (open arrowhead, green); some spores show expression of ZtGFP (yellow in overlay; closed arrowhead), suggesting they are transitioning to hyphae. Scale bar = 30 µm. See Supplementary Fig. 3. **d** Hyphal programme induction, visualised by ZtGFP expression in IPO323_FDR1, after 2-day growth in MM at increasing temperatures. See also Supplementary Fig. 3. **e**, **f** Hyphal programme induction, visualised by ZtGFP expression in IPO323_FDR1, grown for 2 days in carbon- and nitrogen-free minimal medium at 12 °C (**e**) and 18 °C (**f**). Control experiments used minimal medium. **g** GC-MS spectra of wheat leaf surface extract (WLSE) and solvent alone (Control). WLSEs contain several compounds that are absent from the control spectrum (arrowheads). The sucrose peak (purple) was manually added from the total ion chromatogram. See also Supplementary Fig. 5a, b. Mass spectra of hydrocarbon-related compounds are included in the Source File. **h** Hyphal programme induction, visualised by ZtGFP expression in IPO323_FDR1 after 2-day growth in MM at 12 and 18 °C, supplemented with the solvent methanol (12 °C/MetOH, 18 °C/MetOH), or WLSE (12 °C/WLSE and 18 °C/WLSE). Note that WLSE alone induces ZtGFP expression in ~15% and 18 °C alone in ~28% of all spores, yet both cues together reach ~65%, suggesting they act synergistically. Results shown in (**b**, **c**) were obtained independently in 4 experiments. Data in (**d**–**f**, **h**) are given as mean ± SEM, with *n* = 4 biologically independent experiments, each with 21–112 (**d**), 24–51 (**e**), 15–112 (**f**) or 29–119 (**h**) structures (=spores or hyphae) examined; red dots represent independent experiments; statistical comparison used Student’s *t*-testing with Welch correction; *significant difference at two-tailed *P* = 0.0105 (**d**, comparison 15–18 °C) and 0.012 (**d**, comparison 18–21 °C), 0.0121 (**h**, comparison 12 °C/MetOH to 12 °C/WLSE) and 0.0326 (**h**, comparison 18 °C/MetOH to 12 °C/WLSE); **significant difference at two-tailed *P* = 0.0015 (**d**); ***significant difference at two-tailed *P* = 0.0006 (**d**, comparison 21–24 °C), 0.0004 (**d**, comparison 24–27 °C) and 0.0006 (**h**). **e**, **f** used one-way ANOVA testing; n.s. = non-significant difference at two-tailed *P* values of 0.8374 (**e**) and 0.5045 (**f**). Source data are provided as a Source Data file.

We noticed that IPO323 spores culture grown in MM for 2 days at 18 °C, contained ~6% morphologically recognisable hyphae. Therefore, we tested if this moderate temperature induces low levels of dimorphic switching. To carry out such cellular analysis, we developed a fluorescent dimorphic reporter (FDR1) to facilitate early detection of the spore-to-hypha transition, with a change from red fluorescence (in spores) to green fluorescence (in cells that entered the hyphal programme). We purified RNA from spores and hyphae and identified genes, highly expressed in spores (ZTRI_7.69; all IDs from FungiDB, https://fungidb.org/fungidb/app/record/dataset/NCBITAXON_336722) or hyphae (ZTRI_13.167). We fused their promoters (1000 bp; nucleotide sequence provided in the associated Source File) to genes, encoding mCherry and ZtGFP, respectively. Both constructs were combined in the plasmid pCFDR1 (Fig. 2a) and integrated into IPO323 *sdi1* locus (strain IPO323_FDR1). When grown at 27 °C in MM the resulting strain IPO323_FDR1 showed green-fluorescent hyphae, which could readily be distinguished from red-fluorescent spores (Fig. 2b and Supplementary Fig. 3). We grew strain IPO323_FDR1 for 2 days in MM at 18 °C, shaking at 200 rpm (for details see Methods). This was followed by assessing whether red-fluorescent spores also express green-fluorescent ZtGFP, indicating induction of the hyphal programme. We found that ~30% of all spores co-expressed mCherry and ZtGFP (Fig. 2c; visible as yellow spores, image overlay; Fig. 2d), despite not yet having formed a morphological hypha. Thus, we concluded that 18 °C induced dimorphic switching. We investigated the effect of higher and lower temperatures on hyphal induction, monitored by FDR1, after growth for 2 days in MM. This revealed that dimorphic switching occurred at 15 °C and increased with temperature (Fig. 2d and Supplementary Fig. 3a). At 12 °C, cells did not switch (Fig. 2d and Supplementary Fig. 3a), but still grew with a doubling time of 11.0 h (compared to 4.2 h doubling time at 18 °C; Supplementary Fig. 4). In the UK, the average temperature in May, when flag leaf infection occurs, is 11.84 °C (years 2015–2021; https://www.statista.com/statistics/322658/monthly-average-daily-temperatures-in-the-united-kingdom-uk/). Thus, we consider 12 °C a field-relevant temperature for *Z. tritici*.

Our data showed that dimorphic switching does not occur at 12 °C. We therefore used FDR1-containing cells, grown at this temperature, to identify cues that initiate dimorphic switching. Previous work demonstrated that a combination of carbon- and nitrogen-free MM and 18 °C induces hyphal growth^21^. Thus, we considered nutrient limitation a possible cue for dimorphic switching. However, when cells of strain IPO323_FDR1 were grown for 2 days at 12 °C in carbon- or nitrogen-free MM, no significant increase in GFP expression was detected (Fig. 2e). We next tested if the combination of 18 °C and nutrient limitation induces hyphal growth. Again, we did not find an increase in dimorphic switching when compared to control experiments at 18 °C alone (Fig. 2f). Finally, we tested if nutrient limitation induces hyphae that do not express GFP and therefore remain undetected by FDR1. We did not, however, find any morphological hyphae that did not express GFP. Thus, we conclude that, under the conditions tested here, carbon- and nitrogen-depletion does not induce hyphal switching.

Next, we speculated that the leaf surface could provide additional cues for hyphal growth, as spores rest for several hours on the leaf surface before they undergo the yeast-hypha transition^32^. We aimed to understand the timing of dimorphic switching in planta by inoculating spores of strain IPO323_FDR1, grown in MM at 12 °C, onto wheat leaves at 18 °C and hourly monitored the appearance of GFP in cells. We found a continuous increase of GFP over time, with first spores switching at 1 h and ~45% of all spores expressing GFP at 5 h after inoculation (Supplementary Fig. 3b, c). These data suggest that dimorphic switching is a rapid response to cues on the plant surface.

To gain insight into the nature of such cues, we washed wheat leaves with chloroform and analysed the resultant wheat leaf surface extract (WLSE; see Methods) by gas chromatography/mass spectrometry (GC/MS) to identify organic acids, amino acids, sugars, fatty acids and hydrocarbon-related compounds^33^. Comparison of the total ion chromatograms of the solvent-containing blank and WLSE identified several specific peaks. Subsequent mass spectral analysis, followed by library searches revealed the presence of glycerol, sucrose, an unidentified compound (Supplementary Fig. 5a), several alkanes and long chain fatty alcohols (Fig. 2g; see Supplementary Fig. 5b for profiles). Intracellular metabolites, such as organic acids and amino acids were absent, suggesting that we had not disrupted plant cells.

We next investigated if WLSE, dissolved in methanol, induces the dimorphic transition from spores to hyphae. We grew cells at 12 °C, where no expression of GFP from the FDR1 plasmid was found (0%; in these control experiments 0.5% (v v^−1^) of the solvent methanol (MetOH) alone was added). When the solvent was replaced by 0.5% (v v^−1^) WLSE, 14.05 ± 5.15% of the spores showed GFP expression, indicating that they had entered the hyphal growth programme (Fig. 2h). Thus, WLSE alone was able to induce some dimorphic switching. At 18 °C/MetOH, 28.25 ± 8.23% switched to the hyphal growth, but when 18 °C was combined with WLSE (18 °C/WLSE), dimorphic switching occurred in 65.13 ± 5.22% of all spores. This number clearly exceeds the sum of the individual stimuli (42.30%), suggesting that WLSE and 18 °C cooperate synergistically to induce hyphal growth in *Z. tritici*. In summary, we consider it likely that leaf surface compounds and temperature provide independent cues, which cooperate to induce hyphal growth on the leaf, thereby enabling *Z. tritici* to invade leaves.

### Temperature and plant cues induce dimorphic switching in other isolates

All results thus far were obtained with *Z. tritici* WT strain IPO323, yet variations between this reference and other WT strains have been reported^34,35^. We therefore included the previously studied isolate ST99CH_1E4 (= 1E4; isolated in Switzerland^34^), and two unpublished strains, MC2306 and T5 (isolated in the UK and North America, respectively). All strains caused disease symptoms in wheat cultivar Consort leaves, albeit with fewer fruiting bodies (pycnidia) in T5 infections (Supplementary Fig. 6). To confirm that strains MC2306 and T5 are *Z. tritici* isolates, we sequenced their genomic DNA and randomly selected 10,000 gDNA sequence reads (150 bp). Comparing these against the non-redundant database, using nBLAST, revealed that 97% (MC2306) and 98% (T5) of all fragments aligned best with *Z. tritici* entries. Thus, we conclude that T5 and MC2306 are *Z. tritici* isolates.

**Fig. 3:**
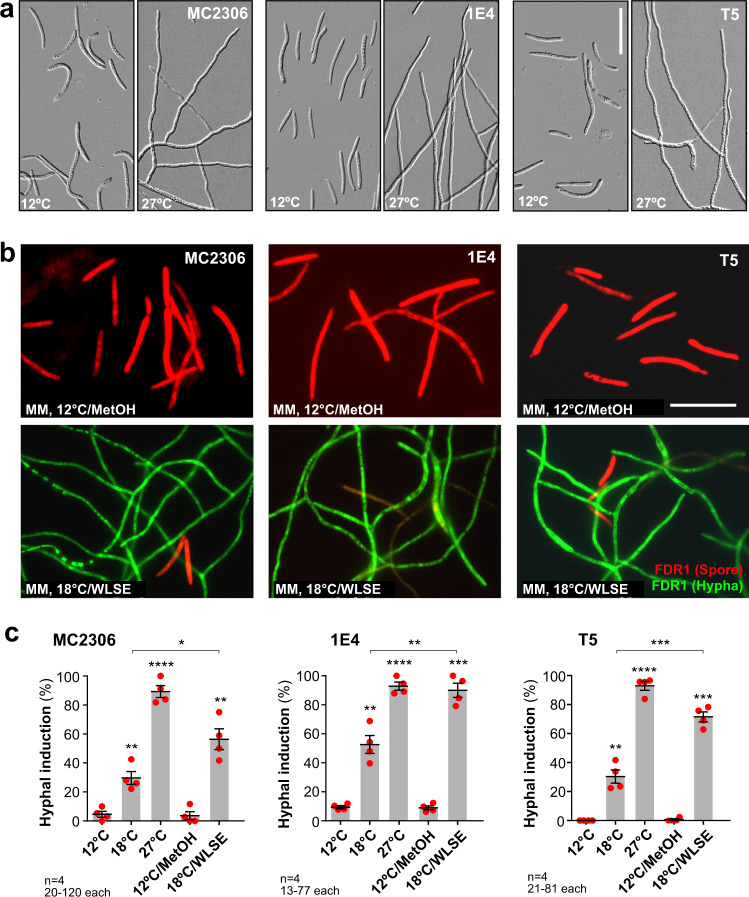
Response of alternative wildtype strains to environmental cues. **a** Morphology of strains MC2306, 1E4 and T5 in MM at 12 and 27 °C. All strains respond to higher temperatures by forming hyphae. Scale bar = 30 µm. **b** Expression of FDR1-encoded fluorescent proteins in wildtype strains MC2306, 1E4 and T5. All strains express mCherry (red) in MM at 12 °C/MetOH but switch to ZtGFP-containing hyphae (green) after 3 days at 18 °C/WLSE. Scale bar = 20 µm. **c** Quantitative analysis of hyphal programme induction in FDR1-carrying wildtype strains after 3 days in hypha-inducing conditions (18 °C, 27 °C 18 °C/WLSE in MM). Note that MC2306, 1E4 and T5 behave similarly to IPO323. Results shown in (**a**, **b**) were obtained independently in 2 (**a**) and 4 (**b**) experiments. Cells used in (**b**, **c**) carried the pCFDR1 plasmid, integrated in their *sdi1* locus; bars in (**c**) represent mean ± SEM, with *n* = 4 biologically independent experiments, each with 20–120 (MC2306), 13–77 (1E4) or 21–81 (T5) structures examined; red dots resemble values from individual experiments; statistical comparisons used Student’s *t*-testing with Welch correction; *significant difference at two-tailed *P* = 0.0243; **significant difference to 12 °C at two-tailed *P* = 0.0059 (**c**, MC2306, 18 °C), 0.0052 (**c**, 1E4, 18 °C) and 0.0069 (**c**, T5, 18 °C), and to 12 °C/MetOH at two-tailed *P* = 0.0026 (**c**, MC2306, 18 °C/WLSE), and between data sets 18 °C and 18 °C/WLSE at two-tailed *P* = 0.0036 (**c**, 1E4); ***significant difference to 12 °C/MetOH at two-tailed *P* = 0.0002 (**c**, 1E4 and T5, 18 °C/WLSE) and between data sets at two-tailed *P* = 0.0005 (**c**, T5); ****significant difference to 12 °C at two-tailed *P* < 0.0001 (**c**). Source data are provided as a Source Data file.

When spores of MC2306, 1E4 and T5 were grown at 27 °C, hyphae formed (Fig. 3a). Such a response was previously shown for strain 1E4^21^. Thus, elevated temperature also cues dimorphic switching in these strains. We introduced FDR1 into the *sdi1* locus of MC2306, 1E4 and T5 and observed cellular fluorescence after growth in MM at 12 °C, 12 °C/MetOH, 18 °C, 27 °C and 18 °C/WLSE. All reporter strains responded to increasing temperature and to WLSE by ZtGFP expression (Fig. 3b, c). Thus, IPO323, MC2306, 1E4 and T5 respond similarly to increased temperatures and leaf surface cues by initiating hyphal growth.

### Dimorphic switch is accompanied by transcriptional changes

**Fig. 4:**
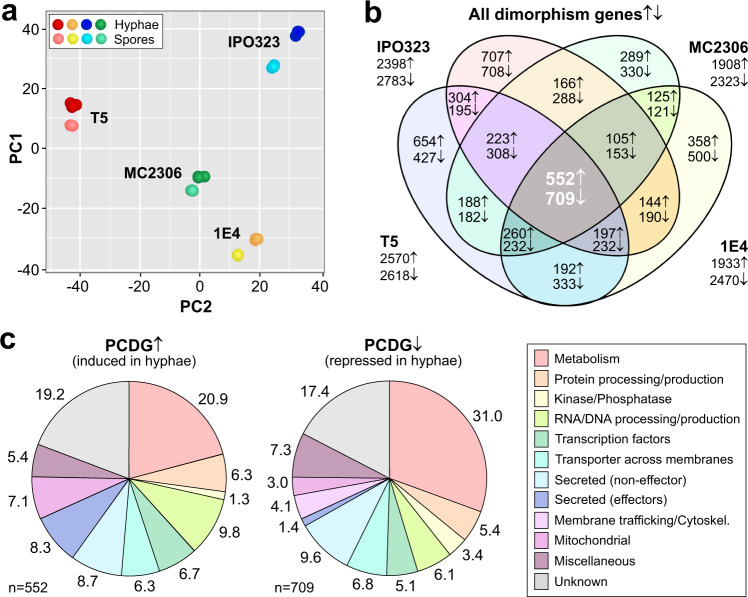
Comparison of gene expression in spores and hyphae of four wildtype strains. **a** Principal component analysis of transcriptomes (indicated by dots) in wildtype strains IPO323, MC2306, 1E4 and T5. Cells were grown for 2–3 days in MM at 18 °C/WLSE (darker symbols) or 12 °C/MetOH (light symbols). Clustering indicates similarity amongst data sets. Number of experiments: 3 (MC2306) or 4 (IPO323, 1E4, T5). Note the diverse transcriptional profiles of the strains. See Supplementary Data 2. **b** Venn diagram showing number of genes, significantly (*P* < 0.01) upregulated (↑) or downregulated (↓) in hyphae of strains IPO323, MC2306, 1E4 and T5, grown in MM at 18 °C/WLSE. Data were obtained by comparison with spores, grown in MM at 12 °C/MetOH. Gene numbers given in sectors. Genes in grey area are considered Pan-Strain Core Dimorphism Genes↑ (PCDG↑) and Pan-Strain Dimorphism Genes↓ (PCDG↓). See Supplementary Data 2 and 4. **c** Functional predictions for Pan-Strain Core Dimorphism Genes↑ (PCDG↑) and Pan-Strain Core Dimorphism Genes↓ (PCDG↓). Note that hyphae induce expression of putative effectors, while spores are characterised by upregulation of metabolic genes. Percentages are given adjacent to sectors. See Supplementary Data 4.

We next set out to identify genes relevant for hyphae formation by comparing the transcriptome of spores, grown at 12 °C/MetOH with that of hyphae, harvested from cultures grown at 18 °C/WLSE, by filtering through Miracloth (see Methods). We firstly asked if the difference in temperature affects the transcriptional activity of *Z. tritici* cells. To address this, we determined the transcriptome of IPO323 cells, grown at 12 and 18 °C (Supplementary Data 1), and checked homologues of 17 nuclear housekeeping genes, described as constitutively expressed in ascomycete fungi (see Supplementary Data 2 and references therein). We found that at 18 °C the average expression of these housekeeping genes is ~20% higher than at 12 °C (1.19-fold higher, 95% confidence interval −1.07- to +1.46-fold; Supplementary Data 2), which likely reflects the increased growth rate at this temperature (see above). To acknowledge this finding, our subsequent comparison of the transcriptomes of 18 °C/WLSE-induced hyphae and 12 °C/MetOH-grown spores only considered genes that showed expression changes beyond this confidence interval. Principal component analysis (PCA) of hyphal transcriptomes of IPO323, MC2306, 1E4 and T5 revealed that all WT strains exhibit distinct expression patterns, both in spores and hyphae, which clearly sets them apart from each other (Fig. 4a). Comparing hyphal and spore gene expression revealed that 4548–5664 genes in each strain are significantly up- or downregulated (Fig. 4b and Supplementary Data 3). This represents 42.7–53.09% of all transcribed genes in each strain (total number at 12 °C/MetOH: IPO323, 11,202; MC2306: 10,645; 1E4: 10,599; T5: 10,505; at 18 °C/WLSE: IPO323, 11,173; MC2306: 10,551; 1E4: 10,702; T5: 10,500). A previous study compared transcriptomes in four WT strains of *Z. tritici* strains and identified candidate genes, expressed in hyphae generated in sugar-free MM^21^. We followed a similar strategy and identified 552 genes that were consistently upregulated and 709 genes that were consistently downregulated in hyphae of IPO323, MC2306, 1E4 and T5 (Fig. 4b, grey area). These two data sets were defined as Pan-strain Core Dimorphism Genes↑ (= PCDG↑) and Pan-strain Core Dimorphism Genes↓ (= PCDG↓; Supplementary Data 4; note that downregulated in hyphae reflects higher regulation in spores).

A significant number of PCDG↑ and PCDG↓ encoded proteins of unknown function (19.2 and 17.4% of all genes, respectively; no domains, no characterised homologues/orthologues; Fig. 4c). On the other hand, ~21% of the PCDG↑ and ~31% of the PCDG↓ were associated with metabolic processes. We searched the PCDG↑ and PCDG↓ core genes for published *Z. tritici* dimorphism factors (Supplementary Table 5). We found the signalling factor MgGpa3^13^, protein kinase subunit MgTpk2^13^ and dimorphism protein ZtMyco#76^36^ (Table 1). We also searched PCDG↑ and PCDG↓ for homologues of fungal transcription factors reported to be involved in development and morphology (Supplementary Table 6). Amongst the 37 transcription factors in PCDG↑ and 36 in PCDG↓, we found homologues of *Botrytis cinerea* (BcATF1), *Neurospora crassa* (Flb3, fluffy and acon-3), *Aspergillus fumigatus* (RgdA) and *Yarrowia lipolytica* (Znc1; Table 1). Finally, we noticed genes encoding light-responsive proteins present amongst the core dimorphism genes, including homologues of the velvet complex^37^, an unknown velvet factor (velvet domain, 2e–05, PFAM) and an *Aspergillus nidulans* velvet-regulated transcription factor NosA^38,39^ (Table 1). This suggested an important role of light in dimorphism in *Z. tritici*. We therefore compared our data sets against published *Z. tritici* genes shown to display >4-fold expression changes in response to light^15^. This revealed that 41.6 and 30.3% of the listed light-dependent genes are present in our PCDG↑ and PCDG↓ data sets, respectively (Supplementary Data 4). This result adds to the growing evidence that light plays a central role in morphogenesis in *Z. tritici*^15–17^.

**Table 1:** Fungal morphology determinants and effectors in PCDG data sets

Name^a^	Gene ID^b^	Homologue^c^	Function	Regulation^d^	*wco*1^e^	Reference
*Proteins involved in light response*						
ZtMve1	ZTRI_2.1013	AnVeA (4e–90)	Velvet factor	2.78↑ (*)	Y	^17^
ZtVelB	ZTRI_7.41	AnVelB (3e–83)	Velvet factor	1.72↓^n^	N	^16^
ZtVosA.1	ZTRI_3.788	AnVosA (8e–44)	Velvet factor	1.68↓	Y	^16^
ZtVosA.2	ZTRI_3.461	AnVosA (2e–22)^f^	Velvet factor	7.94↑	Y	^16^
–	ZTRI_3.864	AnLaeA (9e–52)	Velvet factor	2.88↑	Y	This study
–	ZTRI_3.1090	–	Velvet factor	2.39↓	Y	This study
–	ZTRI_7.76	AnNosA (0.0)	TF^h^	3.98↑	Y	This study
–	ZTRI_1.1139	NcFlbC (4e–59)	TF^i^	5.21↑	Y	This study
Zt_Nop-1	ZTRI_13.368	AnNopA (4e–20)	Opsin^j^	6.67↑	Y	^15^
Zt_Lov1	ZTRI_2.1111	–	Signalling^k,l^	4.23↑	Y	This study
Zt_Lov2	ZTRI_1.2082	–	Unknown^k^	2.33↓	(Y)^o^	This study
Zt_Lov3	ZTRI_4.300	–	Unknown^k^	2.97↑^m^	Y	This study
*Transcription factors participating in development*						
Unknown	ZTRI_1.516	BcATF1 (9e–47)	TF	1.80↑	Y	This study
Unknown	ZTRI_2.479	YlZnc1 (5e–11)	TF	7.90↑	Y	This study
Unknown	ZTRI_4.735	NcFluffy (2e–09)^g^	TF	3.59↓	Y	This study
Unknown	ZTRI_10.332	NcAcr1 (8e–79)	TF	2.42↓	(Y)^p^	This study
Unknown	ZTRI_1.868.1	AfRgdA (1e–143)	TF	1.86↓	Y	This study
Unknown	ZTRI_10.333	SpRrn7 (1e–35)	TF	3.41↓	Y	This study
*Putative effectors*						
Mg1LysM	ZTRI_8.414	–	Effector	8.22↑	Y	^7^
Mg3LysM	ZTRI_11.155	–	Effector	6.34↑	Y	^7^
Mgx1LysM	ZTRI_8.412	–	Effector	11.29↑	Y	^8^
Zt16	ZTRI_9.107	–	Effector	74.35↑	Y	^81^
Zt7	ZTRI_1.1348	–	Effector	13.88↑	Y	^82^
Zt4_1	ZTRI_5.850	–	Effector	25.69↑	Y	^82^
Zt6	ZTRI_11.435	–	Effector	14.95↑	Y	^83^
ZtAvr3D1	ZTRI_7.581		Effector	30.39↑	Y	^44^
Zt26	ZTRI_13.34	–	Effector	3.31↓	N	^83^
Inhibitor i9	ZTRI_10.277	–	Protease inhibitor	2.43↑	N	^41^
Unknown	ZTRI_2.242	CfEcp2 (4e–09)	Effector	3.01↑	Y	This study
Unknown	ZTRI_4.39	VdCP1 (8e–48)	Effector	22.72↑	Y	This study
*Miscellaneous*						
MgGpa3	ZTRI_10.413	–	G protein	1.63↓	(Y)^o^	^13^
MgGpa1	ZTRI_1.856	–	Prot. kinase A	1.42↓	Y	^11^
MgTpk2	ZTRI_3.1002	–	Prot. kinase A	2.01↓	N	^11^
ZtMyco#76	ZTRI_12.391	–	Unknown	2.59↓	Y	^36^

^a^Prefixes are Zt: *Zymoseptoria tritici*, Bc: *Botrytis cinerea*, An: *Aspergillus nidulans*, Af: *Aspergillus fumigatus*; Nc: *Neurospora crassa*, Yl: *Yarrowia lipolytica*, Mg: *Mycosphaerella graminicola* (= Zt, *Zymoseptoria tritici*); Cf: *Cladosporium fulvum*, Co: *Colletotrichum orbiculare*, Vd: *Verticillium dahliae*.

^b^See database FungiDB, https://fungidb.org/fungidb/app/record/dataset/NCBITAXON_336722.

^c^Confirmed by reverse BLASTp search, using NCBI and FungiDB databases; error probability for FungiDB given in brackets.

^d^Average fold-change in hyphae (18 °C/WLSE) compared to spores (12 °C/MetOH) in four wildtype strains; ↑: significantly (*P* < 0.01) induced in hyphae vs. spores; ↓: significantly (*P* < 0.01) repressed in in hyphae vs. spores; for standard deviation and individual values see Supplementary Data 2.

^e^Consistently de-regulated in *wco*1 null mutants of all four wildtype strains.

^f^Weak homology to AnVelC at BLASTp error probability of *P* = 2e–12.

^g^Not highest BLASTp hit.

^h^Controlled by velvet factor AnLeaA^39^.

^i^Controls velvet factor Vos1/VosA^84^.

^j^Green light receptor.

^k^Contains a LOV domain, suggesting blue-light perception.

^l^Contains a regulator of G protein signalling domain (RGS; *P* = 4.2e–08, PFAM).

^m^Consistently up- or downregulated in MC2306, 1E4 and T5, but not in IPO323.

^n^Consistently up- or downregulated in IPO323, 1E4 and T5, but not in MC2306.

^o^Consistently up- or downregulated in MC2306ΔWco1, 1E4ΔWco1 and T5ΔWco1, but not in IPO323ΔWco1.

Fungi perceive light via photoreceptors^40^ and numerous of these proteins were identified in the genome of *Z. tritici*^15^. We searched all PCDG↑ and PCDG↓ for altered expression of photoreceptor genes (Supplementary Fig. 7a; gene IDs in legend). Neither genes encoding homologues of white-collar 1, vivid, phytochrome nor cryptochromes were altered in expression, but we found upregulation of a putative green light-sensing opsin (Zt_Nop-1, ZTRI_13.368; opsin domain, *P* = 2.4e–39, PFAM) with homology to AnNopA, previously shown to be light-regulated^15^ (Table 1, Supplementary Fig. 7a and Supplementary Data 3). In addition, two uncharacterised genes were de-regulated in hyphae (ZTRI_2.1111 in PCDG↑; ZTRI_1.2082 in PCDG↓; these were named Zt_Lov1 and Zt_Lov2, respectively). Both are predicted to carry a light-oxygen-voltage (LOV) domain (Supplementary Fig. 7b). ZT_LOV1 also has an RGS domain (4.2e–08, PFAM), indicating a role in G protein signalling. The genes are distantly related to each other (17.7%/26.7% identity/similarity) and share the highest sequence similarity with uncharacterised genes in other filamentous fungi (Supplementary Fig. 7a). Surprisingly, their primary sequence is also similar to that of blue-light-sensing plant phototropin-2 (e.g. 17.6%/28.0% sequence identity/similarity between Zt_Lov2 and *Arabidopsis thaliana* phototropin-2; Supplementary Fig. 7a). This similarity was highest in the LOV domain (41.3%/65.4% sequence identity/similarity; Supplementary Fig. 7c). This supports the notion that these uncharacterised *Z. tritici* proteins are putative blue-light receptors. Searching the *Z. tritici* genome, we found a third putative LOV domain blue-light-sensing protein (Zt_Lov3, ZTRI_4.300; Supplementary Fig. 7a, b). Expression was upregulated in hyphae of MC2306, 1E4 and T5, but not in IPO323, suggesting it may contribute to hyphal growth in most strains. Taken together, these findings suggest a central role for light in controlling hyphal growth in *Z. tritici*.

### Effectors and PCWDEs are expressed and secreted prior to plant invasion

We noticed that 17% of PCDG↑ and 11% of PCDG↓ entries encode putative secreted proteins (Fig. 4c; SignalP5.0-positive; Supplementary Table 7 lists bioinformatic tools, URLs and references). Amongst these are 28 (PCDG↑) and 34 (PCDG↓) secreted enzymes, including a few putative plant cell wall-degrading enzymes (PCWDEs), such as cellulases and hemicellulases (Supplementary Data 4). In addition, 46 putative secreted effectors were upregulated in hyphae (SignalP5.0- and EffectorP2.0-positive or previously published, Fig. 4c). Of these, 37 have been reported to be upregulated in planta or have been detected in the apoplast (Supplementary Data 4). These effectors included the three LysM proteins Mg1LysM, Mg3LysM and Mgx1LysM^7,8^, the putative protease inhibitor i9^41^ and homologues of Ecp2 from *Cladosporium fulvum*^42^, a SnodProt1-like effector VdCP1 in *Verticillium dahliae*^43^, and the avirulence factor ZtAvr3D1^44^ (see Table 1 for FungiDB gene IDs and BLASTp error probabilities). However, we also found ten putative effector genes downregulated in hyphae (thus are more highly expressed in spores; Supplementary Data 4). The relevance of this observation is unclear.

We compared PCDG↑ with information on protein expression in *Z. tritici* during hyphal colonisation of the leaf apoplast^21,41^. Beside strain 1E4, these studies analysed the in planta transcriptome of three different *Z. tritici* strains. However, they used the same IPO323 reference genome and gene-models as used in our in vitro study, therefore allowing a direct comparison with our data. Thus, we asked if PCDG↑ data are represented in their plant-infection-related data sets. When compared to the first data set^41^, we found 65.2% (=30) of all hyphal-induced effectors, 14 of 28 secreted enzymes and 29 highly induced genes (including the hyphal core opsin gene ZTRI_13.368; Supplementary Data 5, published data sets at 7 and 12 days post infection (dpi) analysed). When compared with the second in planta data set^21^, we found an additional in planta expressed Ecp2-like effector in our PCDG↑ set (ZTRI_2.242). We conclude that axenic hyphae, grown in a plant-free environment, express a large number of infection-relevant proteins, suggesting that hyphal formation primes the fungus for plant invasion.

**Fig. 5:**
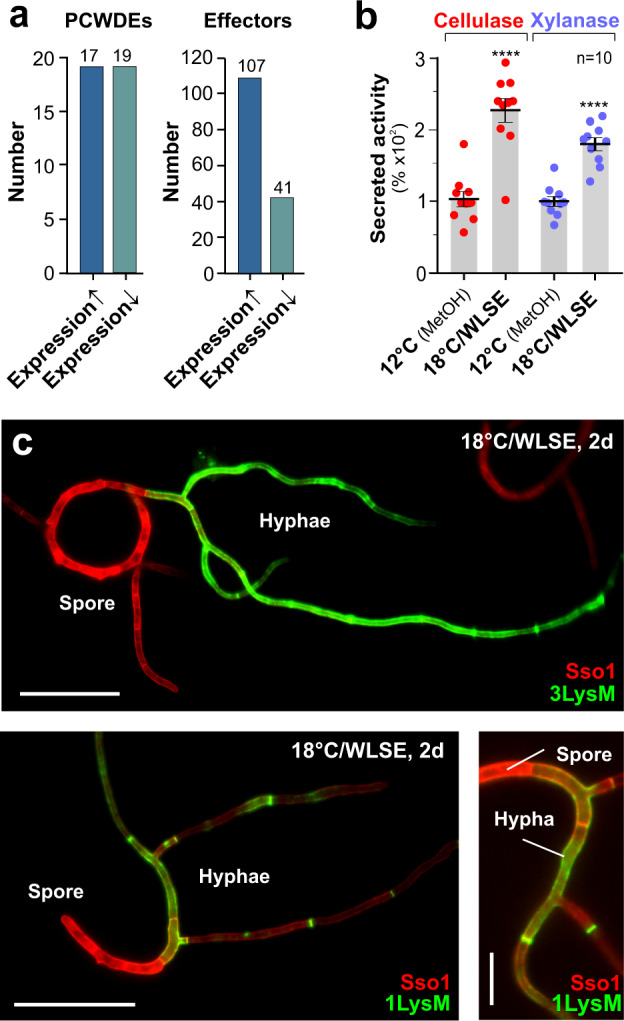
Evidence of PCWDEs and effector secretion in IPO323 hyphae grown in axenic culture. **a** Absolute number of genes, encoding putative plant cell wall-degrading enzymes (PCWDEs) and putative effectors (EffectorP2.0- and Signal P5.0-positive or published) in IPO323 hyphae, grown in MM at 18 °C/WLSE. PCWDEs include putative cellulases, hemicellulases (incl. xylanases), pectinases and cutinases. **b** Activity of cellulases and xylanases in the supernatant of IPO323 cell cultures, grown for 2 days at 12 °C/MetOH or 18 °C/WLSE. **c** Appearance of fluorescent effector 3LysM (green, upper panel; FungiDB gene ID: ZTRI_11.155) and 1LysM (green, lower panels; ZTRI_8.414) in hyphae emerging from spores grown in MM for 2 days at 18 °C/WLSE. The plasma membrane is labelled with fluorescent Sso1^27^ (red). Scale bars = 30 µm (upper and lower left panel), 10 µm (lower right panel). Results shown in (**c**) were obtained independently in 3 experiments. Bars in (**b**) represent mean ± SEM, with *n* = 10 biologically independent experiments; red dots represent individual experiments. Comparison with respective controls in used Student’s *t*-testing with Welch correction; ****significant difference at two-tailed *P* < 0.0001. For image clarity in (**c**) mCherry fluorescence is given in green, and GFP fluorescence in red. All source data for (**b**, **c**) are provided in a Source Data file; for (**a**) see Supplementary Data 1.

Interestingly, hyphae of individual WT strains differ in their numbers of induced PCWDEs and effector genes. IPO323 hyphae, for example, induced 17 PCWDEs, including 11 cellulases and hemicellulases, and 107 effectors (Fig. 5a and Supplementary Data 1), amongst which were some core proteins (Table 1), but also an additional Ecp2 homologue (ZtEcp2.2; ZTRI_13.167; BLASTp error probability *P* = 9e–07) and homologues of necrosis factors Nis1 in *Colletotrichum orbiculare*^45^ (ZtNis1; ZTRI_9.376; *P* = 9e–12) and necrosis factor ZtNip1 in *Z. tritici*^46^ (ZTRI_11.358). To test if IPO323 hyphae secrete PCWDEs, we determined cellulase and xylanase activity in cell-free supernatants of cultures grown at 18 °C/WLSE and 12 °C/MetOH. Indeed, we found a significant increase in enzyme activity under hyphal-inducing conditions (Fig. 5b). We also fluorescently labelled the effectors Mg1LysM and Mg3LysM by fusing their genes to *mCherry* and asked if these effectors are secreted in hyphae, grown in axenic culture at 18 °C/WLSE. Indeed, spores did not express fluorescent effectors, but strong signals were visible at the cell periphery and septa of hyphae (Fig. 5c), which likely represent secreted effectors passing through the cell wall. Thus, we conclude that axenic-grown hyphae of *Z. tritici* produce and most likely secrete effectors, which resonates with previous findings^21,46^.

**Fig. 6:**
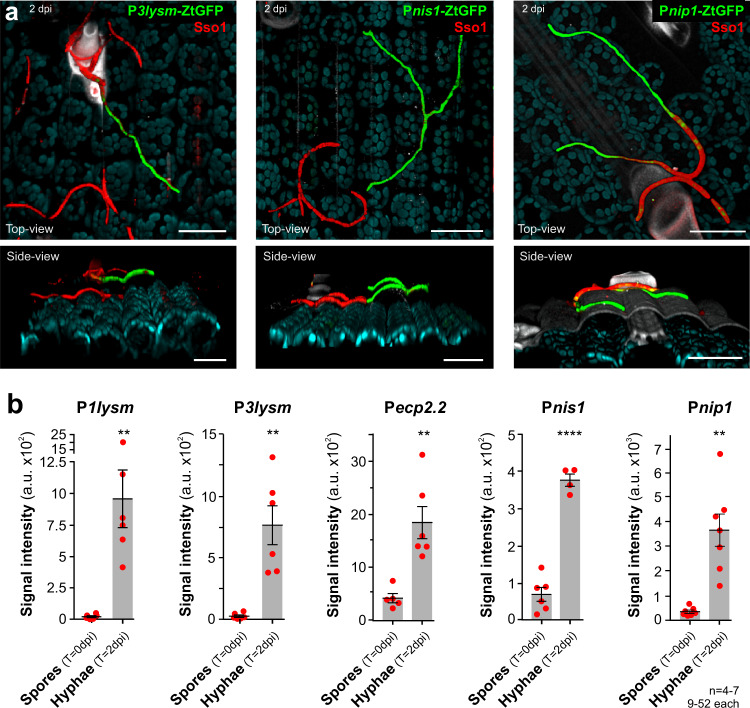
Expression of cytoplasmic GFP under the control of promoters of putative effectors and necrosis-inducing factors in IPO323 in planta. **a** In planta expression of GFP driven by effector *3lysm* promoter (P_*3lysm*_-ZtGFP, green; left panel), the promoter of genes, encoding a homologue of the necrosis-inducing factor Nis1^45^ (P_*nis1*_-ZtGFP, green; middle panel; FungiDB gene ID: ZTRI_9.376) or putative necrosis-inducing factor Nip1^46^ (P_*nip1*_-ZtGFP, green; right panel; ZTRI_11.358). Cells also express fluorescent plasma membrane marker mCherrry-Sso1 (red). Top and side views of image stack reconstructions are shown. Spores show red plasma membrane, whereas switching to hyphae induces GFP fluorescence. Chloroplasts are shown in cyan. White structure in the upper part of the image represents a leaf hair (trichome). Note that the mCherry-Sso1 signal is weak in hyphae. Scale bars = 30 µm. See Supplementary Movies 4–6. **b** Signal intensities of cytoplasmic GFP in spores and hyphae on wheat leaf surface. ZtGFP expression was driven by promoters of effectors Mg1LysM and Mg3LysM^7^, a homologue of effector Ecp2 in *Cladosporium fulvum*^42^ (FungiDB gene ID: ZTRI_13.167), a homologue of the widely conserved necrosis-inducing effector Nis1^45^, and the putative necrosis-inducing factors ZtNip1^46^. In all cases, GFP expression was low in spores but induced significantly upon shift to hyphae. Results shown in (**a**) were obtained independently in 4–7 experiments. Bars in (**b**) represent mean ± SEM, with *n* = 4–7 biologically independent experiments, each with 9–52 structures examined; red dots represent individual experiments. Comparison of data sets used Student’s *t*-testing with Welch correction; **significant difference at two-tailed *P* = 0.0094 (**b**, P*1lysm*), two-tailed *P* = 0.0054 (**b**, P*3lysM*), two-tailed *P* = 0.0050 (**b**, P*ecp2.2*), two-tailed *P* = 0.0025 (**b**, P*nip1*); ****significant difference at *P* < 0.0001 (**b**, P*nis1*). Source data are provided as a Source Data file.

To test if induction of hyphal effector transcription occurs under natural conditions on the plant surface, we generated reporter IPO323 strains, carrying ZtGFP controlled by 1000 bp promoters of effectors Mg1LysM, Mg3LysM, ZtEcp2.2 and putative necrosis factors ZtNis1 and ZtNip1. Two days post inoculation of leaves, low or negligible expression of cytoplasmic ZtGFP was found in spores (Fig. 6a, plasma membrane visualised by mCherry-Sso1 expression and Fig. 6b). However, all promoters were induced upon switching to hyphae, resulting in significantly increased cellular green fluorescence (Fig. 6a, b and Supplementary Movies 4–6). Thus, we conclude that the expression of effectors, including necrosis factors, is induced upon spore-to-hypha transition on the wheat leaf surface prior to plant invasion.

### The white-collar complex controls hyphal formation

**Fig. 7:**
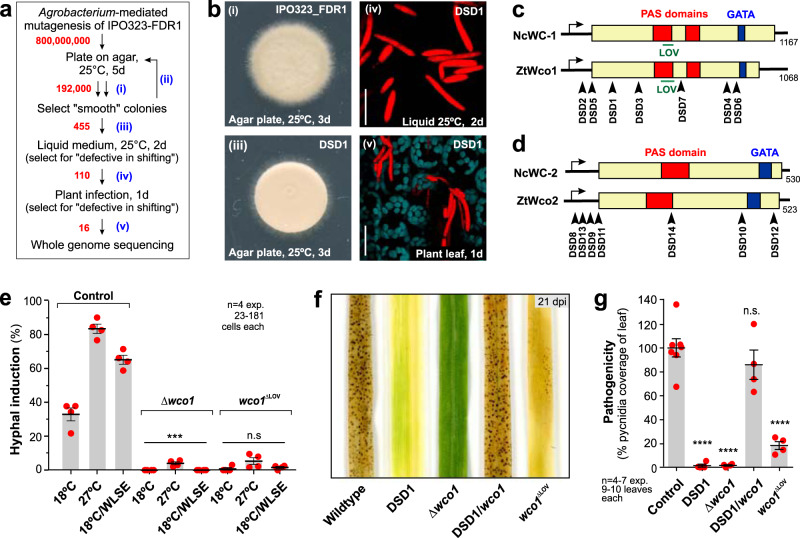
A genetic screen reveals the light-sensing white-collar complex (WCC) as a regulator of dimorphism and pathogenicity. **a** Strategy underlying the genetic screen for mutants defective in dimorphic switching. Mutant numbers in red, experimental steps in blue. See Supplementary Fig. 8. **b** Experimental steps in screen (see **a**, blue numbers). Spores in the right panels express only mCherry (red), indicating the absence of dimorphic switching. Scale bars = 15 µm (upper right panel) and 20 µm (lower right panel). **c**, **d** Domain structure of *Z. tritici* Wco1 and Wco2 and predicted mutations in DSD1-DSD7 (**c**) and DSD8–DSD14 (**d**). NcWC1: *N. crassa* white-collar 1, NcWC2: *N. crassa* white-collar 2. Note that Wco1-encoding gene ZTRI_11.64 does not carry the 405 bp deletion, previously reported for another IPO323 isolate^10^. **e** Hyphal programme induction in Δ*wco*1 and *wco*1^ΔLOV^ mutants. Neither mutant is induced by temperature and/or leaf cues. See Supplementary Figs. 9 and 10. **f**, **g** Disease symptoms (pycnidia) on leaves at 21 days after infection with IPO323 (Control), mutant DSD1, *wco*1 deletion mutant (Δ*wco*1), DSD1 complemented with a *wco*1 wildtype allele (DSD1/*wco*1), and light-blind *wco*1^ΔLOV^ allele (ΔLOV). For other DSD mutants see Supplementary Fig. 11. Results shown in (**b**) were obtained independently in 2 experiments. Cells used in (**a**, **b**, **e**) carried the pCFDR1 plasmid; cells were grown for 2 days in MM under conditions indicated (**e**); bars in (**e**, **g**) represent mean ± SEM, with *n* = 4 (**e**) or *n* = 4–7 (**g**) biologically independent experiments, each with 23–181 structures (**e**) or 9–10 leaves (**g**) examined; with sample size *n* given in graph; red dots represent individual experiments; statistical comparisons used one-way ANOVA (**e**) and Student’s *t*-testing with Welch correction (**g**); n.s. = not statistically different at two-tailed *P* = 0.0699 (**e**), or at two-tailed *P* = 0.3711 (**g**); ***statistically different at two-tailed *P* = 0.0004 (**e**); ****statistically different to Control at two-tailed *P* < 0.0001 (**g**). Source data are provided as a Source Data file.

To obtain insight into the regulation of the spore-to-hypha transition, we performed an *Agrobacterium*-mediated FDR1-based genetic screen for dimorphic switching defective (DSD) mutants. We randomly integrated a G418 resistance cassette into ~800 million IPO323_FDR1 cells, giving 192,000 G418-resistant transformants (Fig. 7a). This number of insertions likely saturates all genes in the genome (*P* = 0.9996), as calculated from the genome size (39,686 kb, average gene length is 1.608 kb; Supplementary Fig. 8). We selected 455 transformants that formed smooth colonies on agar plates (Fig. 7a, b). We tested 110 of these on the leaf surface for dimorphic switching (Fig. 7a, b), which led to the identification of 16 DSD mutants, impaired in spore-to-hypha transition, both in vitro and in planta.

We performed whole-genome sequencing of the 16 DSD mutants and identified 7 mutants (DSD1–7), where the G418 resistance cassette had been inserted into a gene with sequence homology to the blue-light receptor/transcription factor white-collar 1 in *Neurospora crassa* (WC-1^47^). The predicted *Z. tritici* gene product, named hereafter ZtWco1 (ZTRI_11.64), displays 37.0%/48.7% sequence identity/similarity and a similar domain structure (Fig. 7c, G418-cassette insertion sites in DSD mutants indicated; PAS9, *P* = 6.4e–17; PAS3, *P* = 1.0e–10; GATA, *P* = 5.6e–14; all PFAM32.0 predictions). ZtWco1 also contains a LOV domain with 11 flavin-contacting residues, required for blue-light sensing^48^ (Supplementary Fig. 9a). Seven other DSD mutants (DSD8–DSD14) showed insertions into a homologue of WC-2, the *N. crassa* white-collar 2 protein (ZTRI_1.350; Fig. 7d, G418-cassette insertion sites in DSD mutants indicated; PAS3, *P* = 5.8e–11, GATA, *P* = 1.4e–15). The two remaining DSD mutants were defective in hypothetical proteins, either without detectable domains (DSD15; ZTRI_9.470; located on chromosome 9), or with an N-terminal Enoyl-CoA hydratase domain (*P* = 1.5e–21) and a C-terminal BTB/POZ domain (*P* = 1.0–16; DSD16; ZTRI_3.375; located on chromosome 3). To confirm the role of white-collar 1 in dimorphism in *Z. tritici*, we deleted *wco*1 in IPO323_FDR1. The resulting *wco*1 null mutant formed spores at 12 °C but did not switch to hyphal growth at 18 °C, 27 °C or 18 °C/WLSE (Fig. 7e and Supplementary Fig. 10a) or on leaves (Supplementary Fig. 10b). In summary, our genetic screen revealed 14 of 16 mutants defective in either *wco*1 or *wco2*. As *N. crassa* WC-1 and WC-2 form the WCC^49^, we conclude that the WCC is crucial for dimorphic switching in *Z. tritici*.

*N. crassa* WC-1 detects blue light via a LOV sensing domain; its deletion results in loss of light-sensing^50^. We generated an analogous light-blind allele in *wco*1 by deleting residues 420–534 in strain IPO323_FDR1. We tested if the cells of strain IPO323_FDR1_Wco1^ΔLOV^ lose their ability to sense light, by growing this mutant and its mother strain IPO323_FDR1 for 2 days in the dark. Under this condition, IPO323_FDR1 formed green-fluorescent hyphae, whereas IPO323_FDR1_Wco1^ΔLOV^ did not show dimorphic switching (Supplementary Fig. 9b, c). Thus, we conclude that ZtWco1^ΔLOV^ had lost its ability to detect light. Next, we tested if the light-blind ZtWco1^ΔLOV^ protein supports the spore-to-hypha transition. We found that neither 18 or 27 °C, nor 18 °C/WLSE induced dimorphic switching of strain IPO323_FDR1_Wco1^ΔLOV^ (Fig. 7e; Wco1^ΔLOV^). This suggests that WCC control of dimorphic switching requires light perception.

We tested if the WCC is required for pathogenicity in *Z. tritici*. Wheat leaves were infected with IPO323, DSD1, a DSD1 mutant complemented with WT *wco*1 allele, the *wco*1 null and the light-blind *wco*1^ΔLOV^ mutant. At 21 days post inoculation, we assessed disease development by quantifying numbers of pycnidia. We found that WT and complemented DSD1 mutant strains formed melanised pycnidia (Fig. 7f, g; Control and DSD1/*wco*1), whereas *wco*1 mutant strains were strongly impaired in pycnidia formation (Fig. 7f, g; DSD1, Δ*wco*1, *wco*1^ΔLOV^). The remaining DSD mutants, mutated in *wco*1 or *wco2*, developed only weak disease symptoms in infected plants (Supplementary Fig. 11). Thus, we conclude that the WCC complex is crucial for wheat infection by *Z. tritici*, likely due to its pivotal role in dimorphic switching.

### White-collar controls the expression of most dimorphism core genes

**Fig. 8:**
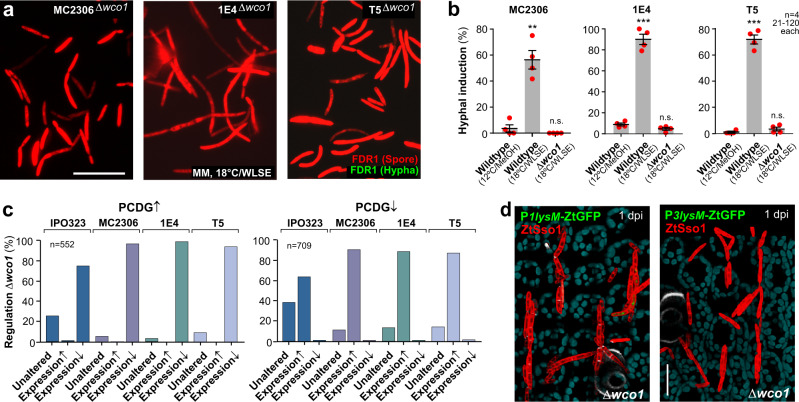
Gene expression in *wco*1 null mutants, grown in hyphal-inducing conditions, in the genetic background of four wildtype strains. **a** Images of FDR1-encoded fluorescent protein expression in Δ*wco*1 mutants in the genetic background of MC2306, 1E4 and T5 after 3 days at 18 °C/WLSE. Spores only induce expression of mCherry (red), indicating the absence of dimorphic switching and confirming a pivotal role of WCC is pivotal to dimorphism of *Z. tritici*. Scale bar = 20 µm. **b** Quantitative analysis of hyphal programme induction in FDR1-carrying Δ*wco*1 mutants in the genetic background of MC2306, 1E4 and T5 after 3 days at 18 °C/WLSE. For comparison, data for wildtype (12 °C/MetOH and 18 °C/WLSE) were taken from Fig. 3c). **c** Effect of *wco*1 deletion on expression of Pan-strain Core Dimorphism Genes↑ (PCDG↑) and Pan-strain Core Dimorphism Genes↓ (PCDG↓). Gene expression in mutants in the IPO323_ΔWco1, MC2306_ΔWco1, 1E4_ΔWco1 and T5_ΔWco1, grown at 18 °C/WLSE, was compared to their respective wildtype progenitor. Note that less IPO323 genes are under the control of Wco1. See Supplementary Data 4. **d** In planta expression of GFP under the *1lysm* promoter (P*1lysm*-ZtGFP, green) and *3lysm* promoter (P*3lysm*-ZtGFP, green) in Δ*wco*1 mutants. Cells also express the fluorescent plasma membrane marker mCherrry-Sso1 (red); chloroplasts are shown in cyan, root hairs appear white. In agreement with results from liquid culture experiments, Δ*wco*1 mutants faintly express the reporter GFP. Scale bars = 20 µm. Results shown in (**a**, **d**) were obtained independently in 2 experiments. Cells used in (**a**, **b**) carried the pCFDR1 plasmid, integrated in their *sdi1* locus; bars in (**b**) represent mean ± SEM, with *n* = 4 biologically independent experiments, each with 21–120 structures examined; with sample size *n* given in graph; red dots resemble values from individual experiments; statistical comparisons used Student’s *t*-testing with Welch correction; n.s. = non-significant difference to 12 °C/MetOH at two-tailed *P* = 0.2821 (**b**, MC2306_Δwco1), 0.0633 (**b**, 1E4_Δwco1), and 0.3012 (**b**, T5_Δwco1); **significant difference to 12 °C at two-tailed *P* = 0.0026 (**b**, MC2306, 18 °C/WLSE); ***significant difference to 12 °C/MetOH at two-tailed *P* = 0.0002 (**b**, 1E4 and T5, 18 °C/WLSE). Source data are provided as a Source Data file.

We deleted *wco*1 in MC2306, 1E4 and T5 strains carrying FDR1. When grown under hypha-inducing conditions (18 °C/WLSE), none of these mutants expressed ZtGFP (Fig. 8a, b), confirming the importance of WCC in initiating the hyphal programme in *Z. tritici*. We deleted the white-collar 1 gene in the WT strains, resulting in MC2306_ΔWco1, 1E4_ΔWco1, T5_ΔWco1 and IPO323_ΔWco1 and determined gene expression in cells, grown at 18 °C/WLSE, followed by comparing their transcriptome to their respective WT hyphae. In the absence of *wco*1 between 3953 and 5935 genes were up- or downregulated in the four strains, thus representing a third and half of all predicted 11,841 genes. We next considered the importance of Wco1 for the regulation of PCDG↑ and PCDG↓ candidate genes and compared their expression in WT and Δ*wco*1 mutants. This revealed that ~74% (IPO323) and ~92–98% (MC2306, 1E4, T5) of all upregulated genes are reduced in expression when *wco*1 is absent (Fig. 8c, PCDG↑; Supplementary Data 4), whilst ~62% (IPO323) and ~86–88% (MC2306, 1E4, T5) of all downregulated core genes are now significantly upregulated (Fig. 8c, PCDG↓; Supplementary Data 4). This suggests that WCC controls the expression of most genes associated with dimorphic switching. As the WCC promoter-binding motif is well characterised^51^, we searched 1000 bp upstream of the open reading frames of all PCDGs for this motif and found 208 significant hits (*P* < 1e–4). Finally, we generated *wco*1 null mutants in P*1lysm*-ZtG and P*3lysm*-ZtG reporter strains and assayed their in planta expression. Consistent with the transcriptome results, no expression of ZtGFP was observed in these null mutants (Fig. 8d). Collectively, our data demonstrate a central role for WCC in induction of dimorphism-associated factors.

### Light conditions determine successful wheat infection by *Z. tritici*

**Fig. 9:**
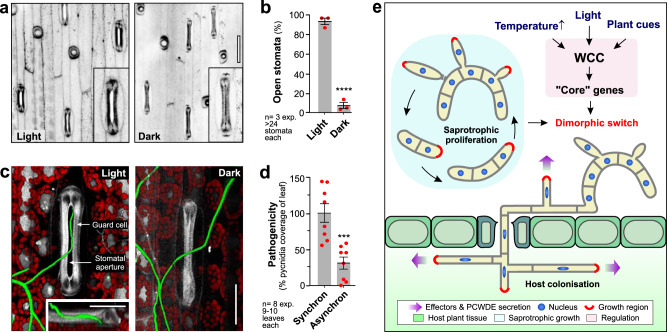
Effect of altered light phase on infection. **a** Contrast inverted images of the upper leaf epidermis in light or dark. Insets show enlarged stomata. Scale bar = 50 µm. **b** Percentage of open stomata in light and dark. **c** *Z. tritici* hyphae, expressing cytoplasmic GFP (green), on wheat leaves, grown in light or dark. Hyphae enter open stomata (Light) but pass over closed stomata (dark). A guard cell and the stomatal aperture are indicated. Chloroplasts are shown in red. Scale bars = 3 µm in side-view insert; 30 µm in overviews. **d** Disease symptoms in infection assays under different light conditions. Synchron = host and pathogen grown under the same 12 h light/12 h darkness regime; Asynchron = host and pathogen grown 12 h out of phase. **e** Model of the role of WCC in controlling dimorphic switching and pathogenicity in *Z. tritici*. During saprotrophic growth in plant debris, *Z. tritici* proliferates cell numbers by forming multi-cellular blastospores by lateral budding. On wheat leaves, the white-collar complex (WCC) senses light and integrates this information with unknown cues on the wheat leaf surface, as well as elevated temperatures. This induces dimorphic switching, most likely driven by changes in the expression of core dimorphism genes, which are largely controlled by WCC. Hyphae secrete effectors and PCWDEs and invade through stomata to colonise the plant. Results shown in (**a**, **c**) were obtained independently in 2 (**c**) and 3 (**a**) experiments. Bars in (**b**, **d**) represent mean ± SEM, with *n* = 3 (**b**) or *n* = 8 (**d**) biologically independent experiments, each with >24 stomata (**b**) or 9–10 leaves (**d**) examined; with sample size *n* given in graph; red dots represent individual experiments; statistical comparisons used Student’s *t*-testing with Welch correction; ***statistically different at two-tailed *P* = 0.0008 (**d**); ****statistically different to Control at two-tailed *P* < 0.0001 (**b**). Source data are provided as a Source Data file.

We show here that Wco1 is a master regulator of dimorphic switching and that its ability to sense light via its LOV domain is pivotal to this role. Successful infection by *Z. tritici* requires entry of hyphae through open stomata. As stomatal opening itself occurs in response to blue light^52^, we speculated that blue-light-sensing of ZtWco1 synchronises hyphae formation with stomatal opening for successful infection. We confirmed that stomata are open when exposed to light, but closed in the dark (Fig. 9a, b). To evaluate the role of light in infection, we grew wheat and *Z. tritici* under two different light regimes: (i) synchronous conditions, with pathogen and host kept in the same light/dark cycle (12 h light, 12 h darkness) and (ii) asynchronous conditions, where pathogen and host were in 12 h light/dark cycles that were shifted by 12 h. Synchronous and asynchronous growth conditions were maintained for >10 days, prior to inoculation at the beginning of their dark period. In the synchronous situation, GFP-expressing IPO323 cells entered through open stomatal apertures (Fig. 9c, light), whereas hyphae passed over closed stomata in asynchronous infections (Fig. 9c, dark). Consequently, asynchronous infection assays resulted in a significant reduction of disease symptoms (Fig. 9d). These results support the notion that WCC integrates light, temperature and leaf surface cues to optimise invasion through stomata (Fig. 9e).

## Discussion

In this study, we consider the cues and molecular processes driving dimorphism in the wheat pathogen *Z. tritici*. The transition from a spore to a hypha marks the onset of pathogenicity, enabling the fungus to invade host leaves via open stomata^6^. The infection cycle is completed by the formation of melanised pycnidia, which release their asexual spores to fuel subsequent repeated cycles of infection during a given wheat-growing season^4^.

Experimental studies have shown that dimorphic switching in *Z. tritici* can be induced by the transfer of spores from 18 to 25–28 °C^13,17,20^. However, others consider that these high temperatures cause heat stress in the fungal cell^21–24^. We confirm here that such elevated temperatures induce promoters of heat stress chaperones, suggesting heat stress to the cells. This response may, however, differ amongst *Z. tritici* strains, as a range of temperature optima have been reported^53^. Our climate data analysis shows such elevated temperatures do occur in wheat fields during summer months. Thus, high temperature alone may not be the major driver of hyphal formation in *Z. tritici*.

Dimorphic switching is generally assessed by changes in cell morphology. We developed a fluorescent reporter system (FDR1), which differentiates spores expressing red-fluorescent protein from hyphae expressing green-fluorescent protein. This reporter enabled us to investigate the early induction of the hyphal programme before morphological changes become apparent. Using this FDR1 system, we show that dimorphic switching occurs in approximately 1/3 of all spores in 4 tested WT strains even at 18 °C. Our climate data reveal that this temperature occurs frequently in wheat fields. However, the low number of dimorphic switching suggests additional cues that trigger dimorphic switching prior to infection.

Successful invasion of wheat plants occurs when *Z. tritici* spores, located on the leaf, undergo dimorphic switching. It is thought that the fungus faces carbon limitations on the leaf surface and that this could be a cue for dimorphic switching^21^. However, sucrose is found on plant leaves^54^, and our GC/MS analysis of wheat leaf washings confirms the presence of sucrose. Consequently, carbon-limitation may not be a major trigger for hyphal growth, thus explaining why IPO323_FDR1 cells do not switch after 2 days in carbon-limiting conditions. While previous reports demonstrate hyphal growth induction in the absence of sucrose as a carbon source, this work was performed in Vogel’s medium^21^. Here, the presence of 2.5% sodium citrate in this medium could provide an alternative carbon source for fungal growth^55^, which makes interpretation of these results problematic. We also report that nitrogen-depletion does not induce dimorphic switching within 2 days. While a study reported hyphal growth after 72 h in nitrogen-depleted medium^21^, such late appearance of hyphae does not reflect the situation on the leaf surface, where hyphae appear after 1 day^5,32^. Indeed, we show here that the first GFP expression from FDR1 occurs around 1 h after spores were inoculated onto the wheat leaf surface. This rapid response supports the notion that starvation stress is not the primary cue for yeast-to-hypha transition at the onset of pathogenic development.

Spores rest on the wheat leaf cuticle for hours before forming hyphae^32^. In planta, IPO323_FDR1 shows rapid dimorphic switching, suggesting that fungal cells perceive unknown leaf surface compounds, such as cuticular waxes or surface sugars. Cuticular waxes are known to induce yeast-to-hypha transition in the maize pathogen *Ustilago maydis*^56^, and germination in the grey mould *Botrytis cinerea*^57^, the powdery mildew fungus *Blumeria graminis* and the rice blast fungus *Magnaporthe oryzae*^58^. This attests to a putative role for plant surface cues in initiating developmental change in *Z. tritici*. We treated spores with WLSE, prepared with chloroform-washed leaves. WLSE alone initiated dimorphic switching of spores at 12 °C, but in combination with 18 °C significantly increased hyphae formation in all four WT strains. GC/MS revealed the presence of several alkanes and long chain fatty alcohols, which are known epicuticular components^59^, as well as glycerol and sucrose. Intracellular plant metabolites, such as organic acids and amino acids, commonly detected by GC/MS in plant cell extracts, were absent^60^. Thus, our WLSE likely contains compounds that provide plant surface cue(s) for dimorphic switching. While further research is needed to identify these compounds, the combination of a moderate temperature and molecules from wheat leaf surfaces appear highly relevant for orchestrating infection under field conditions.

We report that the spore-to-hypha transition is accompanied by global transcriptional changes. By comparing our PCDG↑ data set, obtained from 2-day-old hyphae that were incubated with wheat leaf surface compounds at 18 °C, with transcriptome data from *Z. tritici*-infected leaves^21,41^, we confirm that numerous infection-related effectors are, indeed, induced in axenic hyphae. This observation supports previous findings in strain 1E4 that show induction of 44 effector genes, including the avirulence factor ZtAvr3D1^21,44^. Based on these findings, it was suggested that morphogenesis and virulence genes are co-regulated^21^. Our results confirm this notion, but also show that this occurs on the plant surface. Thus, it appears that the pathogen arms itself prior to the invasion of the plant tissue.

Amongst the effectors in our PCDG↑ data set are three LysM effectors, shown to protect the fungus against plant chitinases^7,8^. These proteins are well-established virulence factors in pathogenic fungi^61^, and we show that they are expressed and secreted in hyphae grown in axenic culture. This, again, suggests that axenic hyphae represent the infectious plant-colonising morphotype. However, we also recorded differences between the transcriptional responses of the four WT strains. In particular, hyphal effector production could be of relevance for strain-specific variations in the pattern and timing of wheat infection, as reported previously^35^. Indeed, each WT strains induced 89–119 effector genes, yet only 47 are commonly upregulated in all strains. While effector upregulation appears to occur prior to infection, the importance of this phenomenon for wheat infections remains elusive.

Fungi sense and respond to environmental light^40^, and various studies have shown that light-dependent factors are involved in morphogenesis in *Z. tritici*^16,17^. Our transcriptome analysis provided additional evidence for the role of light in controlling hyphal growth in *Z. tritici*. Most importantly, we found that ~1/3 of all PCDGs represent light-regulated genes, previously reported in *Z. tritici*^15^. In addition, we found consistent upregulation of an opsin, and uncharacterised LOV domain-containing proteins with overall sequence similarity to uncharacterised proteins in other fungi and *A. thaliana* phototropin-2. Opsins are involved in green light sensing in fungi^40^, but the role of these uncharacterised LOV proteins remains unknown. We also found five putative velvet factors regulated in hyphae in a consistent manner across all four WT strains. The velvet complex coordinates light information with fungal development^37^, again highlighting the importance of light for hyphal growth in *Z. tritici*. Amongst the PCDGs is velvet factor Mve1^17^, required for hyphal growth in *Z. tritici* and *N. crassa*^17,62^. This resonates with its upregulation in hyphae. A second velvet factor, ZtVelB, reported to be required for spore formation^16^, is downregulated in hyphae of 3/4 WT strains. Finally, we found an uncharacterised gene (ZTRI_3.1090), which has no homologue in *A. nidulans*, but carries a velvet domain. Understanding the role of this putative velvet protein in hyphal growth poses a future challenge.

We show that WCC regulates ~62–98% of all core hyphal genes in the four WT strains. About 16–17% of these genes carry a WC-1 promoter-binding motif, suggesting that WCC may directly control a subset of core genes. The majority, however, is probably indirectly controlled via downstream TFs. Such WCC-dependent regulatory network was previously suggested in *N. crassa*^51^. Amongst the WCC-controlled TFs are regulators previously shown to be involved in developmental changes in fungi. These include homologues of *N. crassa* transcription factors fluffy and ACON-3, which control the transition from hyphal to budding growth at the onset of conidiation^63^; hyphal upregulation of a homologue of *Yarrowia lipolytica* Znc1p^64^, which represses hyphal growth in this dimorphic yeast and a homologue of *Aspergillus fumigatus* AsRgdA^65^, required for hyphal growth, which is downregulated in *Z. tritici* hyphae. Thus, our data show that important regulators of fungal development change their expression in a WCC-dependent manner. However, their precise role in dimorphic switching in *Z. tritici* remains elusive.

Our work identifies the WCC as a master regulator for the initiation of hyphal growth in *Z. tritici*. Both our DSD mutants and *wco*1 null mutants failed to form hyphae and, consequently, were non-pathogenic. The role of *wco*1 homologues in fungal virulence has been studied in other fungi, yet its role in pathogenicity is inconsistent. In *Botrytis cinerea*, deletion of *wc1* reduced disease symptoms in *A. thaliana* and French bean by ~20–50%^66^, whereas deletion of *wc-1* in *Magnaporthe oryzae* and *Fusarium oxysporum* had little effect on disease progression^67,68^. An exception to this is seen in the leaf spot fungus *Cercospora zeae-maydis*, which is closely related to *Z. tritici* (family *Mycosphaerellaceae*). Akin to *Z. tritici*, this maize pathogen invades its host via stomata^69^. However, while *Z. tritici* enters open stomata at random and without further morphological differentiation^4,5^, *C. zea-maydis* is chemically attracted to stomata and invades following appressorium formation^70^. Such stomatal tropism and appressorium formation in *C. zea-maydis* depends on light and on white-collar 1-homologue CRP1^69^, yet hyphal growth is unaffected in *crp1* mutants. Thus, while white-collar 1 is crucial for pathogenicity in both Dothideomycete fungi, its role in infection differs significantly between these two pathogens.

The question remains as to why WCC plays such a central role at the onset of pathogenic development in *Z. tritici*. Some fungal pathogens force entry through closed stomata, whereas others require open stomata for invasion^71^. Prior to infection, *C. zea-maydis* forms appressorial infection structures which overlie stomata. As increased turgor pressure within appressoria physically forces ingress into the host^72^ this suggests that *C. zea-maydis* breaches closed stomata. *Z. tritici* does not form infection structures and thus likely requires open stomata for plant invasion. Stomatal opening is controlled by phototropins, which sense blue light via their LOV domain^52^. As the LOV domain in Wco1 recognises the same light spectrum, we suggest that white-collar 1 coordinates and synchronises hyphal growth with stomatal opening, thereby increasing the likelihood and thus the efficiency of infection. Indeed, we show that when the plant and pathogen are exposed to asynchronous light conditions the infection success and pathogenicity of *Z. tritici* is significantly reduced. Thus, we report here that the WCC plays a crucial role in *Z. tritici* pathogenesis, as it integrates light perception with temperature and cues from the plant surface to control the expression of core dimorphism genes that orchestrate host invasion.

## Methods

### Biological material

#### Bacteria

Plasmids were propagated in *Escherichia coli* strain DH5α (Thermo Fisher Scientific, UK). *A. tumefaciens*-mediated transformation of *Z. tritici* used strain EHA105 (GoldBio, St Louis, USA). Bacteria were grown in double-strength yeast extract/tryptone DYT medium (tryptone, 16 g l^−1^; yeast extract, 10 g l^−1^; NaCl, 5 g l^−1^; with 20 g l^−1^ agar in plates) at 37 and 28 °C, respectively.

#### Fungi

*Z. tritici* strains were generated in WT strain IPO323 (Centraalbureau voor Schimmelcultures, The Netherlands; cbs 115943), MC2306 (collected in South England by Dr Helen Fones, Exeter University), 1E4; collected in Switzerland^34^; courtesy of Prof. Bruce McDonald, ETH Zürich) and T5 (collected in North America; courtesy of Prof. Gert Kema, Wageningen University). *Z. tritici* strains, inoculated from glycerol stocks (nutrient broth 8 g l^−1^; sucrose, 5 g l^−1^; yeast extract, 1 g l^−1^; glycerol, 700 ml l^−1^) stored at −80 °C, were grown on Yeast Peptone Dextrose (YPD) agar (yeast extract, 10 g l^−1^; peptone, 20 g l^−1^; glucose, 20 g l^−1^; agar, 20 g l^−1^) for 4–5 days at 12, 15 or 18 °C. For genotypes of strains and plasmid descriptions, as well as experimental usage of all fungal strains see Supplementary Tables 2–4.

#### Plants

Plant-infection assays were with winter wheat (*Triticum aestivum)* cultivar Galaxie (Fenaco Cooperative, Bern, Switzerland) or cultivar Consort (RAGT, Cambridge, UK).

### Field wheat growth temperature analysis

Estimates of canopy temperatures in Kelvin for May, June and July for the last 30 years, taken at 3-h intervals and 0.56° × 0.56° spatial resolution (~60 × 40 km), were downloaded from JRA-55^31^ via Research Data Archive (https://rda.ucar.edu/datasets/ds628.0/ for Southern England (2.0oE–1.4oW, 51.1oN–52.8oN) and North Eastern France (0.8oW–7.0oW, 47.2oN–51.1oN) and converted to Celsius. Information on wheat-growing areas was obtained from https://ipad.fas.usda.gov. The 3-hourly resolution geospatial temporal climate data analysis was performed in R version 4.0.1 (custom R code available at https://github.com/thomaschaloner/Zymoseptoria-tritici-white-collar-complex-integrates-light-temperature-and-plant-cues).

### Induction of the dimorphic switch from spore to hypha in vitro

One litre MM consists of 50 ml l^−1^ of salt solution (NaNO_3_, 120 g l^−1^; KCl, 10.4 g l^−1^; MgSO_4·_7H_2_O, 10.4 g l^−1^; KH_2_PO_4_, 30.4 g l^−1^), 1 ml l^−1^ trace elements (ZnSO_4_·7H_2_O, 2.2 g l^−1^; H_3_BO_3_, 1.1 g l^−1^; MnCl_2_·4H_2_O, 0.5 g l^−1^; FeSO_4_·7H_2_O, 0.5 g l^−1^; CoCl_2_·5H_2_O, 0.16 g l^−1^; CuSO_4_·5H_2_O, 0.16 g l^−1^; (NH_4_)_6_Mo_7_O_24_·4H_2_O, 0.11 g l^−1^; Na_4_EDTA, 5 g l^−1^), glucose, 10 g l^−1^. Carbon-free MM does not contain glucose, nitrogen-free MM does not contain NaNO_3_. 10 ml of this MM was inoculated with ~5 million spores, obtained from YPD agar plates, and grown for 2 days in MM or MM/0.5% (v v^−1^) MetOH at 12 °C at 200 rpm, using an Innova 44 shaker (New Brunswick Scientific). Hyphae were induced by incubation in MM for 2–3 days, 200 rpm, at 18 or 27 °C, or at 18 °C in MM plus WLSE (500 µl WLSE stock solution per 100 ml of MM). WLSE stock solution was prepared by cutting and immersing 30 wheat leaves (12–14 days old and ~10 cm long; second leaf) in 15 ml of chloroform, ensuring that cut tissues did not contact the chloroform. After 3 min the solution was poured into a glass petri dish, and the solvent was left to evaporate overnight. The residue was re-suspended in methanol (final volume 2 ml = WLSE stock solution).

### Determining the cellular doubling time of *Z. tritici* at 12 and 18 °C

Pre-cultures of strain IPO323_eGSso1, which allows detection of individual cells in the multi-cellular spores, were inoculated in 10 ml YG medium (yeast extract, 10 g l^−1^; glucose, 30 g l^−1^) and grown for 3 days at 12 °C, 200 rpm. Spore numbers were counted using a Cellometer Auto 1000 cell counter (Nexcelom Biosciences, Lawrence, USA) and adjusted to ~5.5 × 10^5 ^ml^−1^, using a fresh YG medium. 10 ml of these cultures were incubated for up to 73 h at 12 and 18 °C and the number of spores was counted at various time points using the Cellometer Auto 1000 cell counter. At each sampling, average cell numbers were determined microscopically by observing eGFP-Sso1 and the counted spore numbers were corrected for these values. The obtained cell numbers were plotted and the doubling time was determined using GraphPad Prism 9.

### GC/MS analysis

Methoximated trimethylsilyl derivatives were prepared and analysed by accurate mass GC/MS using a GC-EI-qToF MS instrument (Agilent 7200, Agilent Technologies, Santa Clara, CA, USA). The WLSE and control samples (900 μl) were sequentially dried into 0.2 ml tapered glass vials. Then, 20 µl of 20 mg ml^–1^ methoxyamine hydrochloride, dissolved in pyridine, were added to each sample and incubated at 37 °C for 2 h, thence 35 µl of N-methyl-N-(trimethylsilyl)trifluoroacetamide added and incubated for 30 min. Vials were sealed with polypropylene septa and placed in the GC autosampler. Derivatives were injected (0.6 μl, 12:1 split ratio) onto a Zebron SemiVolatiles GC column (30 m analytical + 10 m guard length, 0.25 mm internal diameter, 0.25-μm film thickness, Phenomenex, Macclesfield, UK) using He carrier gas (1.2 ml min^−1^). Injector temperature was 300 °C. The column temperature programme started at 70 °C with an increase to 310 °C at 15 °C/min and a 6 min hold at the final temperature, followed by a 7 min backflush to clean the column at the end of every run. Compounds were fragmented by electron ionisation at 70 eV and MS spectra were collected between 50 and 600 amu at a rate of 5 spectra s^−1^. To identify features, the chromatograms were deconvolved using Agilent MassHunter Qualitative Analysis (v10.0) and Agilent Unknowns Analysis (v10.0) software (Agilent Technologies, Santa Clara, CA, USA). The extracted mass spectra were searched using NIST v11 mass spectral library (National Institute of Standards and Technology, USA), the Fiehn mass spectral library (Agilent Technologies, Santa Clara, CA, USA) and the Golm mass spectral library (http://gmd.mpimp-golm.mpg.de/). The libraries were installed in MassHunter Quantitative Analysis Library Editor (v10.0) and searched from MassHunter Qualitative Analysis (v10.0) or Agilent Unknowns Analysis (v10.0) software.

### RNA isolation

Strains IPO323, IPO323ΔWco1, MC2306, MC2306ΔWco1, 1E4, 1E4ΔWco1, T5 or T5ΔWco1 were inoculated from YPD agar plates, and spores and hyphae were prepared as follows: 100 ml MM were inoculated with ~50 million spores, obtained from YPD agar plates and grown as described above. After 2 days of growth at 12 °C, 12 °C/MetOH, 18 °C, or 18 °C/MetOH, cell cultures were filtered through Miracloth (Merck Millipore, UK) and the flow-through centrifuged for 10 min (Heraeus Biofuge Stratos™, Thermo Fisher Scientific, UK). Hyphae were harvested after 2–3 days of growth at 18 °C/WLSE by filtering the cell suspension through Miracloth. The retained hyphae were washed off the Miracloth sheet by gentle shaking in 50 ml of sterile distilled water for 5 min, followed by centrifugation at 1250 × g for 10 min. Purity (>95%) was assessed microscopically prior to RNA extraction. Total RNA was prepared by grinding cells in liquid nitrogen, using an RNeasy plant mini kit (Qiagen, UK) to prepare RNA from the lyophilate, according to manufacturers’ instructions. RNA samples were treated with DNase I (Qiagen) at room temperature for 15 min to remove genomic DNA.

### Transcriptome sequencing and analysis

Library preparation and sequencing of 3–4 biological replicates per condition was undertaken by Exeter Sequencing Service (Exeter University). RNA concentration was quantified using Qubit RNA Assay kit and a Qubit 2.0 Fluorometer (Fisher Scientific, UK) and integrity was assessed using the Tapestation 4200 automated electrophoresis system (Agilent Technologies). Libraries were created from 500 ng total RNA using *TruSeq Stranded* mRNA Library Prep Kit (Illumina, USA), and mRNA was isolated using poly-A oligo-attached magnetic beads, prior to sequencing >100 base-pair reads on either the HiSeq 2500 or NovaSeq platform (Illumina). Adaptor and base quality trimming of raw FASTQ data used Cutadapt v1.13 (Supplementary Table 7 for references and web addresses of all bioinformatic programmes used in this study) Read quality was assessed with FastQC v0.11.4 and sample composition purity using FastQScreen v0.5.2. Genomic DNA of all strains (IPO323, T5, 1E4 and MC2306) was aligned to the IPO323 reference genome^25^ (MG2/GCA_000219625.1) with TopHat2 v2.1.1 and read counts generated using HTSeq-count v0.10.0 and recent annotation of IPO323^73^. Read counts were normalised and differentially expressed genes (false detection rate *P* < 0.01) between spores and hyphae, or between WT and Δ*wco*1 mutants in 18 °C/WLSE were identified using DESeq2 v1.14.1. Variation between transcriptome data sets was determined by PCA using DESeq2 v1.14.1. Difference in global transcriptional activity at 12 and 18 °C were identified by investigating the expression of 17 fungal housekeeping genes in transcriptomes (Supplementary Data 2), derived from cells grown for 2 days in MM at both temperatures. Their expression at 18 °C was compared to 12 °C and the average in per cent and the 95% confidence interval was determined, using GraphPad Prism6. Only genes that were expressed beyond these boundaries were considered further (≥1.5-fold induced, ≥1.07-fold repressed).

### Functional annotation of gene products

Gene sequences were obtained from FungiDB (https://fungidb.org/fungidb/); sequence comparisons used CLUSTAL Omega 1.2.4 and EMBOSS Needle 6.6.0. Predicted protein functions from two IPO323 annotations^25,73^ were combined with functional information from Ensembl Fungi, FungiDB, Blast2GO, Pfam and published literature. Matching equivalent accession IDs were identified using bedtools intersect before incorporating all annotations.

### Identifying secreted enzymes, effectors, plant cell wall-degrading enzymes and transcription factors

Secreted proteins were predicted by the presence of a signal peptide sequence, using SignalP5.0, or a functional prediction suggesting extracellular activity. Proteins were categorised as effectors if they (i) aligned with prediction criteria in SignalP5.0 and EffectorP2.0, (ii) are published *Z. tritici* effectors or (iii) show significant homology to published fungal effectors. Secreted enzymes were identified as carrying a SignalP5.0-positive peptide and/or belong in a pathogenicity-associated enzyme category (cell wall-degrading enzymes, cutinases, amylases, lipases, proteases) or are a homologue of a published secreted enzyme. Transcription factors were identified by functional annotation (see above) searching for indications of transcriptional regulatory activity (e.g. domains, GO-term predictions).

### Molecular cloning

Vectors were generated by in vivo recombination in *S. cerevisiae* DS94 (MATα, *ura3-52*, *trp1-1*, *leu2-3*, *his3-111* and *lys2-801*) following published procedures^74^. In brief, *S. cerevisiae* DS94 cells were grown in 3 ml YPD at 28 °C, 12 h at 200 rpm and used to inoculate 50 ml YPD and grown for 5 h at 28 °C, 200 rpm. Cells were harvested by centrifugation at 672 × g for 5 min, washed with 5 ml sterile distilled water, and after re-centrifugation, re-suspended in 300 μl sterile water. Then, 4 μl of purified DNA fragments for in vivo recombination were mixed with 50 μl salmon sperm DNA (2 mg ml^−1^ stock; Sigma-Aldrich), 50 μl *S. cerevisiae* DS94 cells, 32 μl of 1 M lithium acetate and 240 μl of 50% (v v^−1^) PEG 4000 (Sigma-Aldrich). The sample was gently mixed and incubated at 28 °C for 30 min. A heat shock was induced at 45 °C, 15 min, samples were centrifuged at 400 × g for 2 min at room temperature and the pellet was suspended in 150 μl sterile distilled water. Finally, cells were plated onto yeast synthetic drop-out medium, which lacks uracil (yeast nitrogen base without amino acids and ammonium sulphate, 1.7 g l^−1^; ammonium sulphate, 5 g l^−1^; casein hydrolysate, 5 g l^−1^; tryptophan, 20 mg l^−1^; agar, 20 g l^−1^) and incubated at 28 °C for 2 days. Plasmids were transformed into and amplified in *E. coli* strain DH5α. All restriction enzymes were obtained from New England Biolabs (Herts, UK).

Each plasmid was generated as described below. Plasmid descriptions are provided in Supplementary Table 2, cloning primers are listed in Supplementary Table 8.

#### pCP*dnaJ*-ZtGFP

This plasmid contains codon-optimised *ztgfp* under the control of the promoter of a gene encoding a homologue of heat stress protein DnaJ. It was generated using the 13,083 bp fragment of plasmid pCZtGFP^75^ (*PmI*l-digested) and 1000 bp of *dnaJ* promoter (amplified with primers SK894 and SK895 from IPO323 genomic DNA). The plasmid pCP*dnaJ*-ZtGFP was integrated into the *sdi1* locus of strain IPO323, giving strain IPO323_P*dnaJ*ZtG.

#### pCP*hsp60*-ZtGFP

This plasmid contains codon-optimised *ztgfp* under the control of the promoter of a gene encoding a homologue of heat stress protein Hsp60. The plasmid was generated using the 13,083 bp fragment of plasmid pCZtGFP^75^ (*PmI*l-digested) and 1000 bp of *hsp60* promoter (amplified with primers SK908 and SK909 from IPO323 genomic DNA). The plasmid pCP*hsp60*-ZtGFP was integrated into the *sdi1* locus of strain IPO323, giving strain IPO323_P*hsp60*ZtG.

#### pCP*hsp70*/*ssa3*-ZtGFP

This plasmid contains codon-optimised *ztgfp* under the control of the promoter of a gene encoding a homologue of heat stress protein Hsp70/Ssa3. The plasmid was generated using the 13,083 bp fragment of plasmid pCZtGFP^75^ (*PmI*l-digested) and 1000 bp of *hsp70/ssa3* promoter (amplified with primers SK896 and SK897 from IPO323 genomic DNA). The plasmid pCP*hsp70*/*ssa3*-ZtGFP was integrated into the *sdi1* locus of strain IPO323, giving strain IPO323_P*hsp70*/*ssa3*ZtG.

#### pCP*sti1*-ZtGFP

This plasmid contains codon-optimised *ztgfp* under the control of the promoter of a gene encoding a homologue of heat stress protein Sti1. The plasmid was generated using the 13,083 bp fragment of plasmid pCZtGFP^75^ (*PmI*l-digested) and 1000 bp of *sti1* promoter (amplified with primers SK906 and SK907 from IPO323 genomic DNA). The plasmid pCP*sti1*-ZtGFP was integrated into the *sdi1* locus of strain IPO323, giving strain IPO323_P*sti1*ZtG.

#### pCP*cdc37*-ZtGFP

This plasmid contains codon-optimised *ztgfp* under the control of the promoter of a gene encoding a homologue of heat stress protein Cdc37. The plasmid was generated using the 13,083 bp fragment of plasmid pCZtGFP^75^ (*PmI*l-digested) and 1000 bp of *cdc37* promoter (amplified with primers SK898 and SK899 from IPO323 genomic DNA). The plasmid pCP*cdc37*-ZtGFP was integrated into the *sdi1* locus of strain IPO323, giving strain IPO323_P*cdc37*ZtG.

#### pCP*hsp70*/*bip1*-ZtGFP

This plasmid contains codon-optimised *ztgfp* under the control of the promoter of a gene encoding a homologue of heat stress protein Hsp70/bip1. The plasmid was generated using the 13,083 bp fragment of plasmid pCZtGFP^75^ (*PmI*l-digested) and 1000 bp of *hsp70/bip1* promoter (amplified with primers SK902 and SK903 from IPO323 genomic DNA). The plasmid pCP*hsp70*/*bip1*-ZtGFP was integrated into the *sdi1* locus of strain IPO323, giving strain IPO323_P*hsp70*/*bip1*ZtG.

#### pCP*cne1*-ZtGFP

This plasmid contains codon-optimised *ztgfp* under the control of the promoter of a gene encoding a homologue of heat stress protein Cne1. The plasmid was generated using the 13,083 bp fragment of plasmid pCZtGFP^75^ (*PmI*l-digested) and 1000 bp of *cne1* promoter (amplified with primers SK912 and SK913 from IPO323 genomic DNA). The plasmid pCP*cne1*-ZtGFP was integrated into the *sdi1* locus of strain IPO323, giving strain IPO323_P*cne1*ZtG.

#### pCP*hsp70*/*ssc70*-ZtGFP

This plasmid contains codon-optimised *ztgfp* under the control of the promoter of a gene encoding a homologue of heat stress protein Hsp70/Ssc70. The plasmid was generated using the 13,083 bp fragment of plasmid pCZtGFP^75^ (*PmI*l-digested) and 1000 bp of *hsp70*/*ssc70* promoter (amplified with primers SK904 and SK905 from IPO323 genomic DNA). The plasmid pCP*hsp70*/*ssc70*-ZtGFP was integrated into the *sdi1* locus of strain IPO323, giving strain IPO323_P*hsp70*/*ssc70*ZtG.

#### pCP*ztri13.167*ZtGFP

This plasmid contains codon-optimised *ztgfp* under the control of the promoter of a gene encoding *ZTRI_13.167*, which is highly expressed in hyphae. The plasmid was generated using the 13,083 bp fragment of plasmid pCZtGFP^75^ (*PmI*l-digested) and 1000 bp of *ztri_13.167* promoter (amplified with primers CA86 and CA87 from IPO323 genomic DNA).

#### PCP*ztri7.69*mCherry

This plasmid contains *mCherry* under the control of the promoter of a gene encoding ZTRI_7.69, which is highly expressed in spores. The plasmid was generated using the 11,984 bp fragment of plasmid pCmCherry^76^ (digested with *Mlu*l and *Zra*I), 1000 bp of *ztri_7.69* promoter (amplified with primers CA102 and CA103 from IPO323 genomic DNA) and 260 bp *Tnos* terminator (amplified with primers CA98 and CA99 from plasmid pACADSB-mCherry^77^.

#### pCFDR1

This plasmid contains codon-optimised *ztgfp* under the control of the promoter of a gene encoding ZTRI_13.167, which is highly expressed in hyphae and *mCherry* under the control of the promoter of a gene encoding ZTRI_7.69, which is highly expressed in spores. The plasmid was generated using 12,382 bp fragment of plasmid pCP*ztri13.167*ZtGFP (see above; *Mlu*I/*Bgl*II digested), 1086 *tub2* terminator (amplified with primers SK297 and SK19 from pCP*ztri13.167*ZtGFP), 2015 bp of *promoter ztri7.69mCherry-Tnos*, amplified with primers CA117 and CA120 from plasmid pCP*ztri7.69*mCherry, and 889 bp of the right flank of *sdi1* (amplified with primers CA115 and CA116 from plasmid pCP*ztri7.69*mCherry). The plasmid pCFDR1 was integrated into the *sdi1* locus of strains IPO323, MC2306, 1E4 and T5 to give strains IPO323_FDR1, MC2306_FDR1, 1E4_FDR1 and T5_FDR1.

#### pH3LysMmCherry

This plasmid contains *mCherry* fused to the 3-prime end of *3lysm* and is designed for targeted integration into the native gene locus in *Z. tritici*. The plasmid was generated using 9760 bp fragment of pCZtGFP^75^(*PmI*l-digested), 518 bp of ORF, 3′ *3lysm* (amplified with primers SK208 and SK210 from IPO323 genomic DNA) and 1014 bp of *mCherry* and *Tnos* (amplified with primers SK215 and SK214 from plasmid pACADSB-mCherry^77^), 1806 bp of hygromycin resistance cassette (amplified with primers SK136 and SK137 from plasmid pCHYG^78^) and 1041 bp non-coding 3′ *3lysm* (amplified with primers SK211 and SK212 from IPO323 genomic DNA). The plasmid pH3LysMmCherry was integrated into the *3lysm* locus of strain IPO323_eGSso1^27^ to give strain IPO323_eGSso1_3LysMmCh.

#### pH1LysMmCherry

This plasmid contains *mCherry* fused to the 3-prime end of *1lysm* and is designed for targeted integration into the native gene locus in *Z. tritici*. The plasmid was generated using 9760 bp fragment of pCGEN-YR^74^ (digested with *Xba*I and *Zra*I), 392 bp of ORF, 3′ *1lysm* (amplified with primers SK302 and SK303 from IPO323 genomic DNA) and 1014 bp of *mCherry* and *Tnos* (amplified with primers SK215 and SK214 from plasmid pACADSB-mCherry^77^),1806 bp of hygromycin resistance cassette (amplified with primers SK136 and SK137 from plasmid pCHYG^78^) and 1005 bp non-coding 3′ *1lysm* (amplified with primers SK304 and SK305 from IPO323 genomic DNA). The plasmid pH1LysMmCherry was integrated into the *1lysm* locus of strain IPO323_eGSso1^27^ to obtain strain IPO323_eGSso1_1LysMmCh.

#### pCP*3lysm*ZtGFP

This plasmid contains codon-optimised *ztgfp* under the control of the promoter of a gene encoding effector 3LysM. The plasmid was generated using the 13,083 bp fragment of plasmid pCZtGFP^75^ (*PmI*l-digested) and 1000 bp of *3lysm* promoter (amplified with primers SK314 and SK315 from IPO323 genomic DNA). The plasmid pCP*3lysm*ZtGFP was integrated into the *sdi1* locus of strain IPO323_mChSso1^79^ to give strain IPO323_mChSso1_P*3lysm*ZtG.

#### pCP*nis1*ZtGFP

This plasmid contains codon-optimised *ztgfp* under the control of the promoter of a gene encoding necrosis-inducing factor Nis1. The plasmid was generated using the 13,083 bp fragment of plasmid pCZtGFP^75^ (*PmI*l-digested) and 1000 bp of *nis1* promoter (amplified with primers SK573 and SK574 from IPO323 genomic DNA). The plasmid pCP*nis1*ZtGFP was integrated into the *sdi1* locus of strain IPO323_mChSso1^79^ to give strain IPO323_mChSso1_P*nis1*ZtG.

#### pCP*nip1*-ZtGFP

This plasmid contains codon-optimised *ztgfp* under the control of the promoter of a gene encoding necrosis-inducing factor Nip1. The plasmid was generated using the 13,083 bp fragment of plasmid pCZtGFP^75^ (*PmI*l-digested) and 1000 bp of *nip1* promoter (amplified with primers SK882 and SK883 from IPO323 genomic DNA). The plasmid pCP*nip1*-ZtGFP was integrated into the *sdi1* locus of strain IPO323_mChSso1^79^ to give strain IPO323_mChSso1_P*nip1*ZtG.

#### pCP*1lysm*ZtGFP

This plasmid contains codon-optimised *ztgfp* under the control of the promoter of a gene encoding effector 1LysM. The plasmid was generated using the 13,083 bp fragment of plasmid pCZtGFP^75^ (*PmI*l-digested) and 1000 bp of *1lysm* promoter (amplified with primers SK316 and SK317 from IPO323 genomic DNA). The plasmid pCP*1lysm*ZtGFP was integrated into the *sdi1* locus of strain IPO323_mChSso1^79^ to give strain IPO323_mChSso1_P*1lysm*ZtG.

#### pCP*ecp2.2*ZtGFP

This plasmid contains codon-optimised *ztgfp* under the control of the promoter of a gene encoding effector Ecp2.2. The plasmid was generated using the 13,083 bp fragment of plasmid pCZtGFP^75^ (*PmI*l-digested) and 1000 bp of *ecp2.2* promoter (amplified with primers SK577 and SK578 from IPO323 genomic DNA). The plasmid pCP*ecp2.2*ZtGFP was integrated into the *sdi1* locus of strain IPO323_mChSso1^79^ to give strain IPO323_mChSso1_P*ecp2.2*ZtG.

#### pHΔWco1

This plasmid is used to replace the endogenous *wco*1 gene with the hygromycin resistance cassette. The plasmid was generated using 9760 bp fragment of pCGEN-YR^74^ (digested with *Xba*I and *Zra*I), 982 bp of *wco*1 promoter (amplified with primers SK699 and SK700 from IPO323 genomic DNA), 1806 bp of hygromycin resistance cassette (amplified with primers SK136 and SK137 from plasmid pCHYG^78^) and 994 bp non-coding 3′ *wco*1 (amplified with primers SK701 and SK702 from IPO323 genomic DNA). The plasmid pHΔWco1 was integrated into the *wco*1 locus of strains IPO323, MC2306, 1E4, T5, IPO323_FDR1, MC2306_FDR1, 1E4_FDR1, T5_FDR1 to give strains IPO323_ΔWco1, MC2306_ΔWco1, 1E4_ΔWco1, T5_ΔWco1, IPO323_FDR1_ΔWco1, MC2306_FDR1_ΔWco1, 1E4_FDR1_ΔWco1 and T5_FDR1_ΔWco1.

#### pHWco1Δ^LOV^

This plasmid is designed to delete the endogenous *lov* domain in *wco*1 gene. The plasmid was generated using 9533 bp fragment of pCZtGFP^75^ (digested with *BamH*I and *Hind*III), 2291 bp of *wco*1 promoter and 5′ end of *wco*1 gene (amplified with primers SK840 and SK833 from IPO323 genomic DNA), 2289 bp of 3′ end of *wco*1 gene and *wco*1 terminator (amplified with SK834 and SK841 from IPO323 genomic DNA), 1806 bp of hygromycin resistance cassette (amplified with primers SK136 and SK137 from plasmid pCHYG^78^) and 525 bp covering the right flank of *wco*1 gene (amplified with primers SK842 and SK702 from IPO323 genomic DNA). The plasmid pHWco1Δ^LOV^ was integrated into the *wco*1 locus of strain IPO323_FDR1, giving strain IPO323_FDR1_Wco1Δ^LOV^.

#### pHWco1

This plasmid contains the full-length *wco*1 gene under the control of its native promoter, for random ectopic integration. This plasmid was generated using 14,086 bp fragment of pHmCherrySso1^27^ (digested with *Xba*I and *Zra*I), 1149 bp *tub2* promoter (amplified with SK46 and SK706 from IPO323 genomic DNA), 3474 bp full-length *wco*1 gene (amplified with primers SK707 and SK715 from IPO323 genomic DNA) and 994 bp *wco*1 terminator (amplified with primers SK714 and SK713 from IPO323 genomic DNA). The plasmid pHWco1 was random ectopically integrated into strain IPO323_FDR1_DSD1 to give strain IPO323_FDR1_DSD1_Wco1.

#### pGΔWco1

This plasmid is designed to delete the endogenous *wco*1 gene by replacing it with the G418 resistance cassette. The plasmid was generated using 11,484 bp of plasmid pHΔWco1 (see above; digested with *Afl*II) and 1424 bp of G418 resistance cassette (amplified with primers SK772 and SK773 from plasmid pCGen^80^). The plasmid pGΔWco1 was integrated into the *wco*1 locus of strains IPO323_mChSso1_P*1lysm*ZtG and IPO323_mChSso1_P*3lysm*ZtG to obtain strains IPO323_mChSso1_P*1lysm*ZtG_ ΔWco1 and IPO323_mChSso1_P*3lysm*ZtG_ΔWco1.

### *Z. tritici* transformation and molecular analysis of transformants

Plasmids were transformed into *A. tumefaciens* strain EHA105 and *A. tumefaciens-* mediated *Z. tritici* transformation performed as described^30^. In brief, 1 μl vector DNA was added to 1 × 10^5^ *A. tumefaciens* cells, heat shocked at 37 °C for 5 min and retained on ice for 5 min. After the addition of 1 ml DYT medium, samples were incubated at 28 °C, at 200 rpm for 3–4 h, followed by plating on DYT agar medium, supplemented with 20 μg ml^−1^ rifampicin (Melford, Ipswich, UK) and 50 μg ml^−1^ kanamycin (Sigma-Aldrich). Positive transformants were identified by colony PCR and grown in 10 ml DYT medium/20 μg ml^−1^ rifampicin/50 μg ml^−1^ kanamycin overnight at 28 °C, 200 rpm. Cells from this culture were used to inoculate 10 ml *Agrobacterium* induction medium (AIM; 10 mM KH_2_PO_4_, 10 mM K_2_HPO_4_, 2.5 mM NaCl, 2 mM MgSO_4_⋅7 H_2_O, 0.7 mM CaCl_2_, 9 mM FeSO_4_, 4 mM (NH4)_2_SO_4_, 10 mM glucose, 0.5% (w v^−1^) glycerol, 40 mM MES buffer, 1 l H_2_O, pH 5.6), supplemented with 200 μM acetosyringone (Sigma-Aldrich) and grown at 28 °C, 200 rpm, until the initial OD_660_ (optical density at 660 nm) of 0.15 reached to 0.3–0.35 (4–5 h). *Z. tritici* spores were harvested from 5-day-old YPD plates and diluted to a concentration of 1 × 10^8 ^ml^−1^ in AIM. Equal volumes of the *Z. tritici* and *A. tumefaciens* cultures were combined and 200 μl of this mixture was plated onto nitrocellulose filters (AA packaging limited, Preston, UK), overlaid onto AIM agar plates (AIM, 2% agar, w v^−1^), supplemented with 200 μM acetosyringone. After growth for 3 days at 18 °C, the filters were transferred onto Czapek Dox agar plates (Oxoid, Basingstoke, UK), containing 100 μg ml^−1^ cefotaxime (Melford), 100 μg ml^−1^ timentin (Melford) and either 40 μg ml^−1^ carboxin (Sigma-Aldrich), 200 μg ml^−1^ hygromycin (Invivogen, Toulouse, France) or 200 μg ml^−1^ G418 (Sigma-Aldrich) and incubated at 18 °C until colonies appeared (8–12 days). Colonies were transferred onto YPD agar plates, containing 100 μg ml^−1^ cefotaxime, 100 μg ml^−1^ timentin and either 40 μg ml^−1^ carboxin or 200 μg ml^−1^ hygromycin or 200 μg ml^−1^ G418 and grown at 18 °C for 3–4 days.

Integration of vectors into the native loci of *1lysm*, *3lysm*, *wco*1 or the ectopic *sdi1* locus^30^ was by restriction enzyme digestion (*Xmn*I for *1lysm* and *3lysm*, *Bgl*II for *sdi1* locus, *Sph*I to confirm *wco*1 deletion and LOV domain deletion in *wco*1) and confirmed by Southern blot analyses, with DNA-probes generated by PCR and labelled using PCR DIG Probe Synthesis Kit (Sigma-Aldrich, UK).

### Microscopy techniques

Widefield microscopy was performed using a motorised inverted microscope (IX81/IX83; Olympus, Germany), with a PlanApo 100x/1.45 Oil TIRF, UPlanSApo 60x/1.35 Oil or UApoN340 40×/1.35 Oil objective (Olympus) and a VS-LMS4 Laser Merge System for laser-based excitation (488 nm 75 mW and 561 nm 75 mW; Visitron Systems, Germany). Images were captured using a CoolSNAP HQ2 camera (Photometrics, USA). All parts were controlled by MetaMorph 7.8× (Molecular Devices, UK) or VisiView 3.3.0.4 (Visitron Systems). General bright field DIC microscopy used built-in illumination.

The cell length and width were determined using strain IPO323_eGSso1, which expresses a green-fluorescent plasma membrane marker^27^. Cell length and width were determined from microscopy images.

The induction of heat stress-related promoters used strains IPO323_P*dnaj*ZtG, IPO323_P*hsp60*ZtG, IPO323_P*hsp70/ssa3*ZtG, IPO323P*sti1*ZtG, IPO323_P*cdc37*ZtG, IPO323_P*hsp70/bip1*ZtG, IPO323_P*cne1*ZtG and IPO323_P*hsp70/ssc70*ZtG. Strains were grown for 2 days at 18 °C in a YG medium and cultures were divided equally into three flasks and incubated at 18, 27 and 34 °C for 3 h. Cells were imaged using the Olympus IX83 microscope and the 488 nm laser at the same laser intensity to compare GFP expression at different temperatures in each strain. The average cytoplasmic GFP intensity was measured and corrected for adjacent image background, using Metamorph.

Spinning disc confocal microscopy used a VisiScope Confocal Cell Explorer (Visitron System)—an IX81 motorised inverted microscope (Olympus), a CSU-X1 Spinning Disc unit (Yokogawa, Japan), a UPlanSApo63/1.35 oil objective (Olympus) and a Photometrics CoolSNAP HQ2 camera (Photometrics). Fluorescent tags were excited using a VS-LMS6 Laser Merge System (488 nm/100 mW and 561 nm/100 mW, Visitron System). Co-observation of green and red fluorescence used an OptoSplit II LS Image Splitter (Cairn Research Limited, UK). The system was controlled by VisiView (Visitron System).

To quantify the expression of cytoplasmic GFP from various promoters on and in planta, leaves were infected with reporter strains constitutively expressing the red-fluorescent plasma membrane marker mCherry-Sso1 and carrying codon-optimised ZtGFP, controlled by 1000 bp promoters of the chosen effector genes. At 0 and 2 dpi, leaves were submerged briefly in Flutec PP11 (F2 Chemicals Ltd., UK), placed on Carolina Observation Gel (Carolina Biological Supply Company, USA) and green fluorescence average signal intensity was analysed by MetaMorph, using the mCherry-Sso1 signal to delineate cell boundaries. Measured values were corrected for image background.

Laser scanning confocal microscopy of infected leaves used a Leica TCS SP8 confocal laser scanning microscope (Leica), equipped with a Blue diode, Argon, DPSS561, HeNe594 and a HeNe633 laser, three hybrid detectors and two conventional PMT detectors, and HC PL APO CS2 63×/1.40 oil and HC PL APO 63×/1.2 water objectives. Image processing used Leica LAS AF X 3.5.2.18963 software.

Lectin staining used IPO323 infected leaf sections cleared overnight in 100% ethanol, 1 h incubation in 10% KOH at 85 °C, washed in phosphate-buffered saline (PBS, pH 7.4), stained with 20 µg ml^−1^ propidium iodide/ 10 µg ml^−1^ WGA-Alexa-Fluor 488 (Sigma-Aldrich) in PBS/0.02% (v v^−1^) Tween20 for 30 min, followed by washing in PBS.

Imaging of living hyphae on or in wheat leaves, at various time points after infection (for infection protocol see Plant-infection assays), used cytoplasmic or plasma membrane-located GFP excited at 488 nm (Argon laser) and detected at 495–525 nm (HyD detectors in photon integration mode). Chloroplast auto-fluorescence was detected at 700–790 nm; leaf epidermis was visualised using a UV laser at 405 nm and detection at 411–486 nm, using PMT detectors. Stomata were analysed using SP8 confocal laser scanning microscopy, with plants exposed to 12 h full light or 12 h in darkness, under conditions identical to our described plant-infection assays.

### FDR1-expression experiments

The effect of temperature and WLSE on hyphal programme induction used strains IPO323_FDR1, IPO323_FDR1_ΔWco1, IPO323_FDR1_Wco1Δ^LOV^; MC2306_FDR1, MC2306_FDR1_ΔWco1; 1E4_FDR1, 1E4_FDR1_ΔWco1; T5_FDR1 and T5_FDR1_ΔWco1. Cells were grown in MM or MM without glucose (carbon-free condition) or without NO_3_ (nitrogen-free condition) for 2–3 days, at 200 rpm, at 12 °C in cooling shaking incubators (MaxQ 6000, Thermo Scientific). A standard daylight regime was applied. Cells grown at 15 °C and above were incubated at 200 rpm in an Innova 44 incubator (New Brunswick Scientific), again exposing them to a standard daylight regime. To test the effect of WLSE, 0.5% (v v^−1^) of freshly prepared WLSE/MetOH (see above for preparation) was added to 10 ml spore cultures and incubated for 2–3 days, at 200 rpm, at 12 °C. Control experiments used 0.5% (v v^−1^) methanol only. IPO323_FDR1 was also grown at 12, 15, 18, 21, 24 and 27 °C (as above). Cells were placed on 2% (w v^−1^) agarose pads and imaged using an Olympus IX81 microscope. The GFP signal background resulting from basal expression of the hyphal promoter-GFP was calculated using the software package MetaMorph, measuring the average intensity of 10 yeast-like cells, showing a strong red signal clearly identifying them as spores (~5000 arbitrary units of signal intensity). The resulting average value was used as the threshold for all green images and adjusted images were overlaid and red and green cells were counted. Morphotypes showing a clear green-fluorescent signal were scored as being in hyphal induction mode (including those that showed residual red fluorescence). Individual hyphae were counted within a single image field as discrete entities, irrespective of their branching pattern.

### Cellulase and xylanase activity assays

Five million IPO323 cells were grown in 20 ml MM for 2 days at 200 rpm at 12 °C (spores). This pre-culture was split equally and supplemented with either 50 µl WLSE stock solution, grown for 2 days at 18 °C, 200 rpm, or supplemented with 50 µl MetOH (Control), grown for 2 days, 200 rpm at 12 °C, 200 rpm. The cultures were centrifuged for 5 min at 16,200 × g and 50 μl supernatant was used to determine cellulase or xylanase activity in a fluorometric Cellulase Activity Assay kit (Abcam, UK) or an EnzChek Ultra Xylanase Assay Kit (Thermo Fisher Scientific), following the manufacturers’ instructions. After 24 h incubation at room temperature in the dark fluorescence was measured using a GloMax® Discover plate reader (Promega, USA).

### ATMT mutagenesis for factors involved in dimorphic switching

*Agrobacterium*-mediated transformation of *Z. tritici* stain IPO323_FDR1 was performed as described above, using vector pCGen, which carries a G418 resistance cassette for random ectopic integration^74^. Transformants were replica-plated onto PDA and hyphal growth was induced at 25 °C for 5 days. Colonies defective in hyphal growth were identified by their smooth appearance on a Stemi 2000-C Stereo Microscope (Carl Zeiss GmbH, Germany). To confirm this phenotype, smooth colonies were re-suspended in ddH_2_O and re-plated on PDA for 5 days at 25 °C. Hyphal growth-defective mutants were grown in PDB for 2 days at 25 °C, 150 rpm and their ability to switch, as revealed by use of FDR1 marker, was assayed microscopically. Mutants impaired in the expression of GFP (>50% cells show no green fluorescence) were tested in planta by evenly spraying 100 µl cell suspension/leaf (cell density of 1.0 × 10^6^) onto a total of 10 wheat leaves (12 days old and 12–14 cm each). Inoculated plant tissue was examined by confocal laser scanning microscopy and hyphal hyphae formation and expression of green fluorescence was quantified, as described above (FDR1-expression experiments). Maximum projection images were generated using LasX 3.5.2.18963 software (Leica). The basal GFP expression of hyphal promoter-GFP was calculated as described above. Fluorescent cells were counted, and the percentage of green cells was calculated. Mutant cells impaired in the hyphal formation and strongly impaired in GFP expression were considered DSD.

### Genome sequencing of *Z. tritici* strains and DSD mutant analysis

Strains MC2306 and T5 were grown in YG medium for 3 days at 18 °C, 200 rpm. Genomic DNA was isolated from *Z. tritici*, grown in YG medium for 3 days at 18 °C, 200 rpm. Three ml of cells were harvested by centrifugation (Micro Star 17 R cooled centrifuge, VWR) at 16,500 × g for 2 min, followed by the addition of 400 μl of lysis buffer (2% (v v^−1^) Triton X, 1% (v v^−1^) SDS, 100 mM NaCl, 10 mM Tris HCl pH-8.0, 1 mM EDTA), 500 μl phenol: chloroform (1:1) and acid-washed glass beads added (425–600 μm; Sigma-Aldrich). Samples were mixed for 10 min using an IKA Vibrax shaker (IKA, Staufen, Germany) and centrifuged (Micro Star 17R cooled centrifuge, VWR) at 4 °C for 10 min at 16,500 × g. The supernatant was mixed with 1 ml 100% ethanol and centrifuged again. The sedimented DNA was washed with 500 μl 70% ethanol and dried at 55 °C for 5 min. The DNA was suspended in 50 μl sterile distilled water. For PCR applications, the genomic DNA was diluted 200 times and sequenced at Exeter Sequencing Service (https://biosciences.exeter.ac.uk/sequencing/).

DSD mutants were genome-sequenced to identify insertion points of the G418 resistance cassette, and other mutations (e.g., short deletions). Genomic DNA was isolated from mutant strain grown in YG medium for 3 days at 18 °C, 200 rpm, using pooled preparations, that is three pools with 4–7 strains per pool. Pooled DNA libraries were generated using the Nextflex8 protocol and as 125 base-pair paired-end reads on an Illumina HiSeq 2500 (Illumina). Quality control was undertaken as described in the section Transcriptome analysis.

The G418 resistance cassette sequence was added to the IPO323 reference genome prior to the generation and processing of trimmed-read alignment files using BWA-mem and Picard (SamFormatConverter, FixMateInformation and MarkDuplicates). G418 resistance cassette integration sites were identified as inter-chromosomal translocations using customised python scripts. Putative insertion sites were visualised in IGV 2.4.10 and confirmed experimentally by PCR and sequencing.

### Identification of Wco1-regulated genes and WCC-binding motifs

Deletion strains IPO323ΔWco1, T5ΔWco1, 1E4ΔWco1 and MC2306ΔWco1 and respective WT strains were grown under hyphal-inducing conditions (2–3 days, 18 °C/WLSE and 12 °C/MetOH). Genes were categorised as *wco*1 regulated if their typical regulation is significantly altered in the deletion strain, relative to the WT. For example, if a gene was upregulated in IPO323, grown at 18 °C/WLSE relative to 12 °C/MetOH, but is then downregulated in IPO323 ΔWco1, grown at 18 °C/WLSE relative to IPO323 18 °C/WLSE (and vice versa), it would be said to be under *wco*1 control. Putative white-collar binding site motifs in the promoters of genes predicted to be regulated by Wco1, were identified in 1000 bp upstream of open reading frames, using FIMO v5.1.0 and WCC-binding motif (MA1437.1) defined in the JASPAR database. Identified motifs (*P* < 1e–4) on the coding strand were counted.

### Plant-infection assays

*Z. tritici* strains IPO323, MC2306, 1E4, T5, IPO323_FDR1, DSD mutants (IPO323_FDR1_DSD1 to DSD14), IPO323_FDR1_ΔWco1, IPO323_FDR1_DSD1_Wco1, IPO323_FDR1_Wco1^ΔLOV^ were grown on YPD for 5 days at 18 °C. Infection of 12–14-day-old wheat plants was by spraying 1 ml of cell suspension, (adjusted to 2 × 10^5^ cells ml^−1^ using Cellometer Auto 1000 cell counter; Nexcelom Biosciences) at 5 psi using a Voilamart AS18 airbrush/compressor (Hydirect, China) onto the second leaf. Plants were covered by transparent plastic bags for 3 days to maintain high humidity and leaves were harvested after incubation in a Fitotron SGC120 growth chamber (Weiss Technik UK, UK) under 14 h light (intensity 500 μmol of PAR), 24 °C, 80% RH; 10 h dark, 20 °C, 80% RH with automated watering. At 18 dpi second leaves were detached and incubated for 3 days in sealed containers at 25 °C and scanned using an Epson Perfection V850 Pro scanner (Epson, UK). Melanised pycnidia coverage was determined using software ImageJ (https://imagej.net/Downloads) and compared to the total leaf area. Plants for FRD1-expression analysis were grown at 18 °C prior to infection by IPO323_FDR1.

To assess the impact of synchronised and asynchronised growth conditions on virulence, plants were grown for 14 days using a 12 h light cycle (80%RH, 24 °C, light intensity 500 μmol of photosynthetically active radiation) and 12 h darkness (80%RH, 20 °C). In parallel, IPO323 cells were grown at 18 °C on YPD plates for 10 days under 12 h light/dark cycle (synchronous to the plants) or at a 12 h shifted light/dark cycle (asynchronous to the plants). Fungal cells were illuminated by Full Spectrum LED Grow Light Bulbs E27 (400–740 nm, Relassy, https://www.relassy.com/). Plants were infected at the beginning of the plant dark period and analysed as described above. All plant-infection assays were performed using wheat cultivar Galaxie, except for data shown in Supplementary Fig. 6, where the wheat cultivar Consort was used.

### Reporting summary

Further information on research design is available in the Nature Research Reporting Summary linked to this article.

## Supplementary information

Supplementary Information

Supplementary Data 1

Supplementary Data 2

Supplementary Data 3

Supplementary Data 4

Supplementary Data 5

Supplementary Movie 1

Supplementary Movie 2

Supplementary Movie 3

Supplementary Movie 4

Supplementary Movie 5

Supplementary Movie 6

Reporting Summary

## Data Availability

The authors confirm that all relevant data are included in the paper or in the Supplementary information file. Source data, necessary to interpret, verify and extend the research in the article, can be accessed in the Source Data file, provided with this paper, or are accessible from the links provided herein. Raw sequencing data are available from the NCBI Sequence Read Archive (https://www.ncbi.nlm.nih.gov/bioproject/PRJNA759417), 3-h canopy temperate data are available from JRA-55 via Research Data Archive (https://rda.ucar.edu/datasets/ds628.0/). The IPO323 reference genome data are accessible at https://fungi.ensembl.org/Zymoseptoria_tritici/Info/Index. Source Data are provided with this paper.

## Source data

Source Data

## Code availability

The custom R code used for the geospatial temporal climate analysis is available at https://github.com/thomaschaloner/Zymoseptoria-tritici-white-collar-complex-integrates-light-temperature-and-plant-cues.
