# Supplementary material for: Zymoseptoria tritici white-collar complex integrates light, temperature and plant cues to initiate dimorphism and pathogenesis
Source: Nat Commun. 2022 Sep 26;13:5625. doi: 10.1038/s41467-022-33183-2 (PMC9512790; doi:10.1038/s41467-022-33183-2)
Supplement: Supplementary file 1 — Supplementary Information [file 41467_2022_33183_MOESM1_ESM.pdf]

Supplementary Information

Kilaru, S., Fantozzi, E., Cannon, S., Schuster, M., Chaloner, T.M., Guiu-Aragones, C., Gurr, S.J., Steinberg, G.

<u>Contents</u>

Supplementary Figures and Figure Legends

Supp. Figure 1: Cytology of spores and hyphae of *Z. tritici*.

Supp. Figure 2: Expression of putative heat stress genes and days at ≥18ºC in wheat fields.

Supp. Figure 3: FDR1-driven fluorescent protein expression at 12ºC - 27ºC and *on planta*.

Supp. Figure 4: Growth curves for *Z. tritici* IPO323, grown in MM at 12ºC and 18ºC.

Supp. Figure 5: GC-MS chromatograms of WLSE and blank control.

Supp. Figure 6: Pathogenicity of strains IPO323, MC2306, 1E4 and T5.

Supp. Figure 7: Comparison of putative fungal photo-receptors.

Supp. Figure 8: Estimation of ATMT gene insertion probability in *Z. tritici*.

Supp. Figure 9: Characterisation of the LOV domain in Wco1 from *Z. tritici.*

Supp. Figure 10: Dimorphic switching of IPO323 wildtype and Δ*wco1* mutants.

Supp. Figure 11: Pathogenicity of DSD mutants with insertions in *wco1* and *wco2.*

Supplementary Tables

Supp. Table 1: *Z. tritici* strains, used in this study.

Supp. Table 2: Plasmids, used in this study.

Supp. Table 3: Experimental usage of *Z. tritici* strains.

Supp. Table 4: Putative *Z. tritici* heat shock proteins tested in this study.

Supp. Table 5: Published dimorphism genes in *Z. tritici.*

Supp. Table 6: Transcription factors with roles in fungal development.

Supp. Table 7: Bioinformatic tools, used in this study.

Supp. Table 8: Primers used in this study.

Supplementary References

Supplementary Figures and Figure Legends

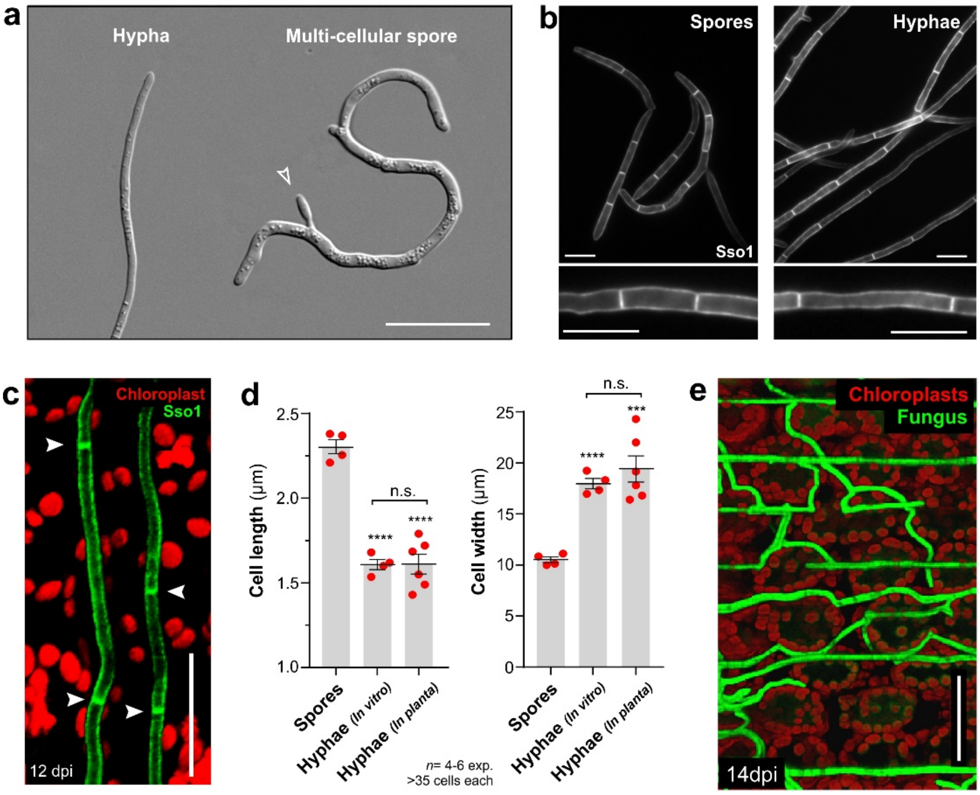

Supplementary Figure 1 Cytology of spores and hyphae of *Z. tritici*.

a A hypha and multi-cellular spore, grown for 5 days at 21°C. Arrowhead indicates a newly-forming blastospore. Scale bar= 20 µm.

b Liquid culture grown spores and hyphae, expressing plasma membrane reporter eGFP-Sso1. Lower panels show the plasma membrane of individual cells. Scale bars= 10 µm.

c Confocal image delineating the wheat leaf mesophyll, invaded by an eGFP-Sso1-expressing *Z. tritici* strain (green), at 12 dpi. Chloroplasts are shown in red; arrowheads indicate septa. Scale bar= 20 µm. See Supplementary Movie_Data 2.

d Cell width and length measured in GFP-Sso1-expressing spores, in liquid culture-grown hyphae and in plant-colonising hyphae. Cells were grown at 18°C (Spores), at 27°C (Hyphae, *in vitro*) or on the surface of the plant leaf for 2-6 dpi and in host mesophyll for 6–13 dpi (Hyphae, *in planta*).

e Confocal image at the upper level of the mesophyll in a wheat leaf, invaded by a cytoplasmic GFP-expressing strain of *Z. tritici* at 14 dpi (green). Chloroplasts are shown in red. Scale bar= 30 µm. See Supplementary Movie_Data 3.

Results shown in (**a** - **c**) where obtained independently in 2 experiments (**a**), in 4 experiments (**b**) and in >5 experiments (**c**, **e**). Values in (**d**) are shown as mean ± SEM, with n= 4-6 biologically independent experiments, each with >35 cells examined. Red dots represent mean values of individual experiments. Statistical analyses used Student's t-testing; n.s.= non-significant difference at two-tailed P= 0.9754 (cell length) and two-tailed P= 0.3311 (cell width); ***= significant different from spores at two-tailed P= 0.0007; ****= significant different from spores at two-tailed P< 0.0001. Source data are provided as a Source Data file.

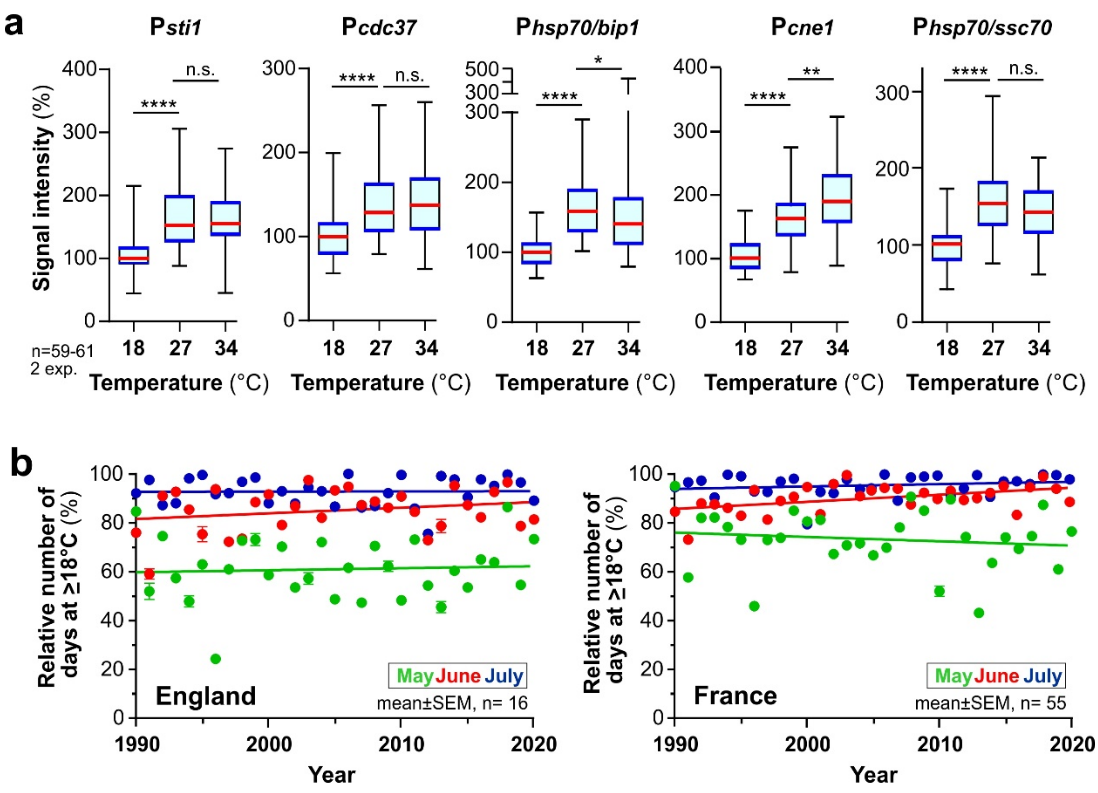

Supplementary Figure 2 Expression of putative heat stress genes and days at ≥18ºC in wheat fields.

a Signal intensity in IPO323 cells, expressing ZtGFP under the promoters of genes, encoding the putative heat stress proteins Sti1 (P_{sti1}), Cdc37 (P_{cdc37}), Hsp70/Bip1 ($P_{hsp70/bip1}$), calnexin (P_{cne1}), Hsp70/ssc70 ($P_{hsp70/ssc70}$), which are all induced.

FungiDB gene IDs are: Sti1, ZTRI_1.122; Cdc37, ZTRI_3.300; Hsp70/Bip1, ZTRI_1.1084; Cne1, ZTRI_4.802; Hsp70/Ssc70' ZTRI_5.654. All heat stress promoters are induced at 27ºC and 34ºC. See Supplementary Table 4.

b Relative number of days in May, June and July (1990-2020) during which the temperature in wheat fields in England (left) and France (right) reaches at least 18ºC. For areas of temperature sampling see red boxes in Figure 1f.

Most of the data sets in (**a**) are non-normally distributed (Shapiro-Wilk test, $P<0.05$) and thus are shown as Whiskers' plots with 25/75 percentiles (blue line) and median (red line; for all numerical values see Source Data file); in (**a**) n= 60-61 cells examined over 2 independent experiments; in (**b**) n= 16 (England) or 55 (France) measurements in different area grids, as depicted in Fig. 1f (main text). statistical comparison used Mann-Whitney, comparisons are indicated by brackets; n.s.= non-significant difference at two-tailed P= 0.5668 (P_{sti1}), 0.5492 (P_{cdc37}) and 0.0782 ($P_{hsp70/ssc70}$); *= significant difference at two-tailed P= 0.0364 ($P_{hsp70/bip1}$); **= significant difference at two-tailed P= 0.0047; ****= significant difference at two-tailed $P<0.0001$; sample sizes are indicated in graphs.

Source data are provided as a Source Data file.

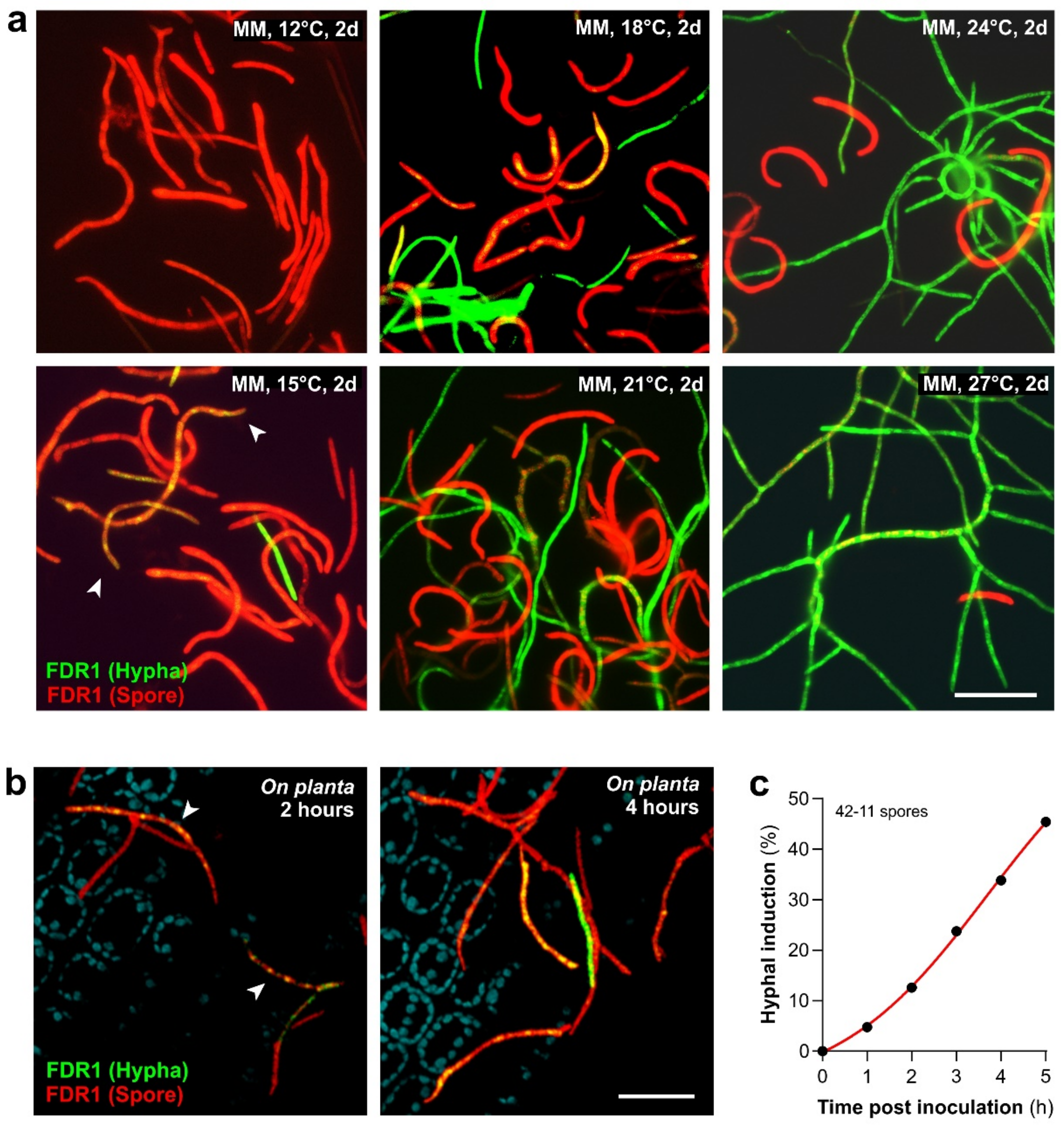

Supplementary Figure 3 FDR1-driven fluorescent protein expression at 12°C - 27°C and *on planta*.

a Spores of strain IPO323_FDR1 were grown for 2d in MM at 12°C, 15°C, 18°C, 21°C, 24°C and 27°C. Spores contain mCherry (red), whereas hyphae express eGFP (green). Scale bar= 30 µm.

b Expression of mCherry (red) and eGFP (green) from FDR1 in spores of strain IPO323_FDR1, grown at 12°C and inoculated onto wheat leaves at 18°C for 2 h. Scale bar= 30 µm.

c Graph showing the percentage of spores that display GFP expression from the FDR1 marker. Spores were grown at 12°C and inoculated onto the leaf surface of plants, grown at 18°C. Note that the switch from a spore to the hyphal programme begins at 1 h.

Results shown in (**a**) where obtained independently in 4 experiments, in (**b**) in 2 experiments. Data points in (**c**) represent counts obtained from a single representative experiment. Source data are provided as a Source Data file.

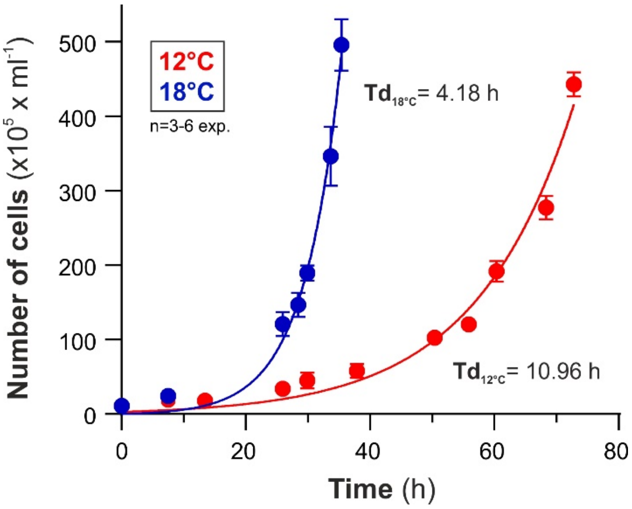

Supplementary Figure 4 Growth curves for *Z. tritici* IPO323, grown in MM at 12ºC and 18ºC. Cell numbers were determined using automated cell counting (see Methods) and doubling times ($Td_{12ºC}$ and $Td_{18ºC}$) were calculated using GraphPad Prism9. Each data point represents mean ± SEM, with n= 3-6 biologically independent experiments. Source data are provided as a Source Data file.

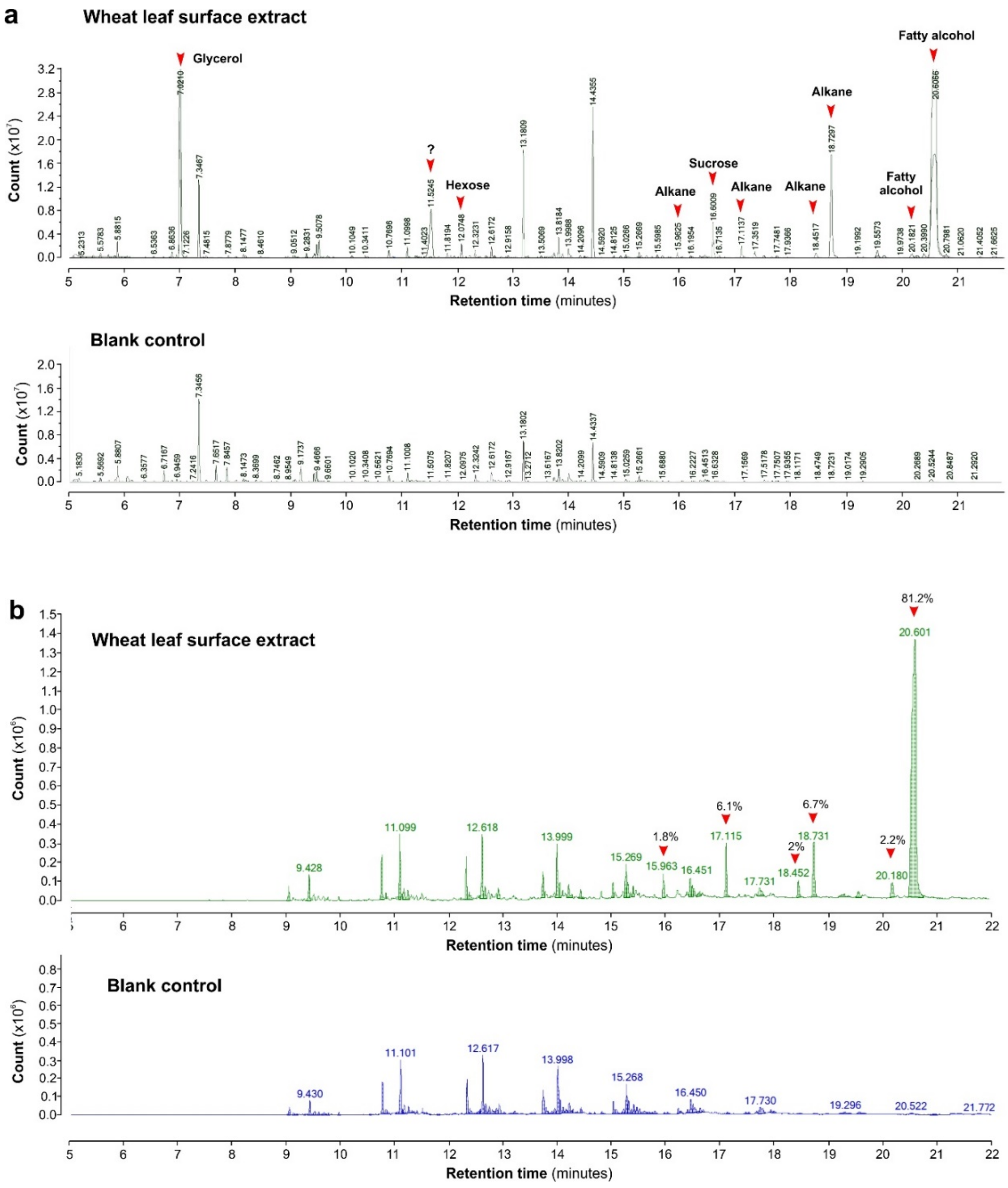

Supplementary Figure 5 GC-MS chromatograms of WLSE and blank control.

a Total ion chromatograms from GC-MS analysis of a leaf chloroform wash (Wheat leaf surface extract) compared to blank control. Proposed chemical nature indicated above peak”. “?” indicates unknown compound.

b Extracted ion chromatogram (m/z 71.08) from GC-MS analysis of a leaf chloroform wash (Wheat leaf surface extract) compared to blank control. Relative amounts indicated above peaks. Source data are provided as a Source Data file.

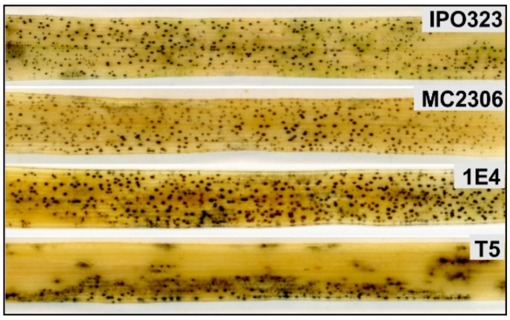

Supplementary Figure 6 Pathogenicity of strains IPO323, MC2306, 1E4 and T5.

Septoria tritici blotch disease symptoms on wheat leaves (cultivar Consort), infected with the wildtype strains IPO323, MC2306, 1E4 and T5, at 21-28 days post infection. Infection induces formation of fruiting bodies (pycnidia). T5 shows only weak infection symptoms, with fewer pycnidia formed. Results shown were obtained independently in 2 experiments. Source data are provided as a Source Data file.

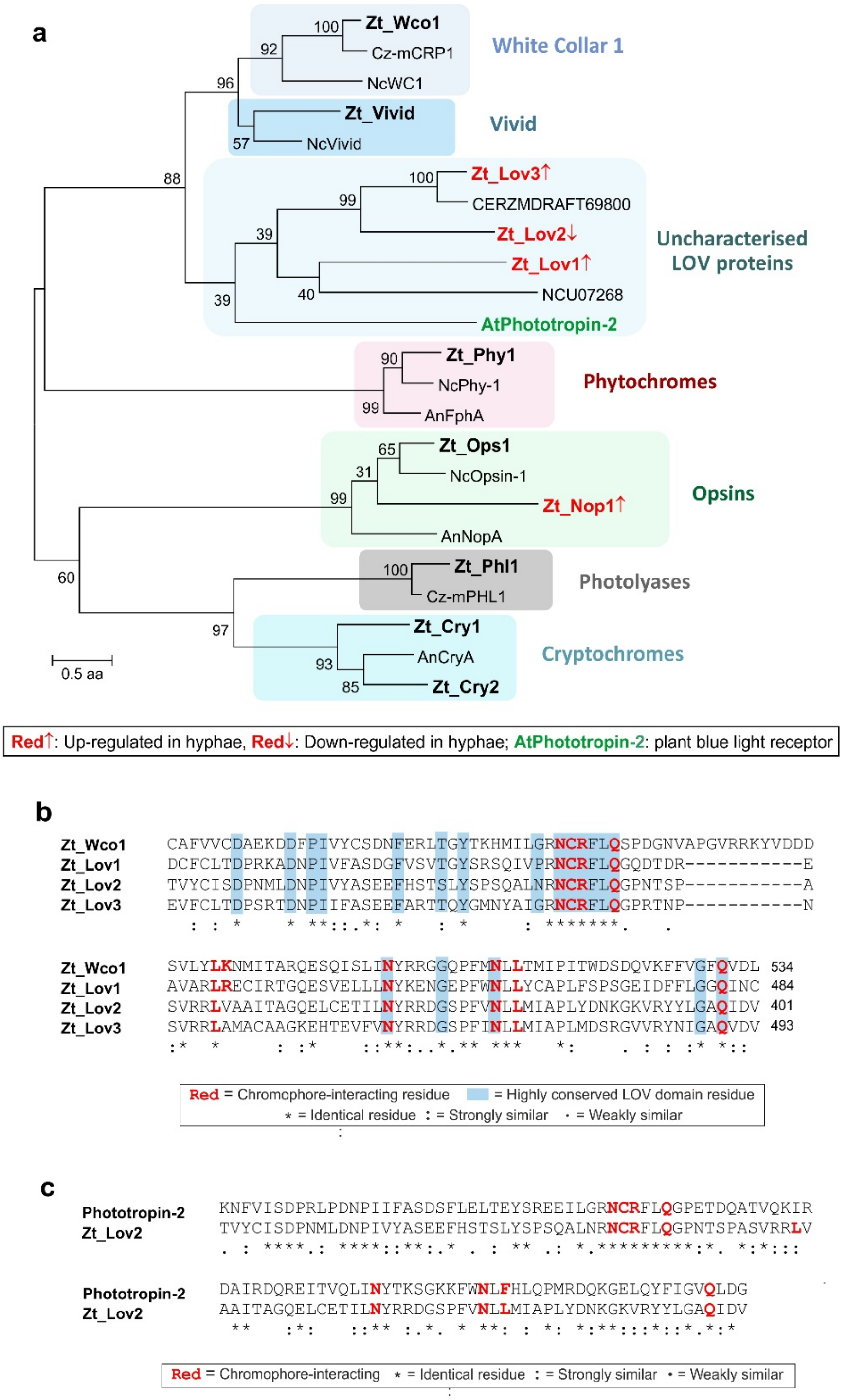

Supplementary Figure 7 Comparison of putative fungal photo-receptors.

a Phylogenetic maximum-likelihood tree of putative light-receptors in *Z. tritici*, related filamentous fungi and *Arabidopsis thaliana*. The tree was calculated using standard settings and 1000 bootstrap reiterations, using the programme MEGA 5.2[1]. Names and NCBI identifiers of predicted proteins are: (1) *Aspergillus nidulans*= AnFphA (XP_682277.1), AnNopA (CBF82866.1), AnCryA (XP_657991.1); (2) *Neurospora*

crassa= NcWC-1 (S69206), NcVivid (XP_009854479.1), NCU07268 (XP_962122.3), NcPhy-1 (XP_960393.2), NcOpsin-1 (XP_959421.1); (3) *Cercospora zeae-maydis*= Cz-mCRP1 (KAF2214354.1), CERZMDRAFT69800 (KAF2209663.1), Cz-mPHL1 (ACB38886.1); (4) *Arabidopsis thaliana*= AtPhototropin-2 (NP_568874.2); (5) *Zymoseptoria tritrici* predicted protein sequences were obtained from FungiDB= Zt_Wco1 (ZTRI_11.64), Zt_Vivid (ZTRI_9.516), ZtLov1 (ZTRI_2.1111), ZtLov2 (ZTRI_1.2082), ZtLov3 (ZTRI_4.300), Zt_Phy1 (ZTRI_5.141), Zt_Ops1 (ZTRI_5.210), Zt_Nop1 (ZTRI_13.368), Zt_Cry1 (ZTRI_1.86), Zt_Cry2 (ZTRI_13.203), Zt_Phl1 (ZTRI_1.976).

b Alignment of predicted LOV domain amino acid sequences in the white collar 1 homologue Zt_Wco1 from *Z. tritici* (FungiDB ID: ZTRI_11.64) and 3 uncharacterised putative light-dependent *Z. tritici* genes, consistently up- (Zt_Lov1, ZTRI_2.1111; Zt_Lov3, ZTRI_4.300) or down-regulated (Zt_Lov2, ZTRI_1.2082) in hyphae in 3-4 wildtype strains. Note that almost all chromophore-interacting residues and highly conserved LOV-domain residues[2] are present. The alignment was generated with Clustal Omega 1.2.4.

c Alignment of the predicted LOV domain amino acid sequences in the putative protein Zt_Lov2 (FungiDB ID: ZTRI_1.2082) and *Arabidopsis thaliana* phototropin-2 (NCBI ID: NP_568874.2). Both sequences share identity/similarity. The alignment was generated with Clustal Omega 1.2.4.

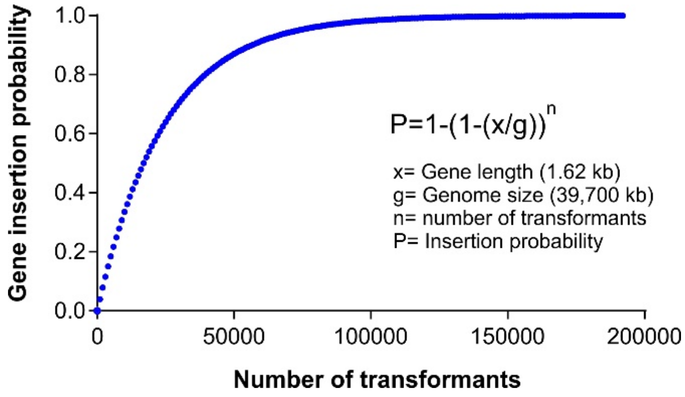

Supplementary Figure 8 Estimation of ATMT gene insertion probability in *Z. tritici.* The probability of insertion of G418 resistance cassette in each gene in the *Z. tritici* genome was calculated according to published procedures[3]. Source data are provided as a Source Data file.

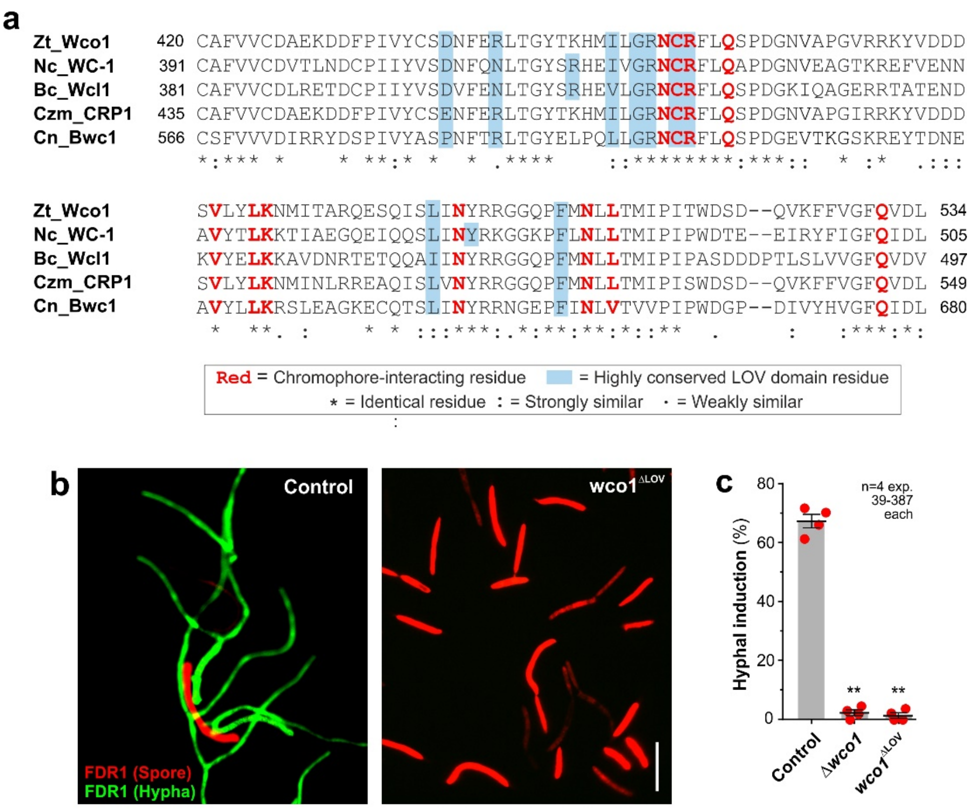

Supplementary Figure 9 Characterisation of the LOV domain in Wco1 from *Z. tritici*.

a Alignment of LOV domain sequences in the white collar homologues Zt_Wco1 from *Z. tritici* (FungiDB ID: ZTRI_11.64), Nc_WC1 from *N. crassa* (FungiDB ID:

NCU02356), BcWcl1 from *B. cinerea* (FungiDB ID: Bcin02g07400), Czm_CRP1 from *C. zeae-maydis* (NCBI ID: AEH41590.1) and Cn_Bwc1 from *C. neoformans* (FungiDB ID: CNL04450). Note that all reported chromophore-interacting residues[2] are conserved in Zt_Wco1. The alignment was generated in CLUSTAL Omega 1.2.4.

b Expression of FDR1 fluorescent hyphal (green) and spore (red) markers proteins in IPO323 wildtype cells (IPO323) and mutant cells that express a “light-blind” Wco1 protein, deleted in its LOV domain (IPO323Wco1$^{\Delta LOV}$) after growth for 2d in MM, 18ºC in darkness. Under these conditions, *Z. tritici* wildtype spores undergo dimorphic switching and form ZtGFP-expressing hyphae (left panel, green). Mutants that express the *wco1*$^{\Delta LOV}$ allele do not switch under these conditions, suggesting that the LOV domain of white collar 1 participates in light sensing. Scale bar= 20 µm.

c Hyphal programme induction in IPO323 (Control), white collar 1 deletion mutant (Δ*wco1*) and light-blind white collar 1 mutant (*wco1*$^{\Delta LOV}$) after 2d in MM, 18ºC, in darkness. In the absence of the WCC or the light-detection ability of Wco1 dimorphic switching is not induced.

Results shown in (**b**) were obtained independently in 4 experiments. Bars in (**c**) represent mean±SEM, sample size *n* given in graph; red dots represent individual experiments. Comparison with Control in (**c**) used Student’s t-testing with Welch correction; the sample size *n* is indicated; **= significant difference at two-tailed P= 0.0025 (Control vs Δwco1) and P= 0.0024 (Control vs wco1$^{\Delta LOV}$). Source data are provided as a Source Data file.

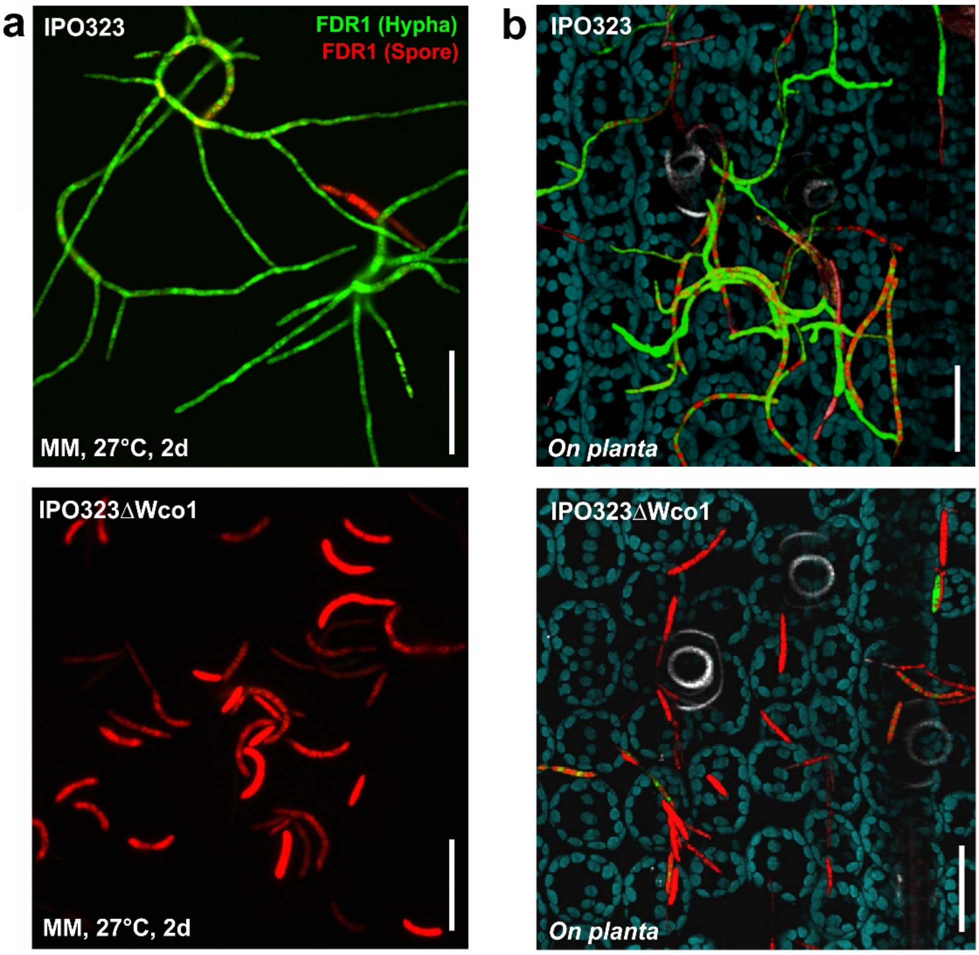

Supplementary Figure 10 Dimorphic switching of IPO323 wildtype and Δ*wco1* mutants.

The control strain IPO323_FDR1 shifts from spores (containing mCherry, red) to hyphae (containing eGFP, green) after 2 days in MM at 27ºC (**a**) and on the leaf surface (**b**), whereas the *wco1* null mutant strain IPO323_FDR1_ΔWco1 is strongly impaired in the spore-hypha transition. Results shown in (**a**, **b**) where obtained independently in 2 experiments. Scale bars= 30 µm. Results shown in (**a**, **b**) were obtained independently in 2 experiments. Source data are provided as a Source Data file.

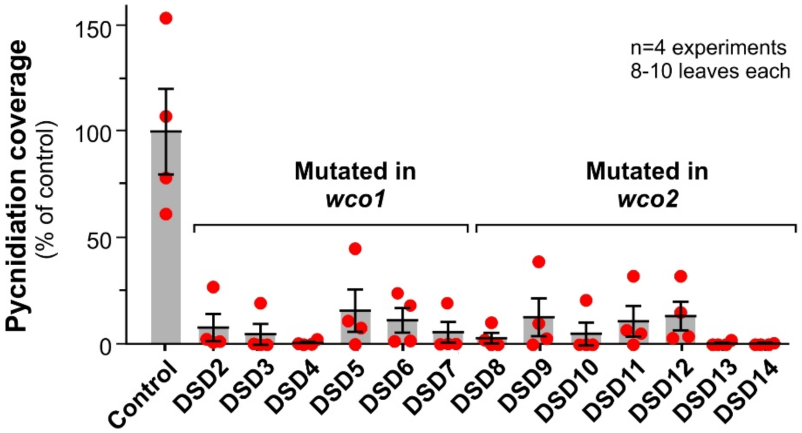

Supplementary Figure 11 Pathogenicity of DSD mutants with insertions in *wco1* and *wco2*. Bars represent mean±SEM, sample size *n* given in graph; red dots represent individual experiments. Control represents IPO323 wildtype.

Source data are provided as a Source Data file.

Supplementary Tables

Supplementary Table 1 *Z. tritici* strains used in this study.

**Name**	**Genotype**	**Source**
IPO323	Wildtype, *MAT1-1*	4
IPO323_ZtGSpa2_mChSso1	*MAT1-1* /pCZtGFPSpa2 / pHmCherrySso1, *cbx*^{R}, *hyg*^{R}	This study
IPO323_eGSso1	*MAT1-1*/pCeGFPSso1, *cbx*^{R}	5
IPO323_eG	*MAT1-1*/pCeGFP, *cbx*^{R}	6
IPO323_P*dnaJ*ZtG	*MAT1-1*/pCP*dnaJ*-ZtGFP, *cbx*^{R}	This study
IPO323_P*hsp60*ZtG	*MAT1-1*/pCP*hsp60*-ZtGFP, *cbx*^{R}	This study
IPO323_P*hsp70*/*ssa3*ZtG	*MAT1-1*/pCP*hsp70*/*ssa3*-ZtGFP, *cbx*^{R}	This study
IPO323_P*sti1*ZtG	*MAT1-1*/pCP*sti1*-ZtGFP, *cbx*^{R}	This study
IPO323_P*cdc37*ZtG	*MAT1-1*/pCP*cdc37*-ZtGFP, *cbx*^{R}	This study
IPO323_P*hsp70*/*bip1*ZtG	*MAT1-1*/pCP*hsp70*/*bip1*-ZtGFP, *cbx*^{R}	This study
IPO323_P*cne1*ZtG	*MAT1-1*/pCP*cne1*-ZtGFP, *cbx*^{R}	This study
IPO323_P*hsp70/ssc70*ZtG	*MAT1-1*/pCP*hsp70/ssc70*-ZtGFP, *cbx*^{R}	This study
IPO323_FDR1	*MAT1-1*/pCFDR1, *cbx*^{R}	This study
MC2306	Unknown	This study
1E4	Unknown	7
T5	Unknown	This study
MC2306_FDR1	Unknown/pCFDR1, *cbx*^{R}	This study
1E4_FDR1	Unknown/pCFDR1, *cbx*^{R}	This study
T5_FDR1	Unknown/pCFDR1, *cbx*^{R}	This study
IPO323_eGSso1_3LysMmCh	*MAT1-1*, P*3lysm-3lysm-mCherry*/pCeGFPSso1, *cbx*^{R}, *hyg*^{R}	This study
IPO323_eGSso1_1LysMmCh	*MAT1-1*, P*1lysm-1lysm-mCherry*/pCeGFPSso1, *cbx*^{R}, *hyg*^{R}	This study
IPO323_mChSso1_P*3lysm*ZtG	*MAT1-1*/pHmCherrySso1/pCP*3lysm*-ZtGFP, *hyg*^{R}, *cbx*^{R}	This study
IPO323_mChSso1_P*nis1*ZtG	*MAT1-1*/pHmCherrySso1/pCP*nis1*-ZtGFP, *hyg*^{R}, *cbx*^{R}	This study
IPO323_mChSso1_P*nip1*ZtG	*MAT1-1*/pHmCherrySso1/pCP*nip1*-ZtGFP, *hyg*^{R}, *cbx*^{R}	This study
IPO323_mChSso1_P*1lysm*ZtG	*MAT1-1*/pHmCherrySso1/pCP*1lysm*-ZtGFP, *hyg*^{R}, *cbx*^{R}	This study
IPO323_mChSso1_P*ecp2.2*ZtG	*MAT1-1*/pHmCherrySso1/pCP*ecp2.2*-ZtGFP, *hyg*^{R}, *cbx*^{R}	This study
IPO323_FDR1_DSD1-16	*MAT1-1*/pCFDR1/pCGen, *cbx*^{R}, *G418*^{R}	This study
IPO323_FDR1_ΔWco1	*MAT1-1*/pCFDR1, Δ*wco1*, *cbx*^{R}, *hyg*^{R}	This study
IPO323_FDR1_Wco1$^{\Delta LOV}$	*MAT1-1*/pCFDR1, *wco1*$^{\Delta 420\text{-}534}$, *cbx*^{R}, *hyg*^{R}	This study
IPO323_FDR1_DSD1_Wco1	*MAT1-1*, Δ*wco1* /pCFDR1/pCGen, *cbx*^{R}, *G418*^{R}, *hyg*^{R}	This study
MC2306_FDR1_ΔWco1	Unknown/pCFDR1, Δ*wco1*, *cbx*^{R}, *hyg*^{R}	This study
1E4_FDR1_ΔWco1	Unknown/pCFDR1, Δ*wco1*, *cbx*^{R}, *hyg*^{R}	This study
T5_FDR1_ΔWco1	Unknown/pCFDR1, Δ*wco1*, *cbx*^{R}, *hyg*^{R}	This study
IPO323_ΔWco1	*MAT1-1*, Δ*wco1*, *hyg*^{R}	This study
MC2306_ΔWco1	Unknown, Δ*wco1*, *hyg*^{R}	This study
1E4_ΔWco1	Unknown, Δ*wco1*, *hyg*^{R}	This study
T5_ΔWco1	Unknown, Δ*wco1*, *hyg*^{R}	This study
IPO323_mChSso1_P*3lysm*ZtG_ΔWco1	*MAT1-1*, Δ*wco1*/pHmCherrySso1/pCP*3lysm*ZtGFP, *hyg*^{R}, *cbx*^{R}, *G418*^{R}	This study
IPO323_mChSso1_P*1lysm*ZtG_ΔWco1	*MAT1-1*, Δ*wco1*/pHmCherrySso1/pCP*1lysm*ZtGFP, *hyg*^{R}, *cbx*^{R}, *G418*^{R}	This study

-: fusion; /: ectopically integrated; eG, eGFP or *egfp*: enhanced green fluorescent protein; p: plasmid; Sso1: a syntaxin-like plasma membrane protein; Spa2: a polarity protein; mCh or mCherry: monomeric red-fluorescent mCherry protein; *hsp*: heat shock protein; *cdc37*: gene for a chaperone cell division cycle 37; *sti1*: gene for a co-chaperone stress-inducible protein 1; *cne1*: gene for a chaperone calnexin; FDR1: a fluorescent dimorphic reporter plasmid, expressing ZtGFP and mCherry under the control of promoters of the hypha-induced gene ZTRI_13.167 and the spore-induced gene ZTRI_7.69 (FungiDB gene IDs); *1lysm* and *3lysm*: lysin domain-containing effectors in *Z. tritici*; P: promoter; ZtG or ZtGFP: codon-optimised green-fluorescent protein; H or *hyg*^{R}: hygromycin resistance; G or *G418*^{R}: geneticin resistance; C or *cbx*^{R}, carboxin resistance; *tub2*: gene for α-tubulin; *nis1*: gene for homologue of the necrosis factor Nis1; *nip1:* gene for the necrosis factor Nip1; *ecp2.2*: gene for a putative extracellular protein 2-like effector; *gpdI*, glycerol-3-phosphate dehydrogenase; *nptII*, neomycin phosphotransferase gene; DSD: dimorphic switch defective mutants; Δ: deletion; *wco1*; a homologue of the *N. crassa* white collar 1 protein; LOV: light/oxygen/voltage sensitive domain.

Supplementary Table 2 Plasmids used in this study.

Name	Description	Source
pCZtGFPSpa2	P*tub2-ztgfp-spa2*, *cbx*^{R}	8
pHmCherrySso1	P*tub2-mCherry-sso1*, *hyg*^{R}	5
pCeGFPSso1	P*tub2-egfp-sso1*, *cbx*^{R}	5
pCeGFP	P*tub2-egfp*, *cbx*^{R}	6
pCP*dnaJ*-ZtGFP	P*dnaJ-ztgfp*, *cbx*^{R}	This study
pCP*hsp60*-ZtGFP	P*hsp60-ztgfp*, *cbx*^{R}	This study
pCP*hsp70*/*ssa3*-ZtGFP	P*hsp70*/*ssa3-ztgfp*, *cbx*^{R}	This study
pCP*sti1*-ZtGFP	P*sti1-ztgfp*, *cbx*^{R}	This study
pCP*cdc37*-ZtGFP	P*cdc37-ztgfp*, *cbx*^{R}	This study
pCP*hsp70*/*bip1*-ZtGFP	P*hsp70*/*bip1-ztgfp*, *cbx*^{R}	This study
pCP*cne1*-ZtGFP	P*cne1-ztgfp cbx*^{R}	This study
Pcp*hsp70/ssc70*-ZtGFP	P*hsp70/ssc70-ztgfp*, *cbx*^{R}	This study
pCP*ztri13.167*ZtGFP	P*ztri13*.167-*ztgfp*, *cbx*^{R}	This study
PCP*ztri7.69*mCherry	P*ztri7.69-mCherry*, *cbx*^{R}	This study
pCFDR1	P*ztri13.167-ztgfp*, P*ztri7.69-mCherry*, *cbx*^{R}	This study
pH3LysMmCherry	P*3lysm-3lysm-mCherry*, *hyg*^{R}	This study
pH1LysMmCherry	P*1lysm-1lysm-mCherry*, *hyg*^{R}	This study
pCP*3lysm*ZtGFP	P*3lysm-ztgfp*, *cbx*^{R}	This study
pCP*nis1*ZtGFP	P*nis1-ztgfp*, *cbx*^{R}	This study
pCP*nip1*-ZtGFP	P*nip1-ztgfp*, *cbx*^{R}	This study
pCP*1lysm*ZtGFP	P*1lysm-ztgfp*, *cbx*^{R}	This study
pCP*ecp2.2*ZtGFP	P*ecp2.2-ztgfp*, *cbx*^{R}	This study
pCGen	P*gpdI-nptII*, *G418*^{R}	9
pHΔWco1	*Δwco1*, *hyg*^{R}	This study
pHWco1$^{\Delta LOV}$	*wco1* $^{\Delta 420\text{-}534}$, *hyg*^{R}	This study
pHWco1	P*wco1-wco1*, *hyg*^{R}	This study
pGΔWco1	*Δwco1*, *G418*^{R}	This study

p: plasmid; Sso1: a syntaxin-like plasma membrane protein; Spa2: a polarity protein; mCherry: monomeric red-fluorescent mCherry protein; *hsp*: heat shock protein; *cdc37*: gene for a chaperone cell division cycle 37; *sti1*: gene for a co-chaperone stress-inducible protein 1; *cne1*: gene for a chaperone calnexin; FDR1: a fluorescent dimorphic reporter plasmid, expressing ZtGFP and mCherry under the control of promoters of the hypha-induced gene ZTRI_13.167 and the spore-induced gene ZTRI_7.69 (FungiDB gene IDs); *1lysm* and *3lysm*: lysin domain-containing effectors in *Z. tritici*; P: promoter; ZtG or ZtGFP: codon-optimised green-fluorescent protein; H or *hyg*^{R}: hygromycin resistance; G or *G418*^{R}: geneticin resistance; C or *cbx*^{R}, carboxin resistance; *tub2*: gene for α-tubulin; *nis1*: gene for homologue of the necrosis factor Nis1; *nip1:* gene for the necrosis factor Nip1; *ecp2.2*: gene for a putative extracellular protein *2*-like effector; *gpdI*, glycerol-3-phosphate dehydrogenase; *nptII*, neomycin phosphotransferase gene; DSD: dimorphic switch defective mutants; Δ: deletion; *wco1*; a homologue of the *N. crassa* white collar 1 protein; LOV: light/oxygen/voltage sensitive domain.

Supplementary Table 3 Experimental usage of *Z. tritici* strains.

Strain name	Figure or Movie
IPO323	Figs. 1a-c, 4a-c, 5a, 5b; 8c, 9d; Supp. Figs. 1a, 6, 6; Movie S2
PO323_ZtGSpa2_mChSso1	Fig. 1c
IPO323_eGSso1	Supp. Figs. 1b-d, 4; Movie S1
IPO323_eG	Supp. Figs. 1e; Figs 9c, 9d; Movie S3
IPO323_P*dnaJ*ZtG	Figs. 1d, 1e
IPO323_P*hsp60*ZtG	Fig. 1e
IPO323_P*hsp70*/*ssa3*ZtG	Fig. 1e
IPO323_P*sti1*ZtG	Supp. Fig. 2a
IPO323_P*cdc37*ZtG	Supp. Fig. 2a
IPO323_P*hsp70*/*bip1*ZtG	Supp. Fig. 2a
IPO323_P*cne1*ZtG	Supp. Fig. 2a
IPO323_P*hsp70*/*ssc70* ZtG	Supp. Fig. 2a
IPO323_FDR1	Figs. 2b-f, 2h, 7a, 7b, 7e-g; Supp. Figs. 3a, 3b, 9b, 9c, 10a, 10b
MC2306	Figs. 3a, 4a-c, 8c; Supp. Fig. 6
1E4	Figs. 3a, 4a-c, 8c; Supp. Fig. 6
T5	Figs. 3a, 4a-c, 8c;
MC2306_FDR1	Figs. 3b, 3c, 8b
1E4_FDR1	Figs. 3b, 3c, 8b
T5_FDR1	Figs. 3b, 3c, 8b
IPO323_eGSso1_3LysMmCh	Fig. 5c
IPO323_eGSso1_1LysMmCh	Fig. 5c
IPO323_mChSso1_P*3lysm*ZtG	Figs. 6a, 6b; Movie S4
IPO323_mChSso1_P*nis1*ZtG	Figs. 6a, 6b; Movie S5
IPO323_mChSso1_P*nip1*ZtG	Figs. 6a, 6b; Movie S6
IPO323_mChSso1_P*1lysm*ZtG	Fig. 6b
IPO323_mChSso1_P*ecp2.2*ZtG	Fig. 6b
IPO323_FDR1_DSD1	Figs. 7a-c, 7f, 7g
IPO323_FDR1_DSD2 to DSD7	Figs. 7a, 7c; Supp. Fig. 11
IPO323_FDR1_DSD8 to DSD14	Figs. 7a, 7d; Supp. Fig. 11
IPO323_FDR1_DSD15 to DSD16	Fig. 7a
IPO323_FDR1_ΔWco1	Figs. 7e-g; Supp. Figs. 9c, 10a, 10b
IPO323_FDR1_Wco1$^{\Delta LOV}$	Figs. 7e-g; Supp. Figs. 9b, 9c
IPO323_FDR1_DSD1_Wco1	Figs. 7f, 7g
MC2306_FDR1_ΔWco1	Fig. 8a, 8b
1E4_FDR1_ΔWco1	Figs. 8a, 8b
T5_FDR1_ΔWco1	Figs. 8a, 8b
IPO323_ΔWco1	Fig. 8c
MC2306_ΔWco1	Fig. 8c
1E4_ΔWco1	Fig. 8c
T5_ΔWco1	Fig. 8c
IPO323_mChSso1_P*1lysm*ZtG_ΔWco1	Fig. 8d
IPO323_mChSso1_P*3lysm*ZtG_ΔWco1	Fig. 8d

Supplementary Table 4 Putative *Z. tritici* heat stress proteins tested in this study.

Name	Gene ID[a]	*A. fumigatus* gene[b]	BLAST[c]	Function
DnaJ	ZTRI_8.716	AFUA_5G07340	2e-57	Chaperone
Hsp60	ZTRI_8.689	AFUA_2G09290	0.00	Chaperonin
Hsp70/Ssa3	ZTRI_10.182	AFUA_1G07440	0.00	Chaperone
Sti1	ZTRI_1.122	AFUA_7G01860	0.00	Chaperone regulator
Cdc37	ZTRI_3.300	AFUA_4G10010	1e-175	Chaperone
Hsp70/Bip1	ZTRI_1.1084	AFUA_2G04620	0.00	Chaperone
Calnexin/Cne1	ZTRI_4.802	AFUA_4G12850	0.00	Chaperone
Hsp70/Ssc70	ZTRI_5.654	AFUA_2G09960	0.00	Chaperone

[a]Accessible at FungiDB (https://fungidb.org/fungidb/app/record/dataset/NCBITAXON_3367220

[b]Shown to upregulated in heat-treated *A. fumigatus* cells (48ºC, ref. 10)

[c]Error probability for NCBI BLASTp (https://blast.ncbi.nlm.nih.gov/Blast.cgi?PAGE=Proteins)

Supplementary Table 5 Published dimorphism genes in *Z. tritici*.

Name	Gene ID[a] (protein type)	Phenotype	Source
A) Transcription factors			
Ste12	ZTRI_8.206 (STE-like)	Deletion causes reduced hyphal growth; reduced pathogenicity	11
ZtVelB	ZTRI_7.41 (Velvet protein)	Deletion causes absence of yeast-like spores; no effect on pathogenicity	12
ZtWor1	ZTRI_8.107 (Gti1/Pac2)	Deletion results in much less "yeast-like" spores; reduced pathogenicity	13
AlmA	ZTRI_12.436 (Zn(II)$_2$Cys$_6$)	Less hyphae when overexpressed	14
ZtVf1	ZTRI_7.203 (C2H2-zinc fing.)	Deletion causes shorter hyphae and reduced branching; reduced pathogenicity	15
Zt107320	ZTRI_1.1162 (Zn(II)$_2$Cys$_6$)	Deletion causes more hyphae and more branching; reduced pathogenicity	16
Mve1	ZTRI_2.1013 (Velvet protein)	Deletion causes a reduction in hyphal growth; no effect on pathogenicity	17
myco#56	ZTRI_8.455 (Regulator Cip2)	ATMT mutant shows abnormal and shorter hyphal growth	18
B) Signalling			
Ste11p	ZTRI_4.424 (MAPKKK)	Deletion abolishes hyphal growth, with most cells remaining yeast-like; non-pathogenic	11
Ste7p	ZTRI_1.496 (MAPKK)	Deletion abolishes hyphal growth, with most cells remaining yeast-like; non-pathogenic	11
MgSte50	ZTRI_2.972 (Ste11p-regulator)	Reduced hyphal growth; reduced pathogenicity	11
MgFus3	ZTRI_6.302 (MAPK)	Deletion impairs later hyphal growth; non-pathogenic	19
MgHog1	ZTRI_10.412 (MAPK)	Deletion affects hyphal length; null mutant non-pathogenic	18,20
MgSlt2	ZTRI_1.392 (MAPK)	Deletion impairs hyphal growth and morphology of spores; null mutant reduced pathogenic	21
ZtSsk1	ZTRI_3.792 (Osmo-sensor)	ATMT mutant shows impaired radial growth; (short hyphae?); null mutant non-pathogenic	18
MgTpk2	ZTRI_3.1002 (Prot. Kin. A, cat.)	Deletion results in shorter hyphae and reduced pathogenicity	22
MgBcy1	ZTRI_1.532 (Prot. Kin. A,reg.)	Deletion results in shorter hyphae and reduced pathogenicity	22
MgGpa1	ZTRI_1.856 (G-Protein, α)	Longer spores; no obvious effect on hyphal growth; reduced pathogenicity	23
MgGpa3	ZTRI_10.413 (G-Protein, α)	Deletion impairs slightly hyphal growth; reduced pathogenicity	23
MgGpb1	ZTRI_2.957 (G-Protein, β)	Deletion results in shorter hyphae with aberrant morphology; reduced pathogenicity	23
C) Miscellaneous			
myco5#5	ZTRI_1.1551 (Vps37)	Deletion induces hyphal growth and represses yeast-like spores, non-pathogenicity	24
myco#100	ZTRI_6.227 (Phospholipase A)	unclear; in paper same picture provided for myco#100, myco#26 and myco#552	18
myco#76	ZTRI_12.391 (unknown)	ATMT mutant shows little radial growth; suggestive of very short hyphae	18
myco#4	ZTRI_8.17 (Purine synth.)	ATMT mutant shows much less hyphae; null mutant non-pathogenic	18
myco#3	ZTRI_11.452 (LETM1-like)	ATMT mutant shows abnormal radial growth, (non-directed and shorter hyphae?)	18
ZtGT2	ZTRI_1.565 (Glycosyltransf.)	Null mutant formed shorter and irregular hyphae; null mutant non-pathogenic	25
MgAlg2	ZTRI_9.53 (Mannosyltransf.)	ATMT mutant is not able to form hyphae; mutant is non-pathogenic	9
Mcc1	ZTRI_9.474 (Cyclin 1)	Deletion causes slow hyphal growth, hyphae show morphological defects; reduced pathogenicity	26

[a] FungiDB at https://fungidb.org/fungidb/app.

Supplementary Table 6 Transcription factors with roles in fungal development.

Gene ID	Name[a]	Organism	Proposed function[b]	Source
ZTRI_2.1013	BcVEL1 (3e-89)<br>AnVeA (4e-78)	*Botrytis cinerea*<br>*Aspergillus. nidulans*	Inhibitor of conidiation<br>Fruiting body development	27, 28
ZTRI_7.41	BcVEL2 (2e-68)<br>AnVelB (4e-37)	*Botrytis cinerea*<br>*Aspergillus nidulans*	Inhibitor of conidiation<br>Fruiting body development	28, 29
ZTRI_3.788	BcVEL3 (4e-35)<br>AnVosA (2e-40)	*Botrytis cinerea,*<br>*Aspergillus. nidulans*	Not expressed in *B. cinerea*<br>Fruiting body development	27,28
ZTRI_3.461	BcVEL4 (1e-33)<br>AnVosA (7e-22)	*Botrytis cinerea*<br>*Aspergillus nidulans*	Not expressed[c]<br>Fruiting body development	28,30
ZTRI_3.864	BcLAE1 (3e-52)<br>AnLea1 (6e-93)	*Botrytis cinerea*<br>*Aspergillus nidulans*	Inhibitor of conidiation[c]	27, 28
ZTRI_1.1139	NcFlb3 (4e-59)	*Neurospora crassa*	Controls hyphal morphology	31
ZTRI_4.735	Fluffy (9e-10)	*Neurospora crassa*	Control of hyphal to budding (conidiation) growth transition	32
ZTRI_1.1143	Acon-2 (7e-76)	*Neurospora crassa*	Control of hyphal to budding (conidiation) growth transition	33
ZTRI_10.332	Acon-3 (8e-79)	*Neurospora crassa*	Control of hyphal to budding (conidiation) growth transition	33
ZTRI_9.516	ENVOY (2e-39)	*Trichoderma reesei*	Fruiting body formation	34
ZTRI_7.76	RosA (1e-143)<br>NosA (0.0)	*Aspergillus nidulans*<br>*Aspergillus nidulans*	Fruiting body formation	35, 36
ZTRI_12.338	BcCrz1 (7e-116)	*Botrytis cinerea*	Required for hyphal growth	37
ZTRI_2.426	BcHOX8 (0.0)	*Botrytis cinerea*	Repressor of hyphal growth	38
ZTRI_1.516	BcATF1 (2e-47)	*Botrytis cinerea*	Inhibitor of hyphal growth	39
ZTRI_1.381	AfWetA (4e-19)	*Aspergillus fumigatus*	Spore formation	40
ZTRI_4.233	AfAbaA (2e-28)	*Aspergillus fumigatus*	Spore formation	40
ZTRI_8.107	BcREG1 (2e-57)	*Botrytis cinerea*	Controls conidiation	41
ZTRI_8.378	BcLTF2 (2e-71)	*Botrytis cinerea*	Inhibitor of hyphal growth	42
ZTRI_1.1757	BcLTF1 (4e-40)	*Botrytis cinerea*	Inhibitor of conidiation	43
ZTRI_1.824	BcLTF3 (5e-11)	*Botrytis cinerea*	Controls conidiation	44
ZTRI_2.187	FoCon7 (9e-40)	*Fusarium oxysporum*	Conidiation, morphogenesis	45
ZTRI_10.234	MoSwi6 (5e-176)	*Magnaporthe oryzae*	Inhibitor of conidiation	46
ZTRI_8.380	Tup1 (5e-151)	*Candida albicans*	Inhibitor of hyphal growth	47
ZTRI_2.479	Znc1 (5e-11)	*Yarrowia lipolytica*	Inhibitor of hyphal growth	48
ZTRI_1.868.1	AfRgdA (3e-143)	*Aspergillus fumigatus*	Inhibitor of sporulation	49
ZTRI_10.333	SpRrn7 (3e-33)	*Schizosaccharom. pombe*	Core TF for ribosomal proteins	50
ZTRI_5.521	AnBrlA (5e-12)	*Aspergillus nidulans*	Inducer of sporulation	51
ZTRI_6.274	StuA (7e-168)	*Leptosphaeria maculans*	Spore formation	52, 53

[a] pBLAST error probability in brackets.
[b] Not all proposed cellular roles are included.
[c]Involved in light-regulated morphology control.

Supplementary Table 7 Bioinformatic tools used in this study

Programme	Use	Description	Web address	Source
**Bedtools 2.26.0**	Functional annotation	Determine concordance between differing gene annotations	bedtools.readthedocs.io/en/latest	54
**Blast2GO 5.0**	Functional annotation	Functional predictions for protein sequences based on blast searches	blast2go.com	55
**Pfam 32.0**	Functional annotation	Functional domain prediction	pfam.xfam.org	56
**SignalP 5.0**	Functional annotation	Fungal secretome prediction	cbs.dtu.dk/services/SignalP	57
**EffectorP 2.0**	Functional annotation	Fungal effector prediction	effectorp.csiro.au	58
**FungiDB Release 46**	Functional annotation	Incorporation of functional annotations	fungidb.org	59
**TMHMM 2.0**	Functional annotation	Transmembrane domain prediction	cbs.dtu.dk/services/TMHMM	60
**Phobius 1.01**	Functional annotation	Prediction of Transmembrane domain prediction	phobius.sbc.su.se	61
**EMBOSS Needle**	Functional annotation	Sequence comparison	ebi.ac.uk/Tools/psa/emboss_needle	62
**ClustalOmega**	Functional annotation	Sequence comparison	ebi.ac.uk/Tools/msa/clustalo	63
**Cutadapt 1.13**	Sequencing data quality	Removal of sequencing adapter sequences and low quality data	cutadapt.readthedocs.io/en/stable/guide.html	64
**FastQC 0.11.4**	Sequencing data quality	Sequencing data quality assessment	www.bioinformatics.babraham.ac.uk/projects/fastqc	none
**FastQScreen 0.5.2**	Sequencing data quality	DNA/RNA contamination screening	bioinformatics.babraham.ac.uk/projects/fastq_screen	65
**TopHat2 2.1.1**	RNAseq analysis	RNA sequence data alignment	ccb.jhu.edu/software/tophat/index.shtml	66
**HTSeq-count 0.10.0**	RNAseq analyssi	Quantify the reads aligned to each gene	htseq.readthedocs.io/en/release_0.11.1/count.html	67
**DESeq2 1.14.1**	RNAseq analysis	Differential expression and Principle component analysis	bioconductor.org/packages/devel/bioc/vignettes/DESeq2/inst/doc/DESeq2.html	68
**JASPAR release 8**	WC-binding motif	Download the *N. crassa* Wco1 binding motif	jaspar.genereg.net/matrix/MA1437.1/	69
**FIMO 5.1.0**	WC-binding motif	Scan 1kb promotor regions for Wco1 binding motifs	meme-suite.org/doc/fimo.html	70
**BWA-mem 0.7.15**	Genome sequencing	DNA sequence data alignment	github.com/lh3/bwa	71
**Picard 2.12.1**	Genome sequencing	SAM to BAM conversion; Fix mate pair information; Mark duplicate reads	broadinstitute.github.io/picard/	none
**Samtools 1.3.1**	DSD mutant analysis	Detection of G418 cassette insertion site	htslib.org	72
**Seqtk 1.2**	DSD mutant analysis	Detection of G418 cassette insertion site	github.com/lh3/seqtk	none
**MEGA5.2**	Phylogenetic tree	Comparison of photo-receptors	https://www.megasoftware.net	1
**IGV 2.4.10**	DSD mutant analysis	Visualisation of G418 cassette insertion site	software.broadinstitute.org/software/igv	73

Supplementary Table 8 Primers used in this study.

Primer	Sequence (5’ to 3’)
CA86	*CATTTGCGGCTGTCTCGAAATCGACGGAAG*CCACGATTTCCTGGGACTACTGC
CA87	*GGTGAAGAGCTCCTCGCCCTTGGAGACCATTTTGATTGAAGTTGAAGTTGAAGTTGAA*
CA98	*ACCGGCGGCATGGACGAGCTGTACAAGTAA*CGTTCAAACATTTGGCAATAAAGTTTC
CA99	*GGGAACAGACTTGAGTATGTTGTAAAATGT*CCCGATCTAGTAACATAGATGACAC
CA102	*CATTTGCGGCTGTCTCGAAATCGACGGAAG*ATGCTATCCTCTTTGCCGCATGTC
CA103	*CATGTTATCCTCCTCGCCCTTGCTCACCAT*GTGGCGGATGAAGAAAGAAAAAGGA
CA115	ACATTTTACAACATACTCAAGTCTGTT
CA116	GTTGAAGTTCTGCGTCGGATCCG
CA117	*CTCTCATAAGAGCTTGGCTGTCGACTCCTC*CGATGAATTCTCATGTTTGACAGCTT
CA120	*GGGAACAGACTTGAGTATGTTGTAAAATGT*ATGCTATCCTCTTTGCCGCATG
SK19	GAGGAGTCGACAGCCAAGCTC
SK46	GAAGTCTGCGGCAGCTCGCAC
SK136	CCCAACTGATATTGAAGGAGCATT
SK137	CCCGATCTAGTAACATAGATGACA
SK208	*TGGCAGGATATATTGTGGTGTAAACAAATT*TCGCCAAGCAGTACAACTCGGG
SK210	*CATGTTATCCTCCTCGCCCTTGCTCACCATG*GCAGACGCAGTGATGTTGTGG
SK211	*GCGCGGTGTCATCTATGTTACTAGATCGGGG*AGGTGGACAGTGTGAACGCCG
SK212	*TAAACGCTCTTTTCTCTTAGGTTTACCCGCG*ATCCCGTCGACGTCGTACGTC
SK214	*CCAAAAAATGCTCCTTCAATATCAGTTGGGC*GATGAATTCTCATGTTTGACAGC
SK215	ATGGTGAGCAAGGGCGAGGAG
SK297	GCGACGACGGACGAGGACAGG
SK302	*TGGCAGGATATATTGTGGTGTAAACAAATTT*GAGTACCAGCACAGCAGGTCTC
SK303	*CATGTTATCCTCCTCGCCCTTGCTCACCATG*AGGCAGCTGTTGCGGTCGTAG
SK304	*GCGCGGTGTCATCTATGTTACTAGATCGGGT*CTCGAAACTTCGGGATGTGGTTG
SK305	*TAAACGCTCTTTTCTCTTAGGTTTACCCGCT*TCAGCGTGCTTTGATGTCGTCG
SK314	*CATTTGCGGCTGTCTCGAAATCGACGGAAGC*CACGACACACGACTGTACAAGA
SK315	*GGTGAAGAGCTCCTCGCCCTTGGAGACCAT*TTTTGCGGTTGGCTTTGATCGAGA
SK316	*CATTTGCGGCTGTCTCGAAATCGACGGAAGT*GACGGCTGCCAGGAAGCCATC
SK317	*GGTGAAGAGCTCCTCGCCCTTGGAGACCAT*GTTGACGGATGCGTGTTGGGAG
SK573	*CATTTGCGGCTGTCTCGAAATCGACGGAAGC*TGGACTACTACAGCCGACAAAC
SK574	*GGTGAAGAGCTCCTCGCCCTTGGAGACCAT*GTTGAGAGAAAAGAGCGAGAGAGT
SK577	*CATTTGCGGCTGTCTCGAAATCGACGGAAG*CCACGATTTCCTGGGACTACTGC
SK578	*GGTGAAGAGCTCCTCGCCCTTGGAGACCAT*TTTGATTGAAGTTGAAGTTGAAGTTGA
SK699	*TGGCAGGATATATTGTGGTGTAAACAAATT*AGTACCTCACCCAGGTAAAGCAC
SK700	*CCAAAAAATGCTCCTTCAATATCAGTTGGG*CGTGGACGGAACCCTCGGGTG
SK701	*GCGCGGTGTCATCTATGTTACTAGATCGGG*TTGATTGATTGAATTGGTCGGTTCTTG
SK702	*TAAACGCTCTTTTCTCTTAGGTTTACCCGC*GAAAGGAGTTCACAAGGTTTCCG
SK706	*GTAGTTTCCGGGATAGGTGTAGCCATTCAT*GGCGATGGTGGTATGCGGATG
SK707	ATGAATGGCTACACCTATCCCGG
SK713	*CAATATCAGTTGGGTACCGAGCTCGAATTC*GAAAGGAGTTCACAAGGTTTCCG
SK714	TGATTCTCGGGAGGAATTGCCGC
SK715	CCTCGGCGATAGTTGATCAGCG
SK772	*AGGGCCACCCACCCGAGGGTTCCGTCCACG*GCGGCTTCGAATCGTGGCTACC
SK773	*AACACAAGAACCGACCAATTCAATCAATCA*AATCATCATGCAACATGCATGTACTG
SK833	*CTTGTTGGTCACCGAACTCGGCTGCTCCAC*GGACAAATCGACGGATCCGATGT
SK834	*CAGATCAACATCGGATCCGTCGATTTGTCC*GTGGAGCAGCCGAGTTCGGTGA
SK840	*TGGCAGGATATATTGTGGTGTAAACAAATT*AGTACCTCACCCAGGTAAAGCAC
SK841	*CCAAAAAATGCTCCTTCAATATCAGTTGGG*ATATCCTTTTCCAATGCTCCCTGC
SK842	*GCGCGGTGTCATCTATGTTACTAGATCGGG*CTACGGTAACATGGCGATCGATAT
SK882	*CATTTGCGGCTGTCTCGAAATCGACGGAAG*CGATCCGGACCAGGAAGCTGAA
SK883	*GGTGAAGAGCTCCTCGCCCTTGGAGACCAT*TGTGGTGGTGGGTTGAGTGGATG
SK894	*CATTTGCGGCTGTCTCGAAATCGACGGAAG*TCCGAGCGATGATCAACTCGAAGA
SK895	*GGTGAAGAGCTCCTCGCCCTTGGAGACCAT*CGTGGCGGCGTGCTTGTGTGT
SK896	*CATTTGCGGCTGTCTCGAAATCGACGGAAG*GGAGGCCAGAGATATTGGGGAG
SK897	*GGTGAAGAGCTCCTCGCCCTTGGAGACCAT*GGTGAAGGTAGTTGTGTCGTAAGT
SK898	*CATTTGCGGCTGTCTCGAAATCGACGGAAG*ATTCAGCGATCTCAGAGCAACAAA
SK899	*GGTGAAGAGCTCCTCGCCCTTGGAGACCAT*CGCTGTGGAGTGATCTTGGTCT
SK902	*CATTTGCGGCTGTCTCGAAATCGACGGAAG*CAACTCGTCCATCAGATTCTTGAAG
SK903	*GGTGAAGAGCTCCTCGCCCTTGGAGACCAT*CTTTCACCAGAGGCCGGGTGCT
SK904	*CATTTGCGGCTGTCTCGAAATCGACGGAAG*AGCTGGACCACGGCCGCTGTG
SK905	*GGTGAAGAGCTCCTCGCCCTTGGAGACCAT*GATGGCGGTTTTAGCACACTGTG
SK906	*CATTTGCGGCTGTCTCGAAATCGACGGAAG*CAAGAAGGGTGAGGCCGACGTC
SK907	*GGTGAAGAGCTCCTCGCCCTTGGAGACCAT*GATTGTTTTGATCGATCAGTCAGTG
SK908	*CATTTGCGGCTGTCTCGAAATCGACGGAAG*ACTATTTTGCGGATGCCAGGCGAT
SK909	*GGTGAAGAGCTCCTCGCCCTTGGAGACCAT*GGTTGCGGTTGTTGGAGATGCC
SK912	*CATTTGCGGCTGTCTCGAAATCGACGGAAG*GCTGCGGCGAGGAAGAGGGAG
SK913	*GGTGAAGAGCTCCTCGCCCTTGGAGACCAT*GGTGGGCAGTCCTGGGAGTGT

Italics indicate sequence that is complementary with another DNA fragment, which allows homologous recombination in *S. cerevisiae*.

Supplementary References

1 Tamura, K. *et al.* MEGA5: molecular evolutionary genetics analysis using maximum likelihood, evolutionary distance, and maximum parsimony methods. *Mol. Biol. Evol.* **28**, 2731-2739 (2011).

2 Crosson, S., Rajagopal, S. & Moffat, K. The LOV domain family: photoresponsive signaling modules coupled to diverse output domains. *Biochemistry* **42**, 2-10 (2003).

3 Krysan, P. J., Young, J. C. & Sussman, M. R. T-DNA as an insertional mutagen in Arabidopsis. *The Plant Cell* **11**, 2283-2290 (1999).

4 Kema, G. H. & van Silfhout, C. H. Genetic variation for virulence and resistance in the wheat-*Mycosphaerella graminicola* pathosystem III. Comparative seedling and adult plant experiments. *Phytopathology* **87**, 266-272 (1997).

5 Kilaru, S., Schuster, M., Ma, W. & Steinberg, G. Fluorescent markers of various organelles in the wheat pathogen *Zymoseptoria tritici*. *Fungal Genet. Biol.* **105**, 16-27 (2017).

6 Kilaru, S. *et al.* A gene locus for targeted ectopic gene integration in *Zymoseptoria tritici*. *Fungal Genet. Biol.* **79**, 118-124 (2015).

7 Croll, D., Zala, M. & McDonald, B. A. Breakage-fusion-bridge cycles and large insertions contribute to the rapid evolution of accessory chromosomes in a fungal pathogen. *PLoSGenet.* **9**, e1003567 (2013).

8 Guo, M., Kilaru, S., Schuster, M., Latz, M. & Steinberg, G. Fluorescent markers for the Spitzenkörper and exocytosis in *Zymoseptoria tritici*. *Fungal Genet. Biol.* **79**, 158-165 (2015).

9 Motteram, J. *et al.* Aberrant protein N-glycosylation impacts upon infection-related growth transitions of the haploid plant-pathogenic fungus *Mycosphaerella graminicola*. *Mol. Microbiol.* **81**, 415-433 (2011).

10 Albrecht, D., Guthke, R., Brakhage, A. A. & Kniemeyer, O. Integrative analysis of the heat shock response in *Aspergillus fumigatus*. *BMC Genomics* **11**, 32 (2010).

11 Kramer, B., Thines, E. & Foster, A. J. MAP kinase signalling pathway components and targets conserved between the distantly related plant pathogenic fungi *Mycosphaerella graminicola* and *Magnaporthe grisea*. *Fungal Genet. Biol*. **46**, 667-68 (2009).

12 Tiley, A. M. M., White, H. J., Foster, G. D. & Bailey, A. M. The ZtvelB gene Is required for vegetative growth and sporulation in the wheat pathogen *Zymoseptoria tritici*. *Front. Microbiol* **10**, 2210 (2019).

13 Mirzadi Gohari, A. *et al.* Molecular characterization and functional analyses of ZtWor1, a transcriptional regulator of the fungal wheat pathogen *Zymoseptoria tritici*. *Mol. Plant Pathol.* **15**, 394-405 (2014).

14 Cairns, T. C. *et al.* Construction and high-throughput phenotypic screening of *Zymoseptoria tritici* over-expression strains. *Fungal Genet. Biol.* **79**, 110-117, (2015).

15 Mohammadi, N. *et al.* The ZtVf1 transcription factor regulates development and virulence in the foliar wheat pathogen *Zymoseptoria tritici*. *Fungal Genet. Biol*. **109** (2017).

16 Habig, M., Bahena-Garrido, S. M., Barkmann, F., Haueisen, J. & Stukenbrock, E. H. The transcription factor Zt107320 affects the dimorphic switch, growth and virulence of the fungal wheat pathogen *Zymoseptoria tritici*. *Mol. Plant Pathol.* **21**, 124-138, (2020).

17 Choi, Y. E. & Goodwin, S. B. MVE1, encoding the velvet gene product homolog in *Mycosphaerella graminicola*, is associated with aerial mycelium formation, melanin biosynthesis, hyphal swelling, and light signaling. *Appl. Environ. Microbiol.* **77**, 942-953 (2011).

18 Yemelin, A. *et al.* Identification of factors involved in dimorphism and pathogenicity of *Zymoseptoria tritici*. *PloS One* **12**, e0183065 (2017).

19 Cousin, A. *et al.* The MAP kinase-encoding gene MgFus3 of the non-appressorium phytopathogen *Mycosphaerella graminicola* is required for penetration and in vitro pycnidia formation. *Mol. Plant Pathol.* **7**, 269-278 (2006).
20 Mehrabi, R., Zwiers, L. H., de Waard, M. A. & Kema, G. H. MgHog1 regulates dimorphism and pathogenicity in the fungal wheat pathogen *Mycosphaerella graminicola*. *Mol. Plant Microbe Interact.* **19**, 1262-1269 (2006).
21 Mehrabi, R., Van der Lee, T., Waalwijk, C. & Gert, H. J. MgSlt2, a cellular integrity MAP kinase gene of the fungal wheat pathogen *Mycosphaerella graminicola*, is dispensable for penetration but essential for invasive growth. *Mol. Plant Microbe Interact.* **19**, 389-398 (2006).
22 Mehrabi, R. & Kema, G. H. Protein kinase A subunits of the ascomycete pathogen *Mycosphaerella graminicola* regulate asexual fructification, filamentation, melanization and osmosensing. *Mol. Plant Pathol.* **7**, 565-577 (2006).
23 Mehrabi, R. *et al.* Gα and Gβ proteins regulate the cyclic AMP pathway that is required for development and pathogenicity of the phytopathogen *Mycosphaerella graminicola*. *Eukaryotic cell* **8**, 1001-1013 (2009).
24 Yemelin, A. *et al.* Two novel dimorphism-related virulence factors of *Zymoseptoria tritici* identified using *Agrobacterium*-mediated insertional mutagenesis. *Int. J. Mol. Sci.* **23**, 400 (2022).
25 King, R. *et al.* A conserved fungal glycosyltransferase facilitates pathogenesis of plants by enabling hyphal growth on solid surfaces. *PLoS Pathog.* **13**, e1006672 (2017).
26 Choi, Y. E. & Goodwin, S. B. Gene encoding a c-type cyclin in *Mycosphaerella graminicola* is involved in aerial mycelium formation, filamentous growth, hyphal swelling, melanin biosynthesis, stress response, and pathogenicity. *Mol. Plant Microbe Interact.* **24**, 469-477 (2011).
27 Schumacher, J. *et al.* The VELVET Complex in the gray mold fungus *Botrytis cinerea*: Impact of BcLAE1 on differentiation, secondary metabolism, and virulence. *Mol. Plant Microbe Interact.* **28**, 659-674 (2015).
28 Ahmed, Y. L. *et al.* The velvet family of fungal regulators contains a DNA-binding domain structurally similar to NF-kappaB. *PLoS Biol.* **11**, e1001750 (2013).
29 Yang, Q., Chen, Y. & Ma, Z. Involvement of BcVeA and BcVelB in regulating conidiation, pigmentation and virulence in *Botrytis cinerea*. *Fungal Genet. Biol.* **50**, 63-71 (2013).
30 Schumacher, J. How light affects the life of *Botrytis*. *Fungal Genet. Biol.* **106**, 26-41 (2017).
31 Boni, A. C. *et al. Neurospora crassa* developmental control mediated by the FLB-3 transcription factor. *Fungal Biol.* **122**, 570-582 (2018).
32 Bailey, L. A. & Ebbole, D. J. The fluffy gene of *Neurospora crassa* encodes a Gal4p-type C6 zinc cluster protein required for conidial development. *Genetics* **148**, 1813-1820 (1998).
33 Bailey-Shrode, L. & Ebbole, D. J. The fluffy gene of *Neurospora crassa* is necessary and sufficient to induce conidiophore development. *Genetics* **166**, 1741-1749 (2004).
34 Seibel, C., Tisch, D., Kubicek, C. P. & Schmoll, M. ENVOY is a major determinant in regulation of sexual development in *Hypocrea jecorina* (*Trichoderma reesei*). *Eukaryotic cell* **11**, 885-895 (2012).
35 Vienken, K. & Fischer, R. The Zn(II)2Cys6 putative transcription factor NosA controls fruiting body formation in *Aspergillus nidulans*. *Mol. Microbiol.* **61**, 544-554 (2006).
36 Sarikaya Bayram, O. *et al.* LaeA control of velvet family regulatory proteins for light-dependent development and fungal cell-type specificity. *PLoS Genet.* **6**, e1001226 (2010).
37 Schumacher, J., de Larrinoa, I. & Tudzynski, B. Calcineurin-responsive zinc finger transcription factor CRZ1 of *Botrytis cinerea* is required for growth, development, and full virulence on bean plants. *Eukaryot. Cell* **7**, 584–601 (2008).
38 Antal, Z. *et al.* The homeobox BcHOX8 gene in *Botrytis cinerea* regulates vegetative growth and morphology. *PloS One* **7**, e48134 (2012).

39 Temme, N. *et al.* BcAtf1, a global regulator, controls various differentiation processes and phytotoxin production in *Botrytis cinerea*. *Mol. Plant Pathol.* **13**, 704-718 (2012).
40 Tao, L. & Yu, J. H. AbaA and WetA govern distinct stages of *Aspergillus fumigatus* development. *Microbiology (Reading)* **157**, 313-326 (2011).
41 Michielse, C. *et al.* The *Botrytis cinerea* Reg1 protein, a putative transcriptional regulator, is required for pathogenicity, conidiogenesis, and the production of secondary metabolites. *Mol. Plant Microbe. Interact.* **24**, 1074–1085 (2011).
42 Cohrs, K. C., Simon, A., Viaud, M. & Schumacher, J. Light governs asexual differentiation in the grey mould fungus *Botrytis cinerea* via the putative transcription factor BcLTF2. *Environ. Microbiol.* **18**, 4068-4086 (2016).
43 Schumacher, J., Simon, A., Cohrs, K. C., Viaud, M. & Tudzynski, P. The transcription factor BcLTF1 regulates virulence and light responses in the necrotrophic plant pathogen *Botrytis cinerea*. *PLoS Genet.* **10**, e1004040 (2014).
44 Brandhoff, B., Simon, A., Dornieden, A. & Schumacher, J. Regulation of conidiation in *Botrytis cinerea* involves the light-responsive transcriptional regulators BcLTF3 and BcREG1. *Curr. Genet.* **63**, 931-949 (2017).
45 Ruiz-Roldán, C., Pareja-Jaime, Y., González-Reyes, J. A. & Roncero, M. I. G. The transcription factor Con7-1 is a master regulator of morphogenesis and virulence in *Fusarium oxysporum*. *Mol. Plant Microbe. Interact.* **28**, 55-68 (2015).
46 Qi, Z. *et al.* MoSwi6, an APSES family transcription factor, interacts with MoMps1 and is required for hyphal and conidial morphogenesis, appressorial function and pathogenicity of *Magnaporthe oryzae*. *Mol. Plant Pathol.* **13**, 677-689 (2012).
47 Martin, R. *et al.* A core filamentation response network in Candida albicans is restricted to eight genes. *PloS One* **8**, e58613 (2013).
48 Martinez-Vazquez, A. *et al.* Identification of the transcription factor Znc1p, which regulates the yeast-to-hypha transition in the dimorphic yeast *Yarrowia lipolytica*. *PloS One* **8** (2013).
49 Jun, S. C., Choi, Y. H., Lee, M. W., Yu, J. H. & Shin, K. S. The putative APSES transcription factor RgdA governs growth, development, toxigenesis, and virulence in *Aspergillus fumigatus*. *mSphere* **5**, e00998 (2020).
50 Rojas, D. A. *et al.* Rrn7 protein, an RNA polymerase I transcription factor, is required for RNA polymerase II-dependent transcription directed by core promoters with a HomolD box sequence. *J. Biol. Chem.* **286**, 26480-26486 (2011).
51 Adams, T. H., Boylan, M. T. & Timberlake, W. E. brlA is necessary and sufficient to direct conidiophore development in *Aspergillus nidulans*. *Cell* **54**, 353-362, (1988).
52 Tan, K. C. & Oliver, R. P. Regulation of proteinaceous effector expression in phytopathogenic fungi. *PLoS Pathogen.* **13**, (2017).
53 Ohara, T. & Tsuge, T. FoSTUA, encoding a basic helix-loop-helix protein, differentially regulates development of three kinds of asexual spores, macroconidia, microconidia, and chlamydospores, in the fungal plant pathogen *Fusarium oxysporum*. *Eukaryot. Cell* **3**, 1412-1422, (2004).
54 Quinlan, A. R. & Hall, I. M. BEDTools: a flexible suite of utilities for comparing genomic features. *Bioinformatics* **26**, 841-842 (2010).
55 Götz, S. *et al.* High-throughput functional annotation and data mining with the Blast2GO suite. *Nucleic Acids Res.* **36**, 3420-3435 (2008).
56 El-Gebali, S. *et al.* The Pfam protein families database in 2019. *Nucleic Acids Res.* **47**, D427-D432 (2019).
57 Almagro Armenteros, J. J. *et al.* SignalP 5.0 improves signal peptide predictions using deep neural networks. *Nat. Biotechnol.* **37**, 420-423 (2019).
58 Sperschneider, J., Dodds, P. N., Gardiner, D. M., Singh, K. B. & Taylor, J. M. Improved prediction of fungal effector proteins from secretomes with EffectorP 2.0. *Mol. Plant Pathol.* **19**, 2094-2110 (2018).
59 Grandaubert, J., Bhattacharyya, A. & Stukenbrock, E. H. RNA-seq-based gene annotation and comparative genomics of four fungal grass pathogens in the genus *Zymoseptoria* identify novel orphan genes and species-specific invasions of transposable elements. *G3 (Bethesda)* **5**, 1323-1333 (2015).

60 Möller, S., Croning, M. D. R. & Apweiler, R. Evaluation of methods for the prediction of membrane spanning regions. *Bioinformatics* **17**, 646-653 (2001).
61 Käll, L., Krogh, A. & Sonnhammer, E. L. A combined transmembrane topology and signal peptide prediction method. *J. Mol. Biol.* **338**, 1027-1036 (2004).
62 Needleman, S. B. & Wunsch, C. D. A general method applicable to the search for similarities in the amino acid sequence of two proteins. *J. Mol. Biol.* **48**, 443-453, (1970).
63 Sievers, F. *et al.* Fast, scalable generation of high-quality protein multiple sequence alignments using Clustal Omega. *Mol. Syst. Biol.* **7**, 539 (2011).
64 Martin, M. Cutadapt removes adapter sequences from high-throughput sequencing reads. *EMBnet. journal* **17**, 10-12 (2011).
65 Wingett, S. W. & Andrews, S. FastQ Screen: A tool for multi-genome mapping and quality control. *F1000Research* **7**, 1338 (2018).
66 Kim, D. *et al.* TopHat2: accurate alignment of transcriptomes in the presence of insertions, deletions and gene fusions. *Genome Biol.* **14**, R36 (2013).
67 Anders, S., Pyl, P. T. & Huber, W. HTSeq--a Python framework to work with high-throughput sequencing data. *Bioinformatics* **31**, 166-169 (2015).
68 Love, M. I., Huber, W. & Anders, S. Moderated estimation of fold change and dispersion for RNA-seq data with DESeq2. *Genome Biol.* **15**, 550 (2014).
69 Fornes, O. *et al.* JASPAR 2020: update of the open-access database of transcription factor binding profiles. *Nucleic Acids Res.* **48**, D87-D92 (2020).
70 Grant, C. E., Bailey, T. L. & Noble, W. S. FIMO: scanning for occurrences of a given motif. *Bioinformatics* **27**, 1017-1018 (2011).
71 Li, H. & Durbin, R. Fast and accurate short read alignment with Burrows-Wheeler transform. *Bioinformatics* **25**, 1754-1760 (2009).
72 Li, H. *et al.* The Sequence Alignment/Map format and SAMtools. *Bioinformatics* **25**, 2078-2079 (2009).
73 Robinson, J. T. *et al.* Integrative genomics viewer. *Nat. Biotechnol.* **29**, 24-26 (2011).
