# Supplementary material for: Zymoseptoria tritici white-collar complex integrates light, temperature and plant cues to initiate dimorphism and pathogenesis
Source: Nat Commun. 2022 Sep 26;13:5625. doi: 10.1038/s41467-022-33183-2 (PMC9512790; doi:10.1038/s41467-022-33183-2)
Supplement: Supplementary file 13 — Reporting Summary [file 41467_2022_33183_MOESM13_ESM.pdf]

nature portfolio

Corresponding author(s): Prof Gero Steinberg

Last updated by author(s): Sep 3, 2022

Reporting Summary

Nature Portfolio wishes to improve the reproducibility of the work that we publish. This form provides structure for consistency and transparency in reporting. For further information on Nature Portfolio policies, see our Editorial Policies and the Editorial Policy Checklist.

Statistics

For all statistical analyses, confirm that the following items are present in the figure legend, table legend, main text, or Methods section.

n/a	Confirmed	
☐	☒	The exact sample size (*n*) for each experimental group/condition, given as a discrete number and unit of measurement
☐	☒	A statement on whether measurements were taken from distinct samples or whether the same sample was measured repeatedly
☐	☒	The statistical test(s) used AND whether they are one- or two-sided<br>*Only common tests should be described solely by name; describe more complex techniques in the Methods section.*
☐	☒	A description of all covariates tested
☐	☒	A description of any assumptions or corrections, such as tests of normality and adjustment for multiple comparisons
☐	☒	A full description of the statistical parameters including central tendency (e.g. means) or other basic estimates (e.g. regression coefficient) AND variation (e.g. standard deviation) or associated estimates of uncertainty (e.g. confidence intervals)
☐	☒	For null hypothesis testing, the test statistic (e.g. *F*, *t*, *r*) with confidence intervals, effect sizes, degrees of freedom and *P* value noted<br>*Give P values as exact values whenever suitable.*
☒	☐	For Bayesian analysis, information on the choice of priors and Markov chain Monte Carlo settings
☒	☐	For hierarchical and complex designs, identification of the appropriate level for tests and full reporting of outcomes
☒	☐	Estimates of effect sizes (e.g. Cohen's *d*, Pearson's *r*), indicating how they were calculated

Our web collection on statistics for biologists contains articles on many of the points above.

Software and code

Policy information about availability of computer code

Data collection	MetaMorph 7.8x; Leica LAS X 3.5.2.18963; VisiView 3.3.0.4
Data analysis	MetaMorph 7.8x; Leica LAS X 3.5.2.18963; ImageJ-win64; GraphPad Prism Version 6 and Version 9; Bedtools 2.26.0; Blast2GO 5.0; Pfam 32.0; SignalP 5.0; EffectorP 2.0; FungiDB Release 46; TMHMM 2.0; Phobius 1.01; EMBOSS Needle 6.6.0.; CLUSTAL Omega 1.2.4.; Cutadapt 1.13; FastQC 0.11.4; FastQScreen 0.5.2; TopHat2 2.1.1; HTSeq-count 0.10.0; DESeq2 1.14.1; JASPAR release 8; FIMO 5.1.0; MEGA5.2; BWA-mem 0.7.15; Picard 2.12.1; Samtools 1.3.1; Seqtk 1.2; IGV 2.4.10.; MassHunter Quantitative Analysis Library Editor (v10.0); Agilent MassHunter Qualitative Analysis (v10.0); Agilent Unknowns Analysis (v10.0); IGV 2.4.10; MEGA 5.2; R version 4.0.1.

For manuscripts utilizing custom algorithms or software that are central to the research but not yet described in published literature, software must be made available to editors and reviewers. We strongly encourage code deposition in a community repository (e.g. GitHub). See the Nature Portfolio guidelines for submitting code & software for further information.

Data

Policy information about availability of data

All manuscripts must include a data availability statement. This statement should provide the following information, where applicable:

- Accession codes, unique identifiers, or web links for publicly available datasets
- A description of any restrictions on data availability
- For clinical datasets or third party data, please ensure that the statement adheres to our policy

The authors confirm that all relevant data are included in the paper or in the Supplementary Information file. Source data, covering the minimum dataset necessary to interpret, verify and extend the research in the article, can be accessed in the Source Data file, provided with this paper, or are accessible from the links provided herein. Raw sequencing data are available from the NCBI Sequence Read Archive (https://www.ncbi.nlm.nih.gov/bioproject/PRJNA759417), 3-hourly canopy

temperate data are available from JRA-55 via Research Data Archive (https://rda.ucar.edu/datasets/ds628.0/). The IPO323 reference genome data is accessible at https://fungi.ensembl.org/Zymoseptoria_tritici/Info/Index.

Field-specific reporting

Please select the one below that is the best fit for your research. If you are not sure, read the appropriate sections before making your selection.

- [x] Life sciences
- [ ] Behavioural & social sciences
- [ ] Ecological, evolutionary & environmental sciences

For a reference copy of the document with all sections, see nature.com/documents/nr-reporting-summary-flat.pdf

Life sciences study design

All studies must disclose on these points even when the disclosure is negative.

Sample size	Sample sizes ranged from 4 experiments to several hundred cells. The sample size followed practicality issues, which reflect the ability to gather data given the degree of difficulty of the experimental system. The smallest sample sizes were n=4, which allows Student's t-testing (J.C.F. de Winter (2013), "Using the Student's t-test with extremely small sample sizes," Practical Assessment, Research and Evaluation, 18:10, August, ISSN 1531-7714); the sample sizes of climate data were determined by the selected areas of study and the spatial resolution of the climate data.
Data exclusions	Area grid cells were removed from the climate data accessed from JRA-55 that were not suitable for the study i.e., grid cells located in neighbouring or nearby countries, or grid cells outside of the target region of study.
Replication	Experiments were repeated using independently grown cell cultures, using newly prepared media, or plant infection rounds, using freshly grown plants and microbes. Consequently, examination of the experimental outcome was done at different time points on independently grown biological material; all images included in the figures show representative examples, all results obtained in 2 to >5 experiments (as stated in the figure legends of the main text) were included in the provided quantifications. Thus, all experiments described in this paper were performed with 2-5 independent biological replicates, usually including 5-10 technical replicates each.
Randomization	An experimental group was defined by the fungal cells in an examined cell culture flask or by plants inoculated in a plant pathogenicity assay experiment (e.g. all 3rd leaves of 10 plants inoculated at the same day). Cells within this experimental group were measured from randomly chosen microscopic fields of view. This procedure was repeated for each biological replicate.
Blinding	Blinding appears not to be applicable, as blinding is usually done in clinical research, where "treatment allocation for each patient is not revealed until the patient has irrevocably been entered into the trial" (Day 2000, pBMJ 2000;321:504). However, we reduced the bias of data collection by strictly analysing and including all cells in a randomly chosen microscopic field (exception: cells were collapsed and dead) or investigating all areas of an inoculated and infected plant leaf (exception in pathogenicity assays: leaves died before developing disease symptoms), always using biological and technical replicates.

Reporting for specific materials, systems and methods

We require information from authors about some types of materials, experimental systems and methods used in many studies. Here, indicate whether each material, system or method listed is relevant to your study. If you are not sure if a list item applies to your research, read the appropriate section before selecting a response.

Materials & experimental systems

n/a	Involved in the study	
x		Antibodies
x		Eukaryotic cell lines
x		Palaeontology and archaeology
x		Animals and other organisms
x		Human research participants
x		Clinical data
x		Dual use research of concern

Methods

n/a	Involved in the study	
x		ChIP-seq
x		Flow cytometry
x		MRI-based neuroimaging
